# Switchable divergent synthesis of chiral indole derivatives *via* catalytic asymmetric dearomatization of 2,3-disubstituted indoles†

**DOI:** 10.1039/d4ra03231d

**Published:** 2024-05-14

**Authors:** Tingting Liu, Jianbin Wang, Rou Xiao, Junling Zhao

**Affiliations:** a School of Pharmaceutical Sciences (Shenzhen), Shenzhen Campus of Sun Yat-sen University Shenzhen 518107 P. R. China zhaojling3@mail.sysu.edu.cn

## Abstract

A strategy allowing the switchable divergent synthesis of chiral indole derivatives was established *via* chiral phosphoric acid-catalyzed asymmetric dearomatization of 2,3-disubstituted indoles using naphthoquinone monoimines as electrophiles. The products were switched between chiral indolenines and fused indolines according to the post-processing conditions. Both two types of products were obtained in good to high yields with generally excellent enantioselectivities. NaBH_4_ was found to work as a promoter as well as a reductant in the cyclization process leading to fused indolines.

## Abstract 2

A CPA-catalyzed switchable divergent synthesis of chiral indolenines/indolines *via* the CADA of 2,3-disubstituted indoles has been described. The post-processing conditions played a key role for the structure switch of the final products.

## Graphical abstract

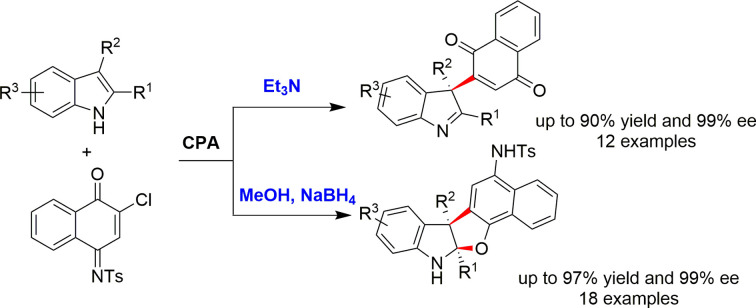

## Introduction

The indole ring system is one of the most intriguing nitrogen-containing heterocycles because of its frequent appearance in natural products and pharmaceuticals.^1^ Therefore, the synthesis of indole derivatives has garnered a lot of attention from both academic and industrial realms. The direct functionalization of the indole core is the most direct and efficient strategy to accessing indole derivatives and intensive efforts have been devoted to this end. Among which, the catalytic asymmetric dearomatization (CADA) of 3-substituted indoles^2^ is particularly attractive because the resulting products are indolenines^3^ or fused indolines^4^ that are found in a number of natural alkaloids and bioactive molecules. In this respect, many elegant methods have been developed using various catalytic strategies, such as propargylic substitution,^5^ allylic alkylation,^6^ Michael addition,^7^ halogenation,^8^ hydrazination,^9^ and arylation.^10,11^

**Scheme 1:**
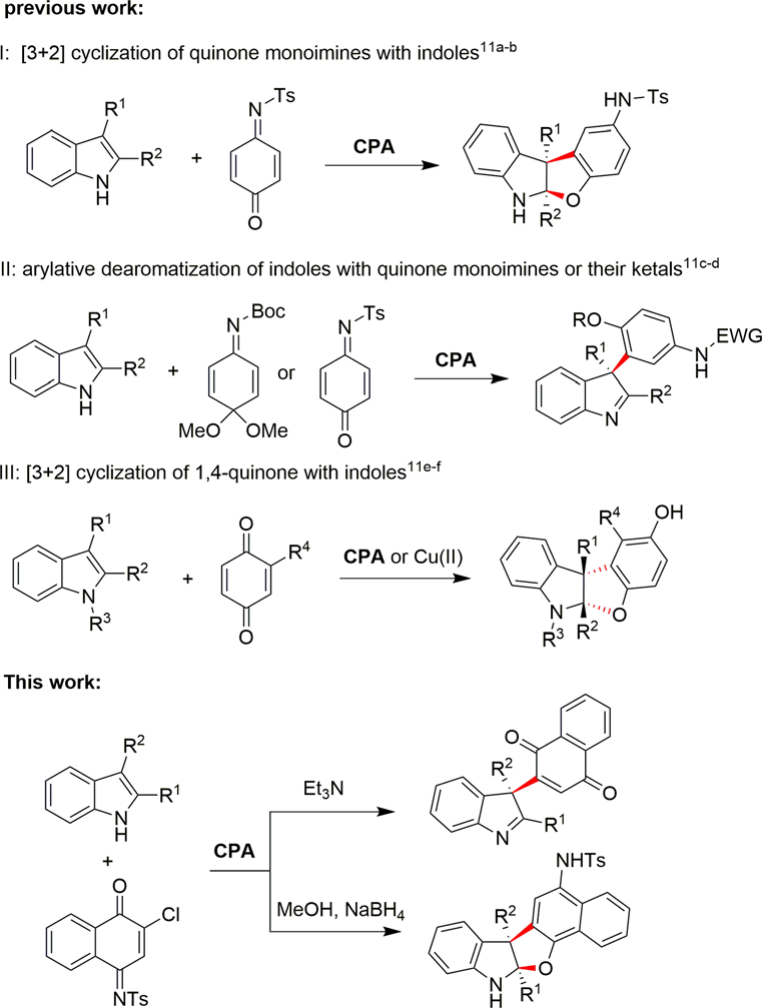
Asymmetric arylative dearomatization of indoles using quinones and their imines as electrophiles.

With regard to the asymmetric arylation strategy, electrophilic quinones and their imines are excellent acceptors, and some elegant methods have been developed. As a class of privileged organocatalyst, chiral phosphoric acids (CPAs) showed the best catalytic efficiency in those transformations.^12^ Zhang and co-workers^11*a*^ reported CPA-catalyzed asymmetric arylative dearomatization/cyclization of 3-substituted indoles with 1,4-quinone monoimines, affording chiral benzofuroindolines with high yields and stereoselectivities. The 1,4-quinone monoimines used can be one-pot generated though oxidation of the corresponding phenols as reported by Zhong group, who employed a biomimetic Mn(iii)/CPA relay catalysis strategy for this process.^11*b*^ On the other hand, Shi group revealed the CADA of 2, 3-disubstituted indoles with quinone derived imine ketals or monoimines to give chiral indolenines.^11*c*,*d*^ Recently, the synthesis of fused indolines *via* asymmetric [3 + 2] annulation of 1,4-quinones with indoles was also reported by Tang^11*e*^ and Zhong,^11*f*^ respectively (Scheme 1).

As the analogs of quinones, naphthoquinones and their derivatives often displayed similar chemical reactivity compared to that of quinones. However, that is not the case with respect to the CADA of indoles, and no successful example was given using naphthoquinones or their imines as electrophiles so far. This fact suggested that there might be challenges need to be overcome when naphthoquinones were used. This was exemplified by the CPA-catalyzed asymmetric [3 + 2] annulation of 1,4-quinones with indoles. Probably due to its relatively lower electrophilicity, no reaction occurred with 1,4-quinone being replaced by 1,4-napthoquinone as reported by Zhong.^11*f*^ As our continuing interest in the asymmetric functionalization of indoles,^13^ here we presented our recent study on the CADA of 2,3-disubstituted indoles using naphthoquinone monoimines as electrophiles.

**Scheme 2:**
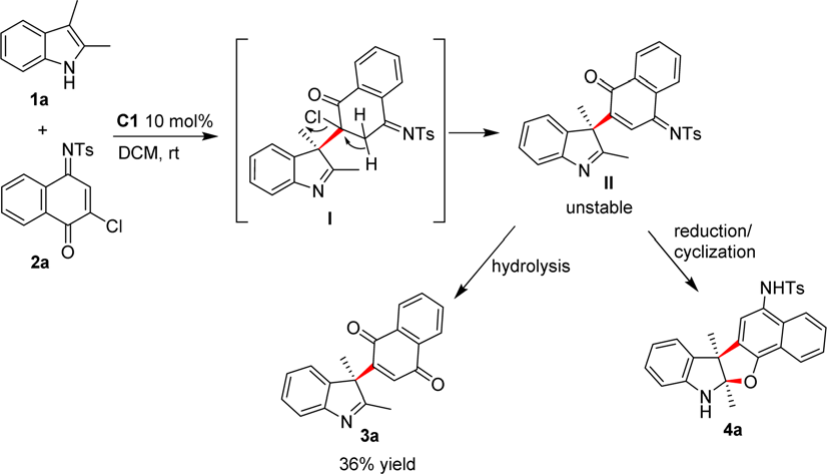
Design of the switchable divergent synthesis of chiral indole derivatives.

We started our investigation by reacting 2,3-dimethylindole (1a) with naphthoquinone monoimine (2a) in dichloromethane (DCM) at room temperature with 10 mol% C1 as a catalyst. Not surprisingly, a mixture of hard-to-separate products was produced. Fortunately, we isolated the dechlorinated indolenine derivative 3a in a low yield, and found out that the other products were slowly transformed to 3a during the separation process. These results suggested that unstable intermediates were generated during this dearomatization process. Based on the above results, we assumed that the dearomatization of 1a produced intermediate I, which underwent dehydrochlorination to give intermediate II. II was sensitive to moisture and hydrolyzation occurred during the purification process to give 3a. Based on this assumption, the addition of a base might accelerate the process leading to 3a, while the reduction of carbonyl group in II might afford indoline derivative 4a. Thus, a switchable divergent synthesis of chiral indole derivatives might be established by simply regulating post-processing conditions (Scheme 2).

To confirm our hypothesis, trimethylamine was added to the above reaction mixture after completion by TLC (Method A), and the yield of 3a was increased to 95%. On the other hand, indoline derivative 4a was formed smoothly in 80% yield following the treatment of NaBH_4_ (Method B). However, products with low enantioselectivities were observed in both cases (Table 1, entries 1 and 2). The reaction leading to 3a was chosen as a model reaction and a range of CPAs were subsequently examined to improve the stereocontrol of this transformation. As shown in Table 1, it was found that both the substituents and the chiral backbones of the catalysts have remarkable effects on the yield and enantioselectivity of the product. Among these catalysts tested, C10 showed the best catalytic efficiency to give 3a in 95% yield and 84% ee (Table 1, entry 11). The enantioselectivity of 3a was further improved to 94% ee when 1,2-dichloroethane (DCE) was used as the reaction media (Table 1, entry 14). The best result in term of yield and ee was obtained with the addition of 4 Å molecular sieves as an additive, albeit a prolonged reaction time was needed (Table 1, entry 15). When the post-processing condition was switched to B: with the addition of NaBH_4_ and MeOH, the corresponding indoline derivative 4a was produced in 93% yield and 99% ee (Table 1, entry 16). Thus, we have developed a method for the switchable chiral indolenines/indolines synthesis by simply switching the post-processing conditions of the reaction.

**Table 1:** Optimization of the Reaction Conditions^a^

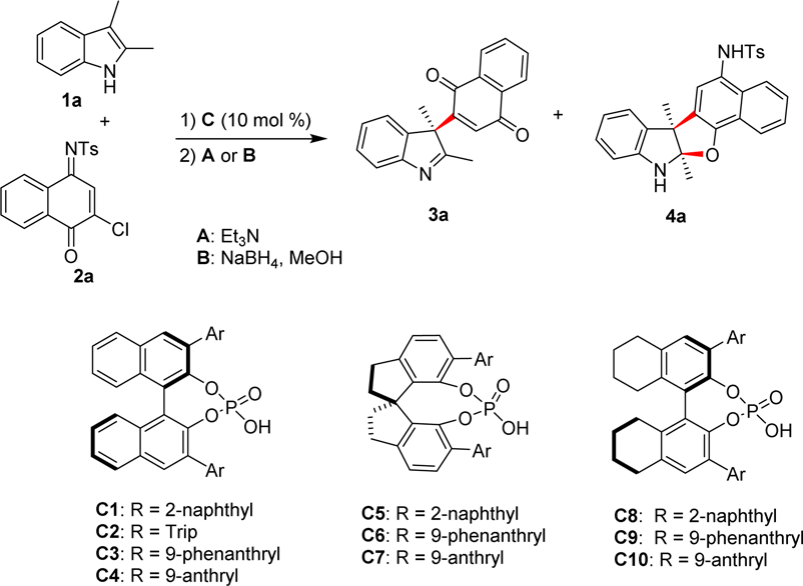					
Entry	CPA	Solvent	A/B	Yield b(%)	ee^c^ (%)
1	C1	DCM	A	3a, 95	30
2	C1	DCM	B	4a, 80	33
3	C2	DCM	A	3a, 95	8
4	C3	DCM	A	3a, 95	76
5	C4	DCM	A	3a, 71	72
6	C5	DCM	A	3a, 44	22
7	C6	DCM	A	3a, 62	57
8	C7	DCM	A	3a, 46	32
9	C8	DCM	A	3a, 92	31
10	C9	DCM	A	3a, 95	82
11	C10	DCM	A	3a, 95	84
12	C10	THF	A	3a, 51	21
13	C10	PhMe	A	3a, 97	84
14	C10	DCE	A	3a, 88	94
15^d^^,^^e^	C10	DCE	A	3a, 89	98
16^d^^,^^e^	C10	DCE	B	4a, 93	99

a Reaction conditions: 1a (0.05 mmol), 2a (0.075 mmol), C (0.005 mmol), solvent (0.5 mL), room temperature, 2 h, unless otherwise noted. Condition A: 0.1 mL Et_3_N was added and the reaction was stirred in air for an extra 30 min. Condition B: MeOH (0.5 mL) and NaBH_4_ (0.5 mmol) were added and the reaction was stirred for an extra 30 min.

b Isolated yields were given.

c Enantiomeric excess was determined by HPLC on a chiral stationary phase. All dr > 20 : 1 determined by ^1^H NMR spectra analysis.

d 20 mg 4 Å molecular sieves was added.

e Reaction time is 6 h.

With the optimal reaction conditions determined, we first studied the scope of the reaction leading to chiral indolenine derivatives 3, and the results were presented in Table 2. It was revealed that substituents variations on the benzene position of indoles were well tolerated, producing the corresponding products in good to high yields (53–90%) with excellent enantioselectivities (97–99% ee). Then, we turned our attention to the reaction using other naphthoquinone monoimines. The reaction proceeded smoothly when brominated substrate 2b was used, expectedly, debromination occurred to produce 3a in comparably high yield and ee. The reaction of unsubstituted imine 2c was also examined. In this case, the intermediate should be a naphthol derivative III which tend to undergo cyclization to give fused indoline 4a. However, high yield of 3a was still obtained following the addition of Et_3_N. This result suggested that the cyclization process was much slower than expected and oxidation/hydrolyzation occurred quickly under basic condition.^14^

**Table 2:** Substrate scope for the synthesis of chiral indolenines^a^

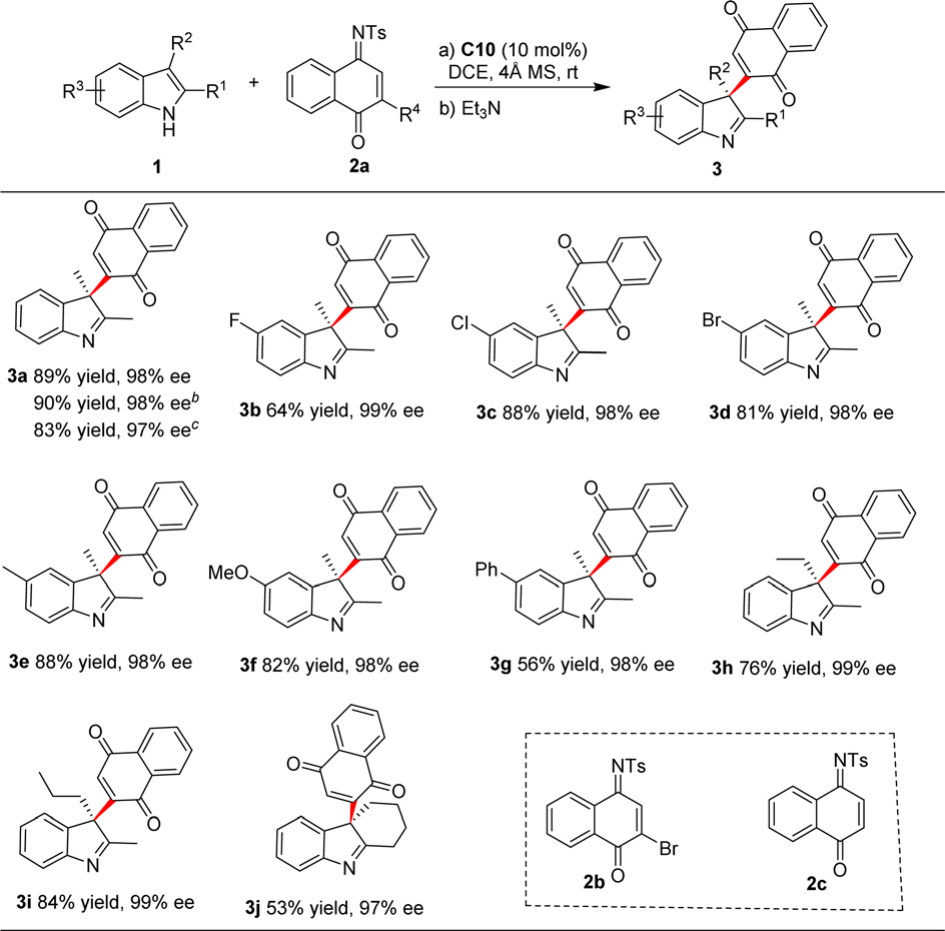

a Reactions conditions: 1 (0.05 mmol), 2 (0.075 mmol), C10 (0.005 mmol), 20 mg 4 Å molecular sieves, DCE (0.5 mL), room temperature, 6–46 h. After completion, 0.1 mL Et_3_N was added, and the reaction mixture was stirred in air for 30 min. Isolated yields are given. Enantiomeric excess was determined by HPLC on a chiral stationary phase.

b 2b was used as an electrophile.

c 2c was used as an electrophile.

Then, the scope for the synthesis of fused indoline derivatives 4 was examined (Table 3). The reaction between various 2,3-disubstituted indoles and naphthoquinone monoimines were investigated under the standard conditions, and the results indicated that the change of post-processing conditions has little effect on the efficiency of this reaction, affording the corresponding fused indolines 4 smoothly with good outcomes. It seems that NaBH_4_ played multiple roles in this reaction: (1) as a reducing agent, (2) as a “promoter” to accelerate the cyclization process.^15^ This point was further confirmed by the reaction of 2c. In this case, cyclization product 4a was not observed in the absence of NaBH_4_, while high yield (77%) of 4a was obtained in just 10 minutes following the addition of this reagent.^14^

**Table 3:** Substrate scope for the synthesis of fused indolines^a^

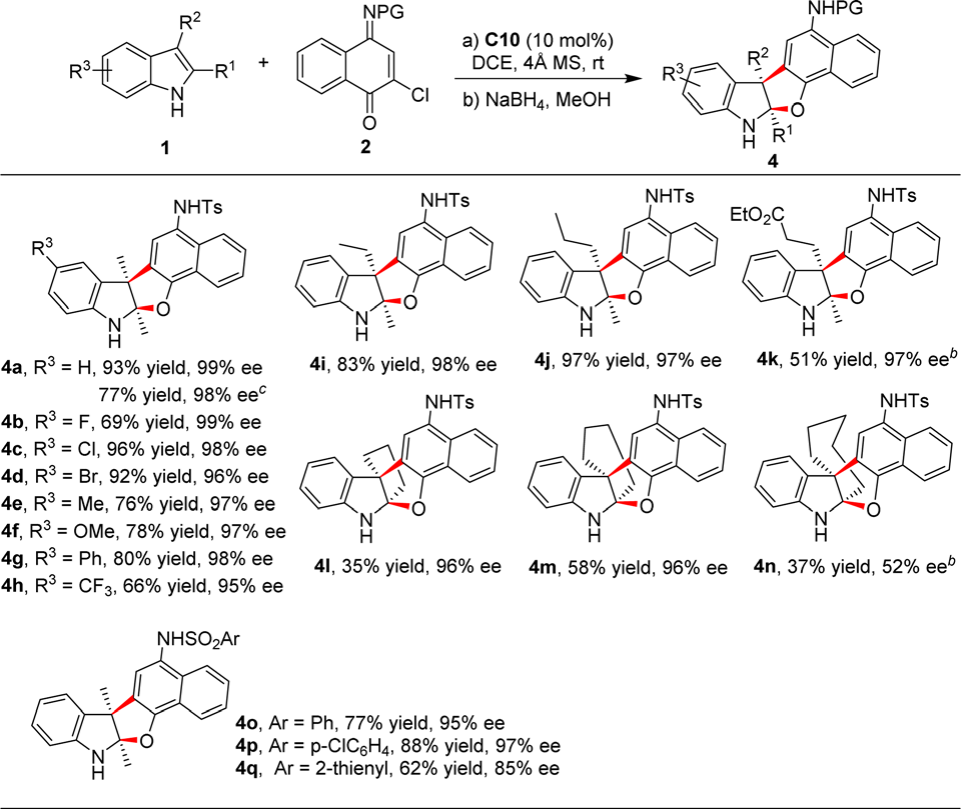

a Reactions conditions: 1 (0.05 mmol), 2 (0.075 mmol), C10 (0.005 mmol), 20 mg 4 Å molecular sieves, DCE (0.5 mL), room temperature, 4–79 h. After completion, 0.5 mL MeOH and 0.5 mmol NaBH_4_ (in portions) was added, and the reaction mixture was stirred for 30 min. Isolated yields are given. Enantiomeric excess was determined by HPLC on a chiral stationary phase. dr > 20 : 1 in all cases.

b Without the addition of molecular sieves.

c 2c was used as an electrophile.

The absolute configurations of the newly formed chiral centers in 4a were assigned as 2*R*, 3*S* by X-ray analysis of its methylated product 5 (for details, see the ESI†).^16^ According to this observation, the chiral quaternary center in 3 has a *S* configuration. That is because the synthesis of these two types of chiral indole derivatives originated from the same asymmetric dearomatization reaction and the only difference is the post-processing procedure which will not influence the configuration of the existing quaternary chiral center at the C3-position of indole nucleus.

**Scheme 3:**
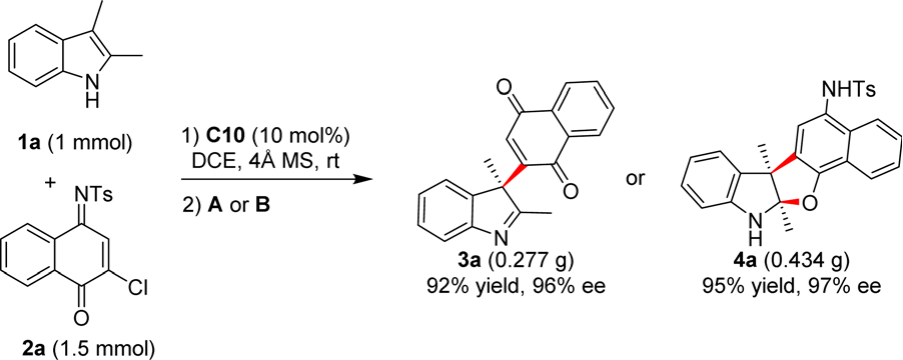
Scale-up reactions for the syntheses of 3a and 4a.

The synthetic potential of this reaction was also explored. When the model reactions were up scaled to 1 mmol under the standard conditions, high yields of 3a or 4a were obtained, respectively, with slightly decreased enanioselectivities (Scheme 3).

**Scheme 4:**
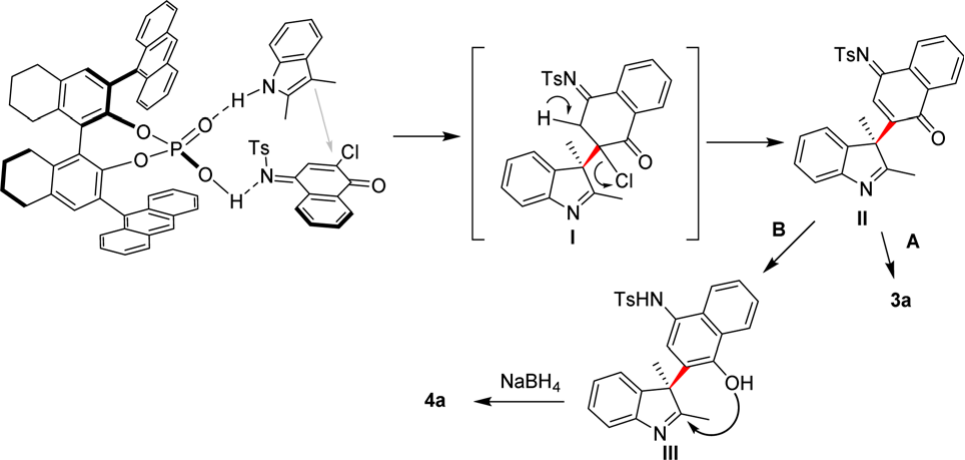
Possible reaction mechanism.

Finally, the possible reaction mechanism was proposed to explain the stereochemistry of this reaction. As shown in Scheme 4, both substrates were activated through hydrogen bonding interaction with the catalyst, and 1a attack 2a from the bottom to give indolenine intermediate I bearing a quaternary chiral centre in *S* configuration. The addition of NaBH_4_ produced III which underwent cyclization to give 4a, Re face attack was favoured during this process to generate the second quaternary chiral centre in *R* configuration.

## Conclusions

In conclusion, we have developed a protocol allowing the switchable divergent synthesis of chiral indolenines/fused indolines *via* a CPA-catalyzed dearomatization of 2,3-disubstituted indoles with naphthoquinone monoimines. Unlike their quinone-derived counterparts, the reaction of naphthoquinone monoimines with indoles produced unstable intermediates which can be readily transformed to different products by simply using different post-processing conditions. In the case for the synthesis of fused indolines, NaBH_4_ was used as a reducing agent as well as a promoter in the cyclization process.

## Author contributions

J. Z. conceived and directed the project. T. L., J. W. and R. X. conducted the experimental work and data analysis. J. Z. wrote the manuscript. All authors approved the final version of the manuscript.

## Conflicts of interest

There are no conflicts to declare.

## Supplementary Material

RA-014-D4RA03231D-s001

RA-014-D4RA03231D-s002
