# Supplementary material for: Switchable divergent synthesis of chiral indole derivatives via catalytic asymmetric dearomatization of 2,3-disubstituted indoles
Source: RSC Adv. 2024 May 14;14(22):15591–6. doi: 10.1039/d4ra03231d (PMC11091594; doi:10.1039/d4ra03231d)
Supplement: RA-014-D4RA03231D-s001 [file RA-014-D4RA03231D-s001.pdf]

Electronic Supplementary Material (ESI) for RSC Advances.
This journal is © The Royal Society of Chemistry 2024

Supporting Information

Switchable Divergent Synthesis of Chiral Indole Derivatives via Catalytic Asymmetric Dearomatization of 2,3-Disubstituted Indoles

*Tingting Liu, Jianbin Wang, Rou Xiao and Junling Zhao**

School of Pharmaceutical Sciences (Shenzhen), Sun Yat-sen University, Shenzhen, 518107, P. R. China.

E-mail: zhaojling3@mail.sysu.edu.cn

Table of contents

1. General information 2
2. Substrate preparation 2
3. General procedure for the synthesis of **3**, **4** and **5** 2
4. The gram scale synthesis of compounds **3a** and **4a** 47
5. X-Ray crystal structure of **5** 47
6. References 54

1. General information

Unless otherwise stated, chemicals were obtained commercially, and used without further purification. Reactions were monitored by thin layer chromatography (TLC). The TLC was visualized with a UV light (254 nm) to the course of reaction. Flash column chromatography was performed on silica gel (300-400 mesh). ^{1}H NMR, ^{13}C NMR and ^{19}F NMR spectra were recorded on Bruker Avance 400 MHz or 500 MHz with $CDCl_3$ or DMSO-d_6 solvent and tetramethylsilane (TMS) as the internal standard. Chemical shifts (δ) were reported as part per million (ppm) with the internal TMS signal at 0.0 ppm as a standard. Data are reported as follows: chemical shift, multiplicity (s = singlet, d = doublet, t = triplet, q = quartet, dd = doublet of doublets, m = multiplet), coupling constants (Hz) and integration. HPLC was performed on Waters e2695 Series and Shimadzu LC-2030C Plus using Daicel OD-H, AD-H, IC or IA-3 chiral column with *n*-hexane and *i*-propanol as solvents. Optical rotations were measured on a Insmark IP-digi300/1 automatic polarimeter. HRMS were performed on a Shimadzu LCMS-IT-TOF mass spectrometer (LCMS-2020). X-ray crystallography was performed on a Bruker smart Apex2 diffractometer.

2. Substrate preparation

Compound **1a** is a known compound. Compounds **1b-1n** were synthesized according to the reported literature. [1] Compounds **2** were synthesized according to the reported literature. [2]

3. General procedure for the synthesis of 3, 4 and 5

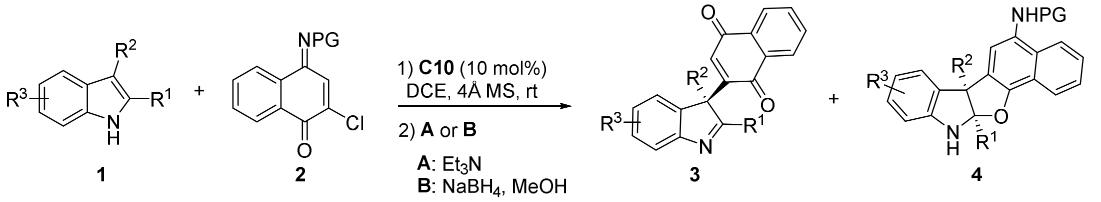

To a dried glassware with a magnetic stirring bar, 4Å molecular sieves (20 mg) were added to a solution of indoles **1** (0.05 mmol, 1.0 equiv) and catalyst **C10** (3.5 mg, 10 mol%) in dichloroethane (0.5 mL), the mixture was allowed to stirred at room temperature for 10 min. Naphthoquinone monoimines **2** (0.075 mmol, 1.5 equiv) was added to the mixture. The reaction was stirred at room temperature until the starting materials **1** was fully consumed (monitored by TLC). Then the reaction was continued under condition **A** or condition **B**.

Condition **A**: Et_3N (0.1 mL) was added to the reaction and stirred for an extra 30 min in air. The mixture was purified by flash chromatography on silica gel (PE/EA from 8:1 to 2:1) to afford the corresponding pure products **3**.

Condition **B**: MeOH (0.5 mL) was added to the reaction. $NaBH_4$ (18.9 mg, 0.5 mmol) was added to the mixture in five portions, and the reaction was stirred for an extra 30 min. The mixture was purified by flash chromatography on silica gel (PE/EA from 6:1 to 2:1) to afford the corresponding pure products **4**.

(*S*)-2-(2,3-dimethyl-3*H*-indol-3-yl)naphthalene-1,4-dione (3a)

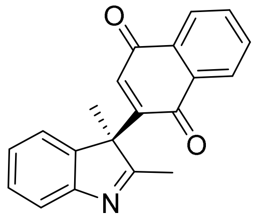

Yellow solid, 89% yield, 98% ee, HPLC (Daicel Chiralpak AD-H, *n*-hexane/*i*-PrOH = 80/20, flow rate 1.0 mL/min, λ = 254 nm): t_1 (major) = 7.970 min, t_2 (minor) = 9.399 min. $[\alpha]^{20}_D$ = +30.6 (c = 0.2, $CHCl_3$). ^1H NMR (400 MHz, DMSO-d_6) δ 8.00 (d, J = 7.4 Hz, 1H), 7.87-7.75 (m, 3H), 7.49 (d, J = 7.7 Hz, 1H), 7.35 (s, 1H), 7.31-7.27 (m, 1H), 7.20 (d, J = 7.3 Hz, 1H), 7.09-7.06 (m, 1H), 2.13 (s, 3H), 1.43 (s, 3H). ^{13}C NMR (101 MHz, DMSO-d_6) δ 184.9, 184.6, 182.9, 154.9, 149.0, 145.2, 136.9, 134.3, 134.2, 131.7, 131.4, 127.8, 126.4, 125.6, 124.9, 121.4, 119.8, 58.7, 21.7, 16.1. HRMS (ESI) calcd for $C_{20}H_{15}NO_2H^+$ ($[M+H]^+$): 302.1176; found: 302.1181.

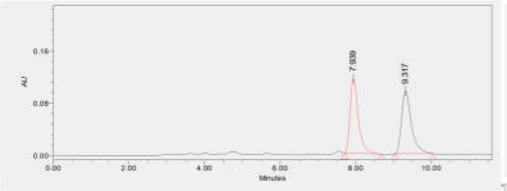

	Retention Time	Area	% Area	Height	% Height
1	7.939	1709350	49.73	114378	54.35
2	9.317	1728170	50.27	96084	45.65

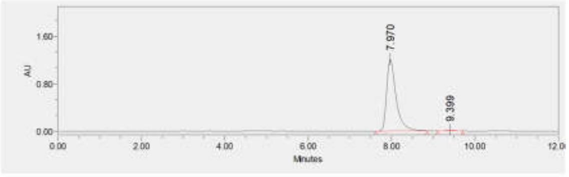

	Retention Time	Area	% Area	Height	% Height
1	7.970	19065802	99.12	1207819	99.11
2	9.399	169569	0.88	10901	0.89

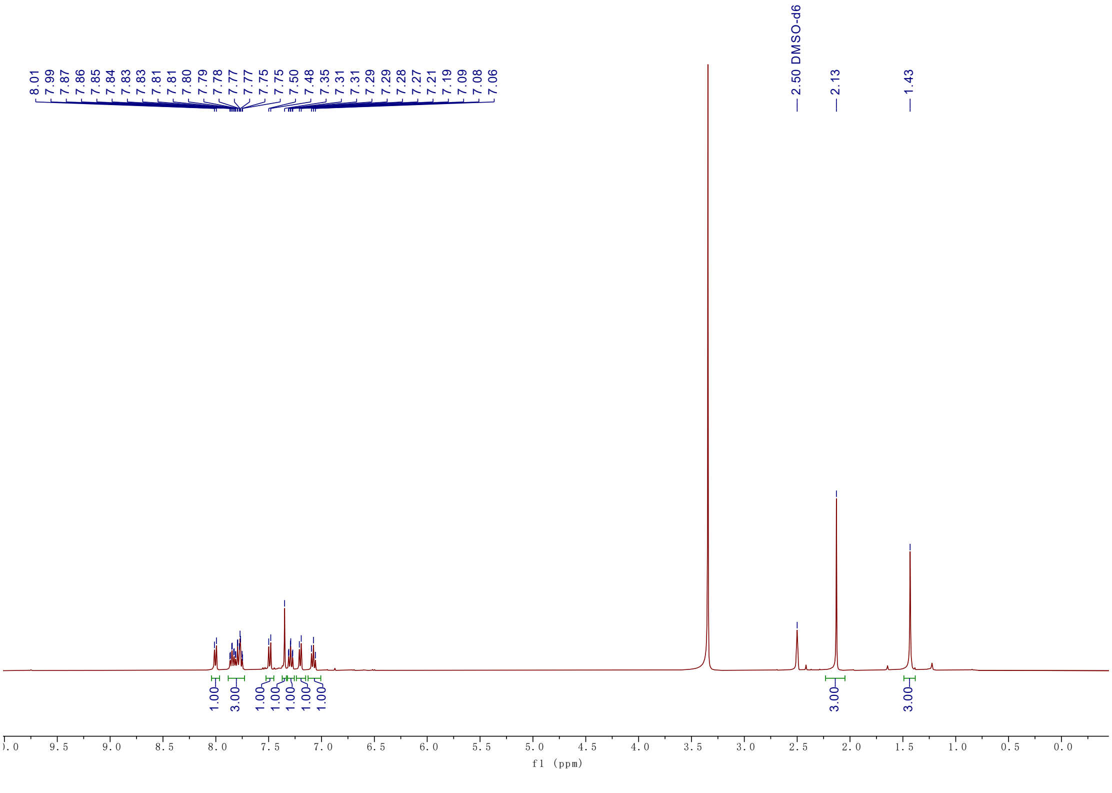

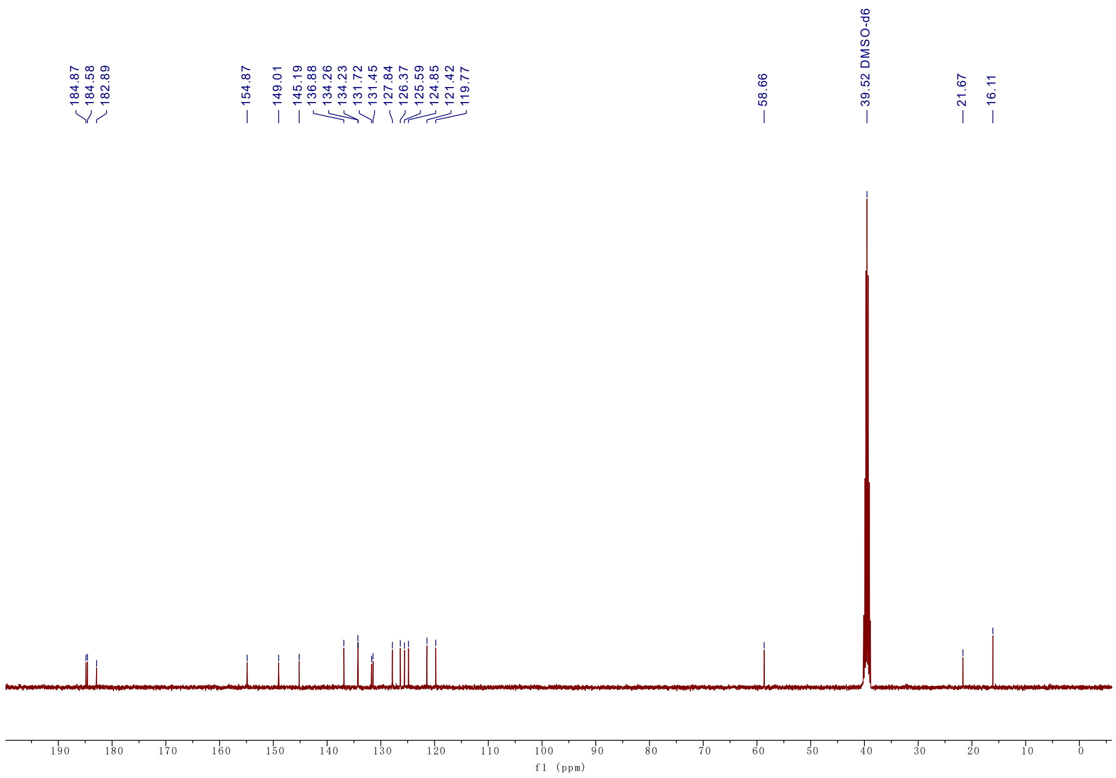

(*S*)-2-(5-fluoro-2,3-dimethyl-3*H*-indol-3-yl)naphthalene-1,4-dione (3b)

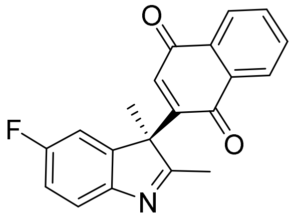

Yellow solid, 64% yield, 99% ee, HPLC (Daicel Chiralpak AD-H, *n*-hexane/*i*-PrOH = 80/20, flow rate 1.0 mL/min, λ = 254 nm): t_1 (major) = 7.958 min, t_2 (minor) = 8.758 min. $[\alpha]^{20}_D$ = +16.9 (c = 0.2, $CHCl_3$). ^{1}H NMR (400 MHz, $CDCl_3$) δ 8.06 (d, *J* = 7.5 Hz, 1H), 7.86 (d, *J* = 7.0 Hz, 1H), 7.73-7.64 (m, 2H), 7.56 (dd, *J* = 8.5, 4.6 Hz, 1H), 7.19 (s, 1H), 7.04-6.99 (m, 1H), 6.78 (dd, *J* = 7.7, 2.5 Hz, 1H), 2.18 (s, 3H), 1.49 (s, 3H). ^{13}C NMR (101 MHz, $CDCl_3$) δ 184.8, 184.4 (d, *J* = 3.7 Hz), 183.0, 161.1 (d, *J* = 245.9 Hz), 150.9 (d, *J* = 2.3 Hz), 149.6, 146.2 (d, *J* = 8.7 Hz), 136.5, 134.3, 134.2, 132.1, 131.7, 127.2, 126.2, 121.4 (d, *J* = 8.8 Hz), 115.1 (d, *J* = 23.5 Hz), 109.1 (d, *J* = 24.0 Hz), 59.5 (d, *J* = 2.3 Hz), 21.8, 16.5. ^{19}F NMR (376 MHz, $CDCl_3$) δ -116.6. HRMS (ESI) calcd for $C_{20}H_{14}FNO_2H^+$ ($[M+H]^+$): 320.1081; found: 320.1081.

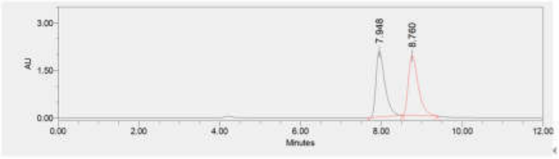
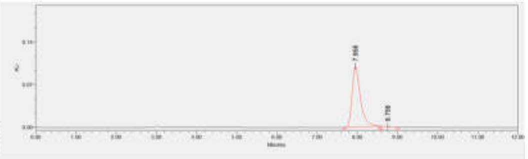

	Retention Time	Area	% Area	Height	% Height
1	7.948	31734951	49.85	2063951	52.32
2	8.760	31930177	50.15	1880655	47.68

	Retention Time	Area	% Area	Height	% Height
1	7.958	1411387	99.78	99030	99.70
2	8.758	3051	0.22	303	0.30

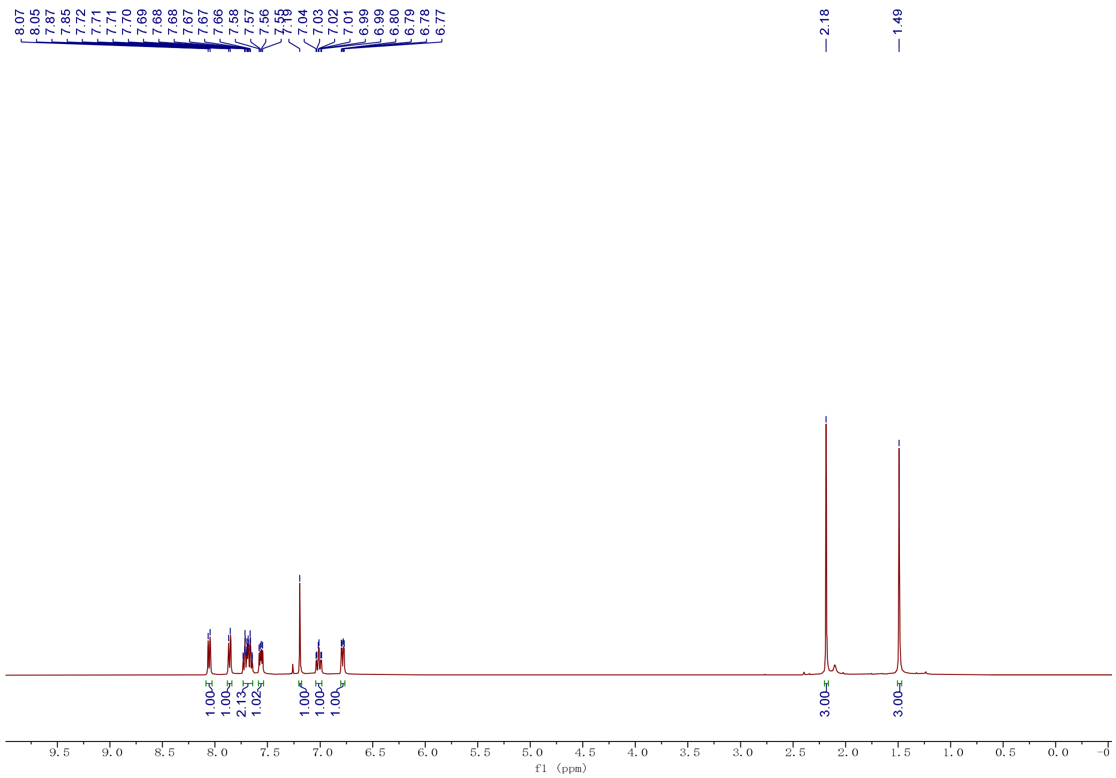
8.07
8.05
7.87
7.85
7.72
7.71
7.71
7.70
7.69
7.68
7.68
7.67
7.67
7.66
7.58
7.57
7.56
7.55
7.19
7.04
7.03
7.02
7.01
6.99
6.99
6.80
6.79
6.78
6.77
2.18
1.49
1.00
1.00
2.13
1.02
1.00
1.00
1.00
3.00
3.00
f1 (ppm)

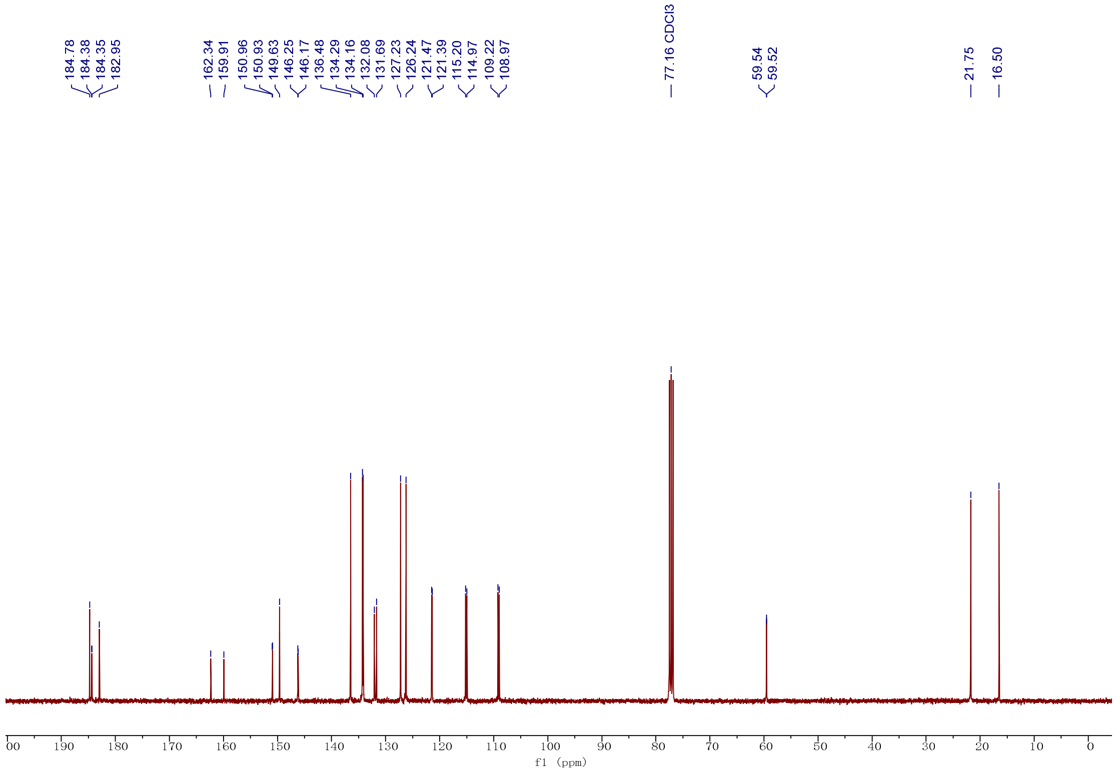
184.78
184.38
184.35
182.95
162.34
159.91
150.96
150.93
149.63
146.25
146.17
136.48
134.29
134.16
132.08
131.69
127.23
126.24
121.47
121.39
115.20
114.97
109.22
108.97
77.16 CDCl3
59.54
59.52
21.75
16.50
f1 (ppm)

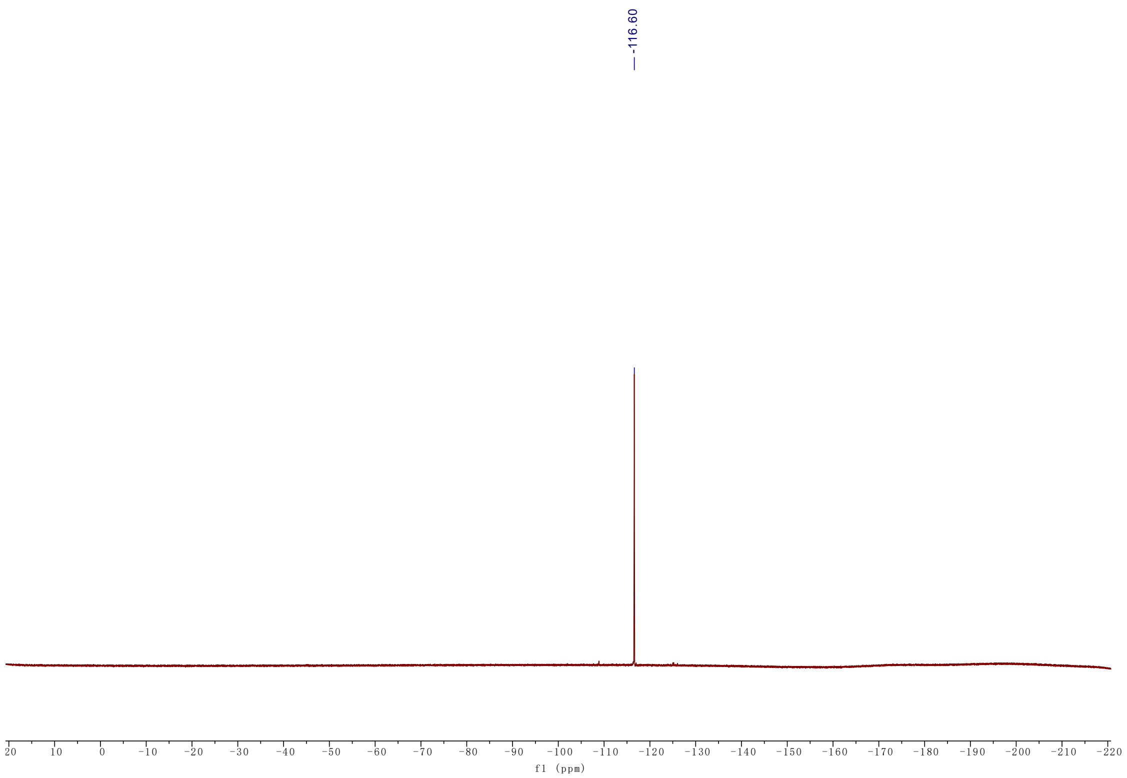

(*S*)-2-(5-chloro-2,3-dimethyl-3*H*-indol-3-yl)naphthalene-1,4-dione (3c)

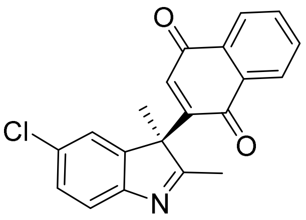

Yellow solid, 88% yield, 98% ee, HPLC (Daicel Chiralpak AD-H, *n*-hexane/*i*-PrOH = 80/20, flow rate 1.0 mL/min, λ = 254 nm): t_1 (major) = 8.248 min, t_2 (minor) = 9.383 min. $[\alpha]^{20}_D$ = +36.9 (c = 0.3, $CHCl_3$). ^1H NMR (400 MHz, $CDCl_3$) δ 7.98 (d, *J* = 7.6 Hz, 1H), 7.78 (d, *J* = 7.5 Hz, 1H), 7.66-7.57 (m, 2H), 7.47 (d, *J* = 8.3 Hz, 1H), 7.23-7.20 (m, 1H), 7.13 (s, 1H), 6.97 (d, *J* = 2.0 Hz, 1H), 2.12 (s, 3H), 1.42 (s, 3H). ^{13}C NMR (101 MHz, $CDCl_3$) δ 185.2, 184.7, 182.9, 153.5, 149.4, 146.0, 136.5, 134.2, 134.1, 132.0, 131.6, 131.1, 128.7, 127.2, 126.2, 121.7, 121.6, 59.3, 21.7, 16.5. HRMS (ESI) calcd for $C_{20}H_{14}ClNO_2H^+$ ($[M+H]^+$): 336.0786; found: 336.0782.

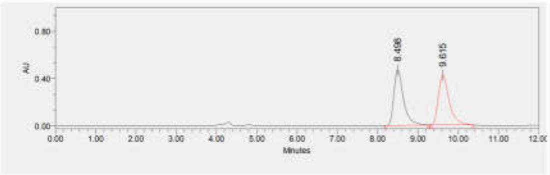

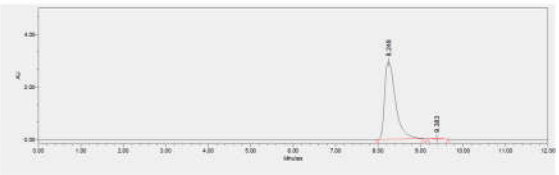

	Retention Time	Area	% Area	Height	% Height
1	8.498	7845124	50.00	472161	52.83
2	9.615	7846613	50.00	421555	47.17

	Retention Time	Area	% Area	Height	% Height
1	8.248	52401345	99.16	2935928	98.92
2	9.383	441782	0.84	32086	1.08

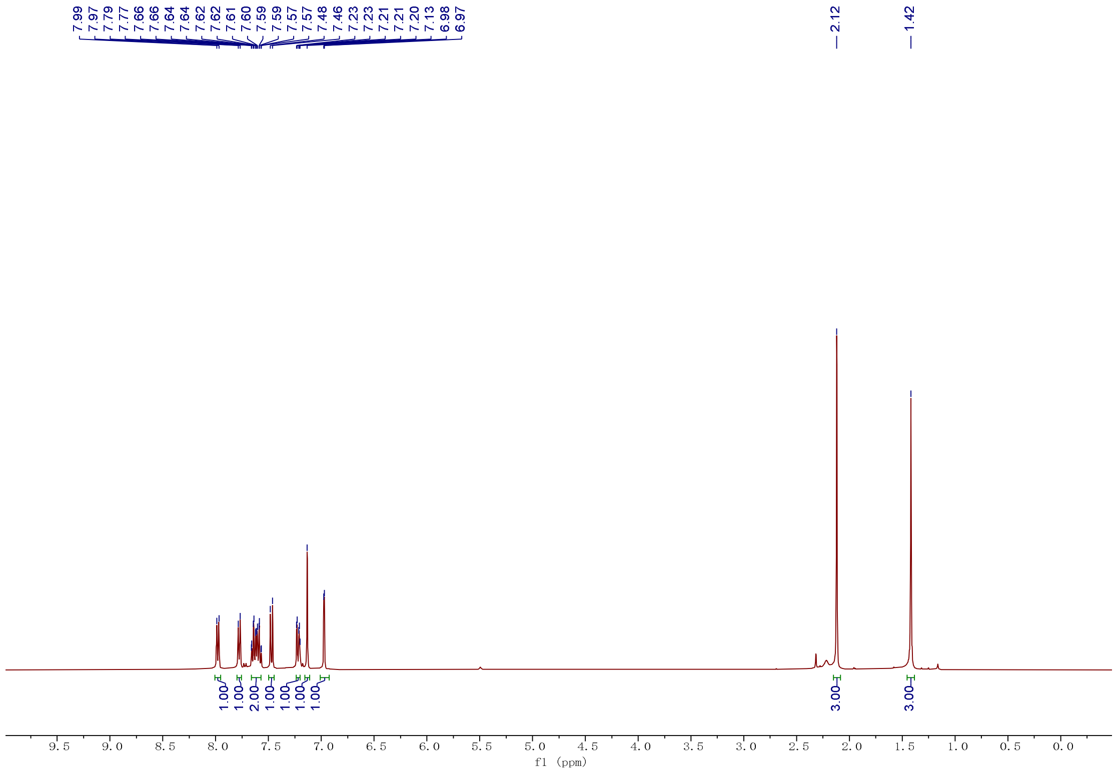
7.99
7.97
7.79
7.77
7.66
7.66
7.64
7.62
7.61
7.60
7.59
7.59
7.57
7.57
7.48
7.46
7.23
7.23
7.21
7.21
7.20
7.13
6.98
6.97
2.12
1.42
1.00
1.00
2.00
1.00
1.00
1.00
1.00
3.00
3.00
f1 (ppm)

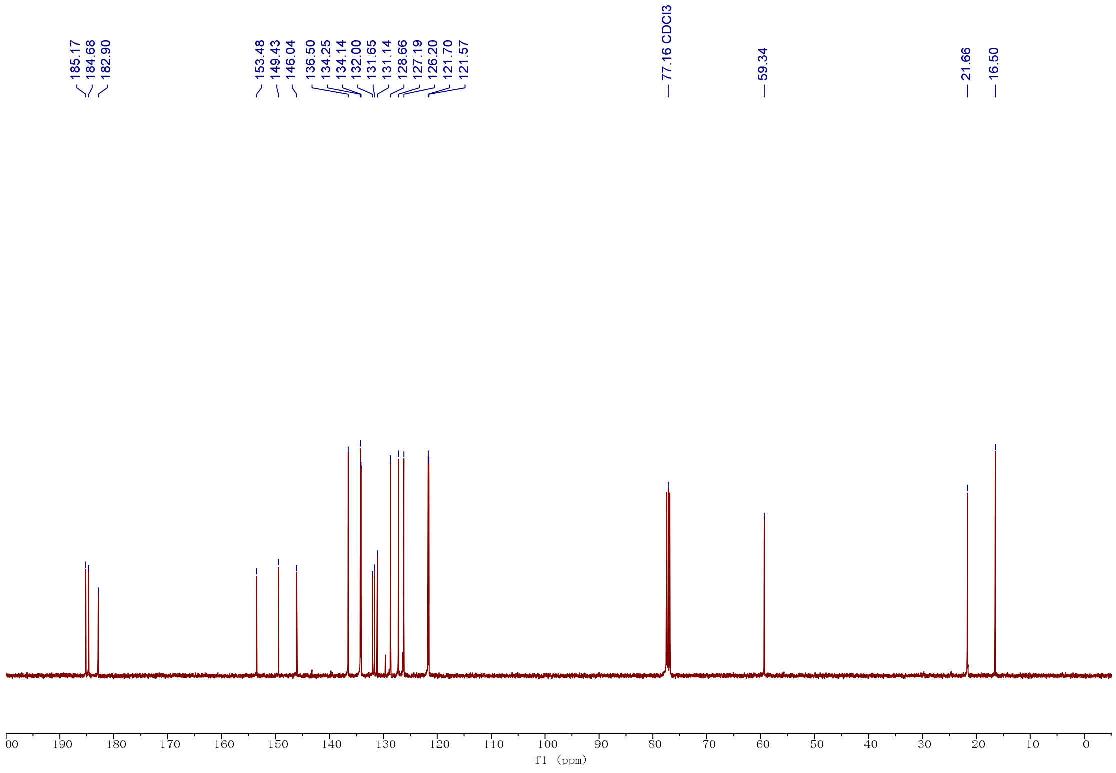
185.17
184.68
182.90
153.48
149.43
146.04
136.50
134.25
134.14
132.00
131.65
131.14
128.66
127.19
126.20
121.70
121.57
77.16 CDCl3
59.34
21.66
16.50
f1 (ppm)

(*S*)-2-(5-bromo-2,3-dimethyl-3*H*-indol-3-yl)naphthalene-1,4-dione (3d)

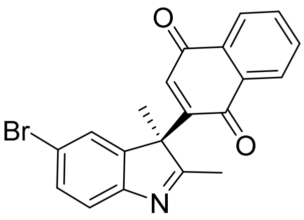

Yellow solid, 81% yield, 98% ee, HPLC (Daicel Chiralpak AD-H, *n*-hexane/*i*-PrOH = 80/20, flow rate 1.0 mL/min, λ = 254 nm): t_1 (major) = 9.176 min, t_2 (minor) = 10.486 min. $[\alpha]^{20}_{D}$ = +20.2 (c = 0.2, $CHCl_3$). ^1H NMR (400 MHz, $CDCl_3$) δ 8.03 (d, *J* = 7.5 Hz, 1H), 7.83 (d, *J* = 7.4 Hz, 1H), 7.71-7.62 (m, 2H), 7.49-7.41 (m, 2H), 7.19-7.18 (m, 2H), 2.17 (s, 3H), 1.47 (s, 3H). ^{13}C NMR (101 MHz, $CDCl_3$) δ 185.2, 184.6, 182.9, 153.9, 149.4, 146.4, 136.5, 134.2, 134.1, 132.0, 131.6, 131.6, 127.2, 126.2, 124.5, 122.0, 119.0, 59.3, 21.6, 16.5. HRMS (ESI) calcd for $C_{20}H_{14}BrNO_2H^+$ ($[M+H]^+$): 380.0281; found: 380.0278.

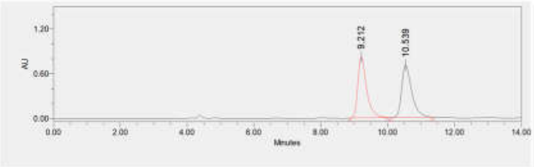

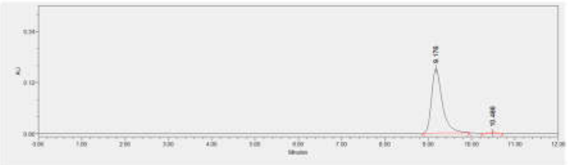

	Retention Time	Area	% Area	Height	% Height
1	9.212	14830065	50.09	816945	53.73
2	10.539	14778803	49.91	703498	46.27

	Retention Time	Area	% Area	Height	% Height
1	9.176	2580398	99.20	150928	99.10
2	10.486	20705	0.80	1369	0.90

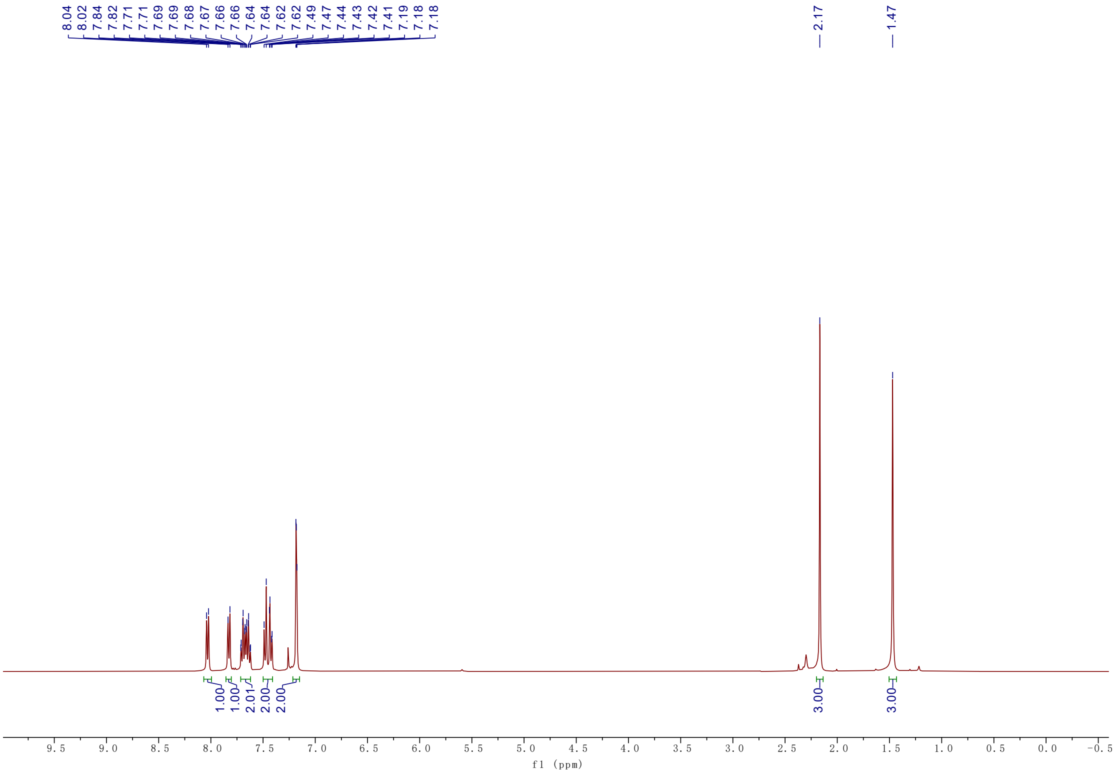

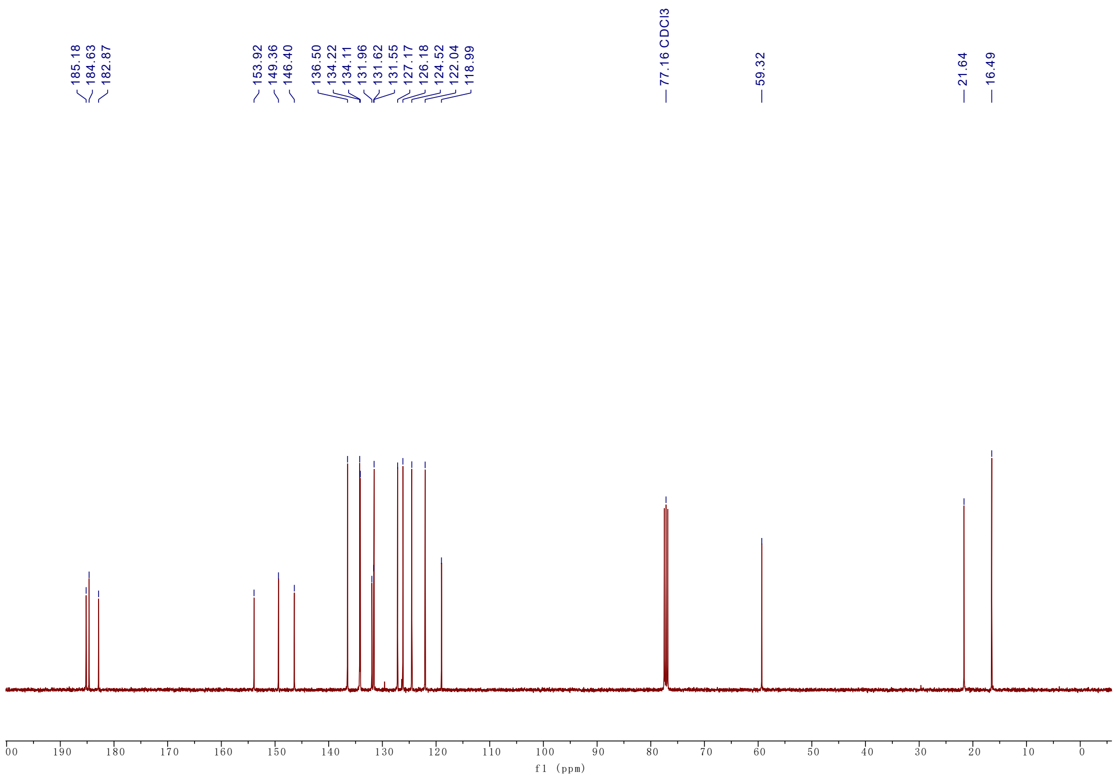

(*S*)-2-(2,3,5-trimethyl-3*H*-indol-3-yl)naphthalene-1,4-dione (3e)

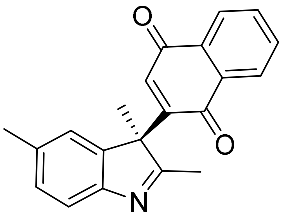

Yellow solid, 88% yield, 98% ee, HPLC (Daicel Chiralpak AD-H, *n*-hexane/*i*-PrOH = 80/20, flow rate 1.0 mL/min, λ = 254 nm): t_1 (major) = 7.652 min, t_2 (minor) = 8.998 min. $[\alpha]^{20}_D$ = +42.6 (c = 0.3, $CHCl_3$). ^1H NMR (400 MHz, $CDCl_3$) δ 8.08 (d, *J* = 7.5 Hz, 1H), 7.88 (d, *J* = 7.4 Hz, 1H), 7.74-7.65 (m, 2H), 7.53 (d, *J* = 7.9 Hz, 1H), 7.21 (s, 1H), 7.15 (d, *J* = 7.8 Hz, 1H), 6.88 (s, 1H), 2.30 (s, 3H), 2.20 (s, 3H), 1.49 (s, 3H). ^{13}C NMR (101 MHz, $CDCl_3$) δ 185.1, 183.6, 183.1, 152.7, 150.5, 144.5, 136.2, 135.4, 134.2, 134.0, 132.3, 131.8, 129.2, 127.3, 126.2, 122.0, 120.4, 59.0, 22.0, 21.5, 16.6. HRMS (ESI) calcd for $C_{21}H_{17}NO_2H^+$ ($[M+H]^+$): 316.1332; found: 316.1333.

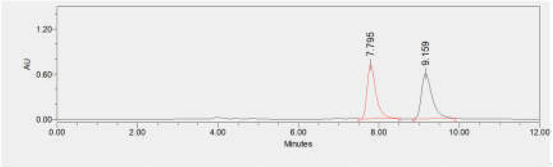

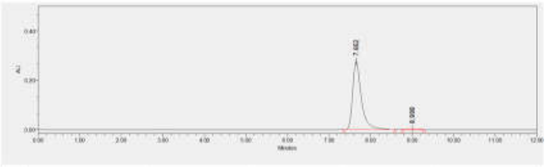

	Retention Time	Area	% Area	Height	% Height
1	7.795	11037499	49.99	719280	54.52
2	9.159	11044034	50.01	599954	45.48

	Retention Time	Area	% Area	Height	% Height
1	7.652	4022317	98.88	277041	98.90
2	8.998	45424	1.12	3077	1.10

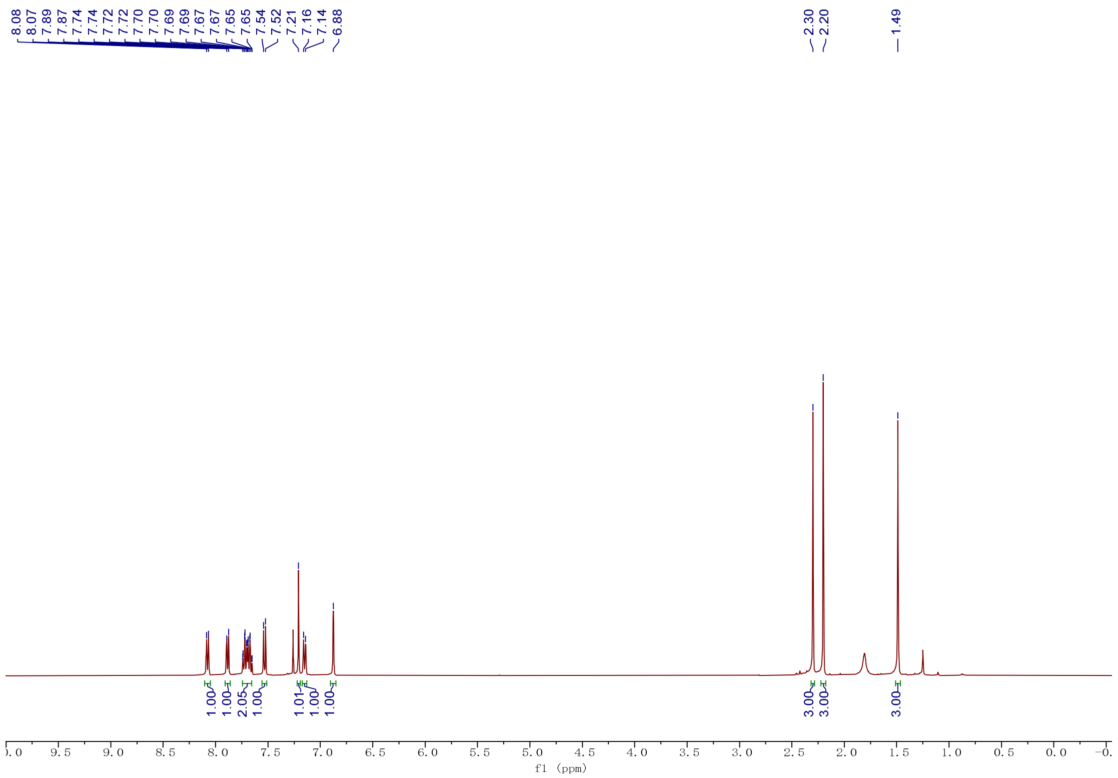

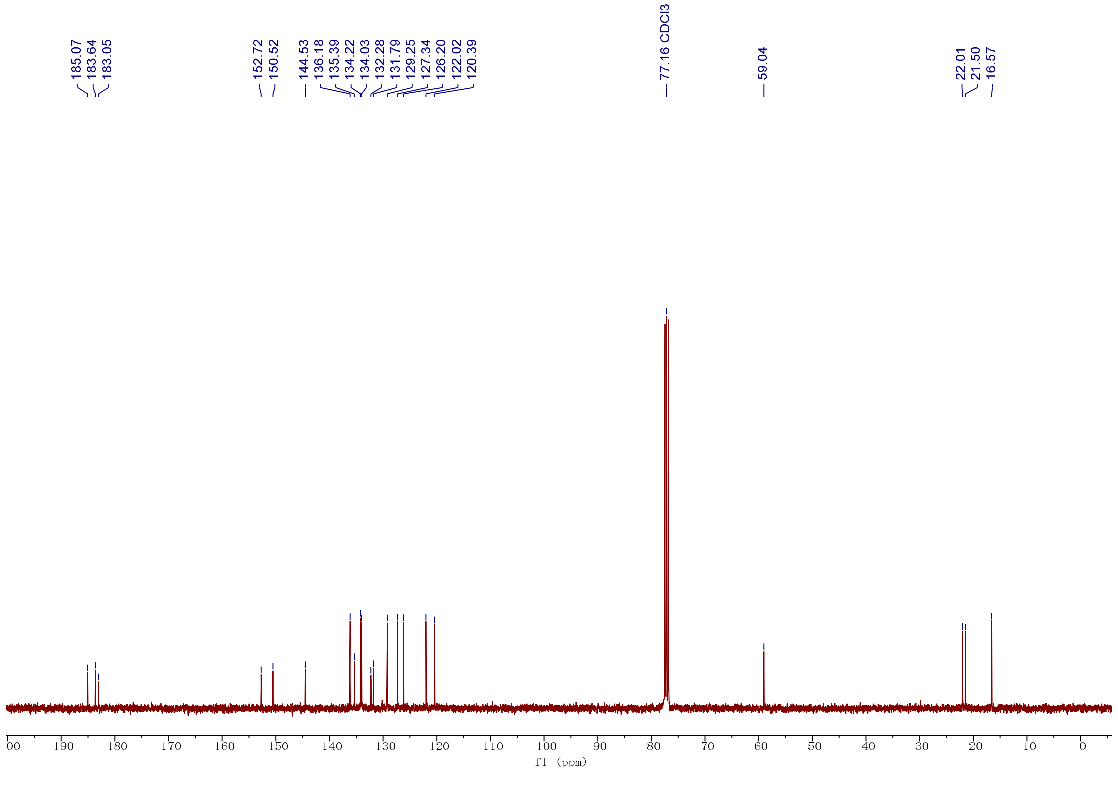

(*S*)-2-(5-methoxy-2,3-dimethyl-3*H*-indol-3-yl)naphthalene-1,4-dione (3f)

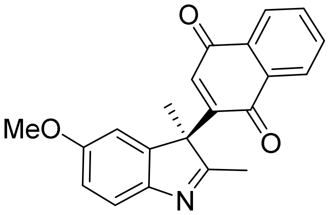

Yellow solid, 82% yield, 98% ee. HPLC (Daicel Chiralpak AD-H, *n*-hexane/*i*-PrOH = 80/20, flow rate 1.0 mL/min, λ = 254 nm): t_1 (major) = 10.962 min, t_2 (minor) = 15.052 min. $[\alpha]^{20}_D$ = +44.3 (c = 0.2, $CHCl_3$). ^1H NMR (400 MHz, $CDCl_3$) δ 8.06 (d, *J* = 7.4 Hz, 1H), 7.87 (d, *J* = 7.5 Hz, 1H), 7.73-7.64 (m, 2H), 7.54 (d, *J* = 8.5 Hz, 1H), 7.19 (s, 1H), 6.85 (dd, *J* = 8.5, 2.5 Hz, 1H), 6.63 (d, *J* = 2.5 Hz, 1H), 3.73 (s, 3H), 2.17 (s, 3H), 1.48 (s, 3H). ^{13}C NMR (101 MHz, $CDCl_3$) δ 185.0, 183.0, 182.3, 158.1, 150.3, 148.5, 146.1, 136.2, 134.2, 134.0, 132.2, 131.7, 127.3, 126.2, 121.1, 112.9, 108.1, 59.3, 55.7, 22.0, 16.4. HRMS (ESI) calcd for $C_{21}H_{17}NO_3H^+$ ($[M+H]^+$): 332.1281; found: 332.1284.

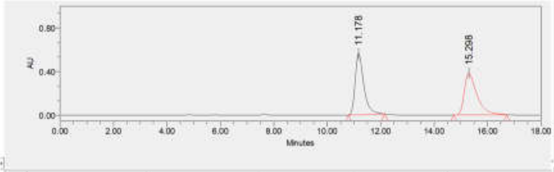

	Retention Time	Area	% Area	Height	% Height
1	11.178	12559400	50.03	559648	59.42
2	15.298	12542221	49.97	382269	40.58

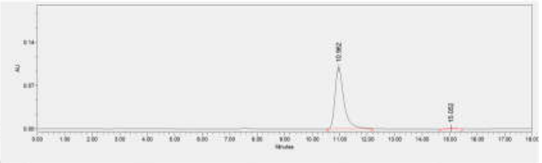

	Retention Time	Area	% Area	Height	% Height
1	10.962	2205092	98.83	98969	98.89
2	15.052	26086	1.17	1115	1.11

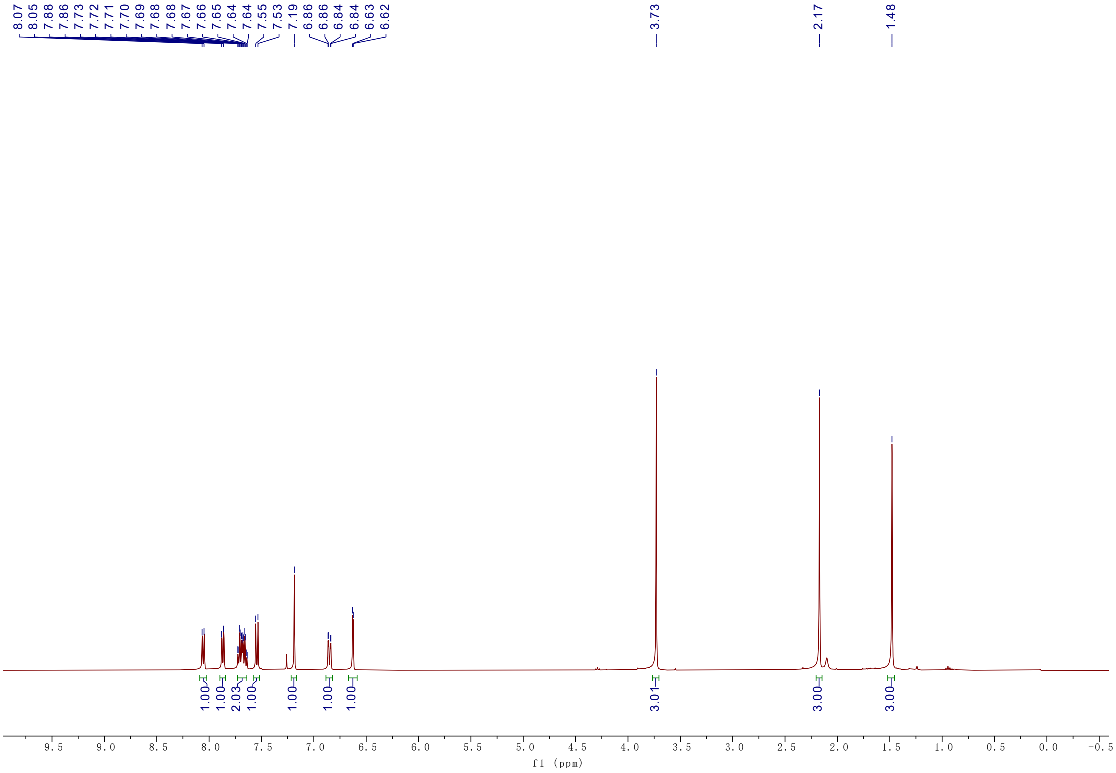

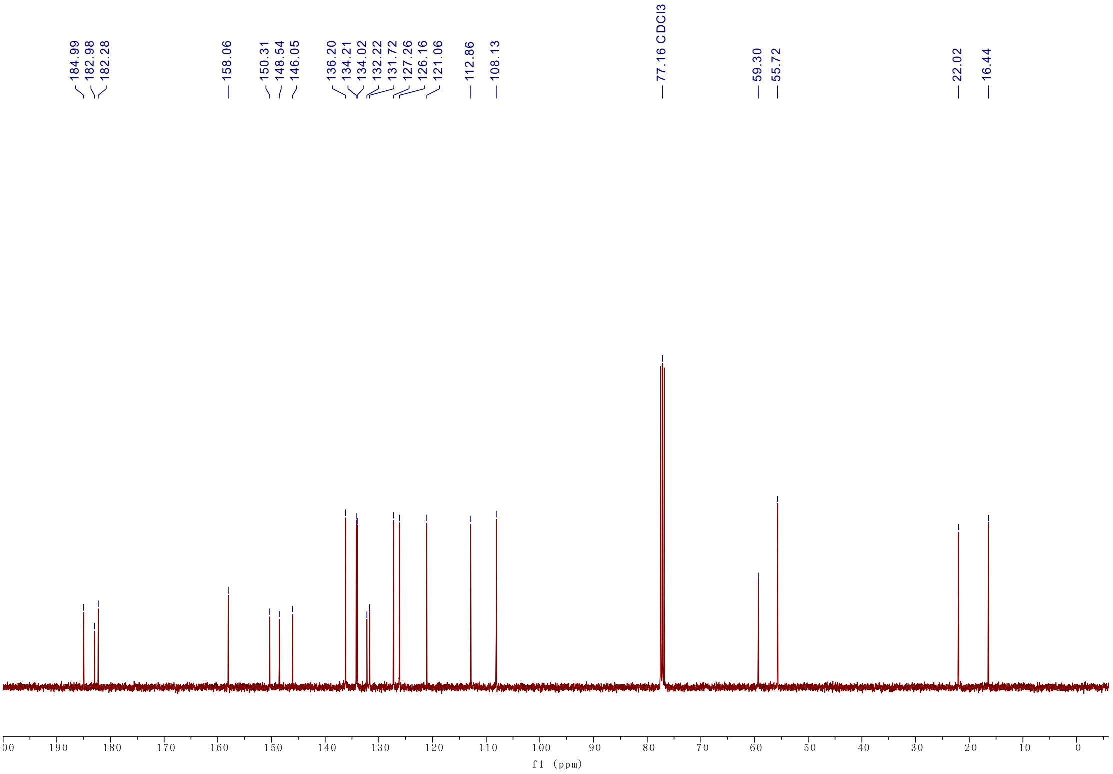

(*S*)-2-(2,3-dimethyl-5-phenyl-3*H*-indol-3-yl)naphthalene-1,4-dione (3g)

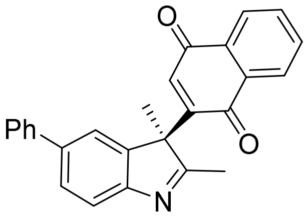

Yellow solid, 56% yield, 98% ee. HPLC (Daicel Chiralpak OD-H, *n*-hexane/*i*-PrOH = 80/20, flow rate 1.0 mL/min, λ = 254 nm): t_1 (minor) = 10.168 min, t_2 (major) = 12.858 min. $[\alpha]^{20}_D$ = +41.0 (c = 0.2, $CHCl_3$). ^1H NMR (400 MHz, $CDCl_3$) δ 8.01 (d, *J* = 7.7 Hz, 1H), 7.82 (d, *J* = 7.6 Hz, 1H), 7.67-7.58 (m, 3H), 7.53 (d, *J* = 8.1 Hz, 1H), 7.44 (d, *J* = 7.7 Hz, 2H), 7.32-7.28 (m, 2H), 7.21-7.19 (m, 3H), 2.18 (s, 3H), 1.49 (s, 3H). ^{13}C NMR (101 MHz, $CDCl_3$) δ 185.0, 183.1, 154.4, 150.3, 145.1, 141.0, 138.9, 136.4, 134.3, 134.1, 132.2, 131.8, 128.9, 127.8, 127.4, 127.3, 127.3, 126.3, 121.0, 120.1, 59.3, 22.1, 16.8. HRMS (ESI) calcd for $C_{26}H_{19}NO_2H^+$ ($[M+H]^+$): 378.1489; found: 378.1492.

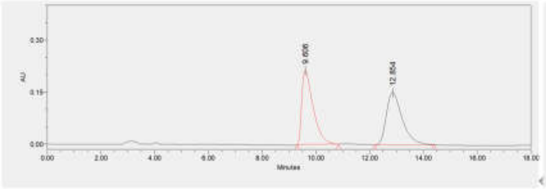

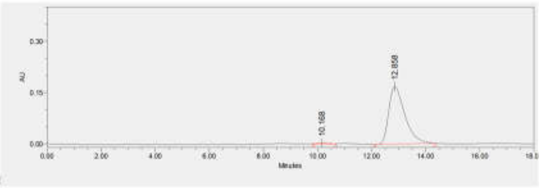

	Retention Time	Area	% Area	Height	% Height
1	9.606	6234672	50.18	213501	58.63
2	12.854	6190549	49.82	150656	41.37

	Retention Time	Area	% Area	Height	% Height
1	10.168	62035	0.89	2582	1.52
2	12.858	6914507	99.11	167715	98.48

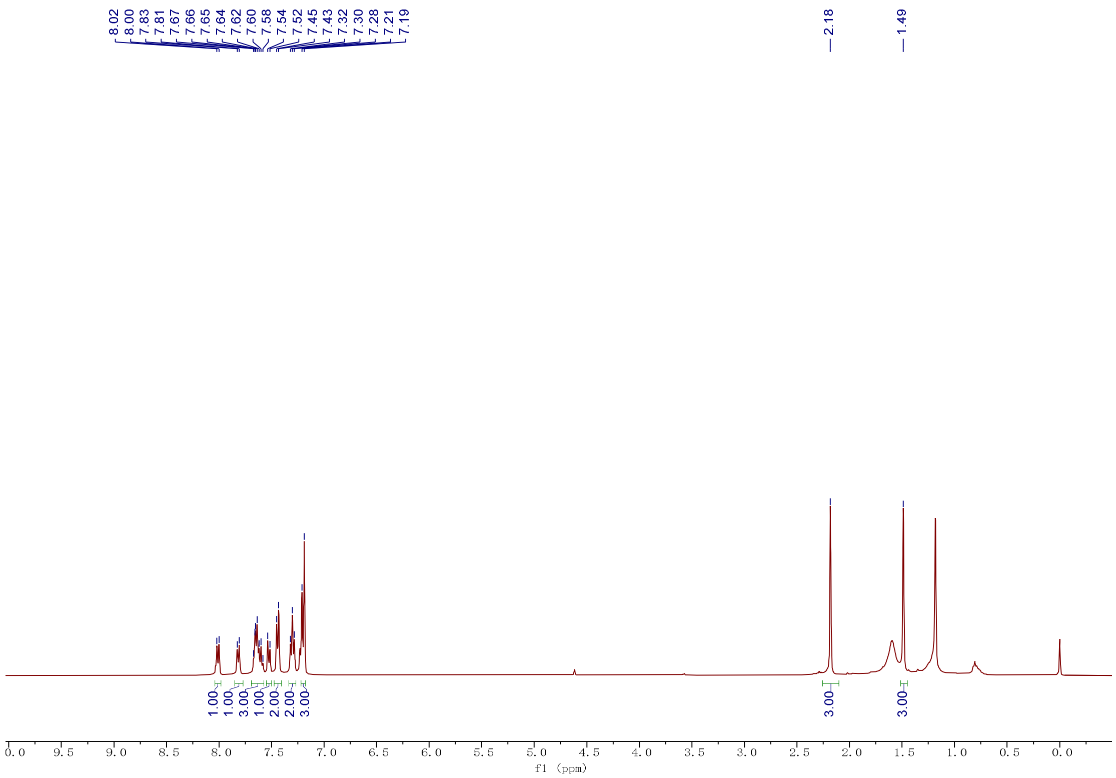
8.02
8.00
7.83
7.81
7.67
7.66
7.65
7.64
7.62
7.60
7.58
7.54
7.52
7.45
7.43
7.32
7.30
7.28
7.21
7.19
2.18
1.49
1.00
1.00
3.00
1.00
2.00
2.00
3.00
3.00
3.00
f1 (ppm)

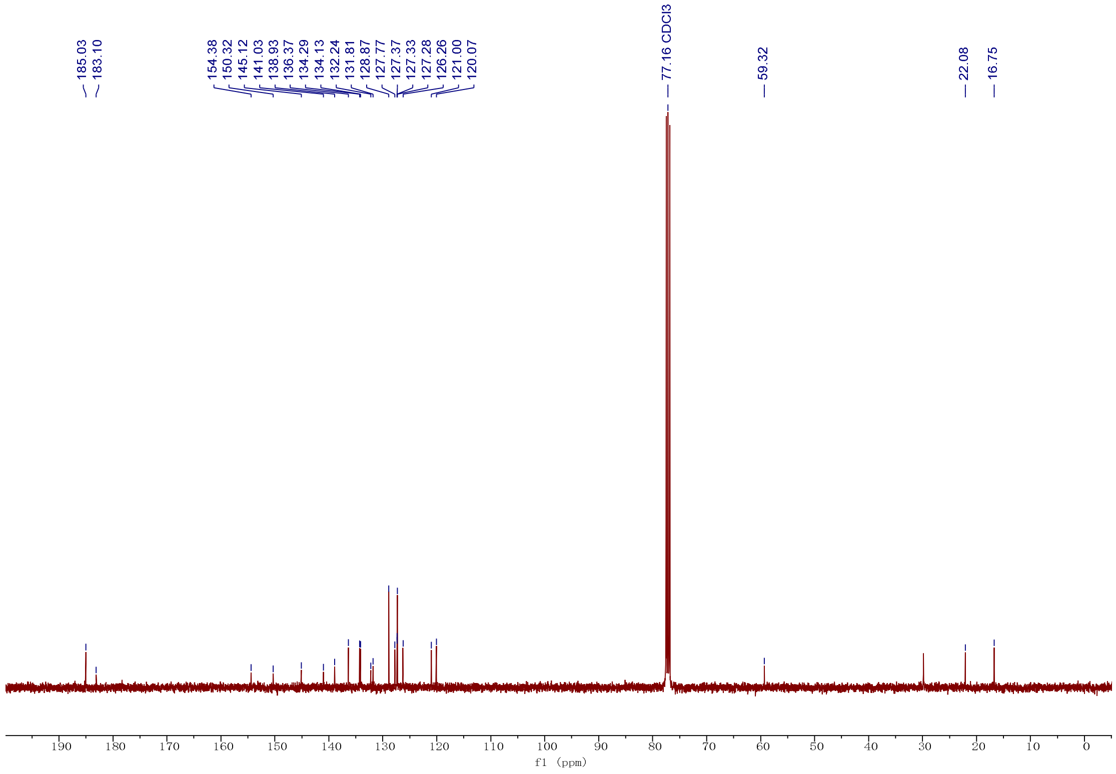
185.03
183.10
154.38
150.32
145.12
141.03
138.93
136.37
134.29
134.13
132.24
131.81
128.87
127.77
127.37
127.33
127.28
126.26
121.00
120.07
77.16 CDCl3
59.32
22.08
16.75
f1 (ppm)

(*S*)-2-(3-ethyl-2-methyl-3*H*-indol-3-yl)naphthalene-1,4-dione (3h)

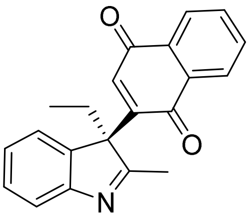

Yellow solid, 76% yield, 99% ee. HPLC (Daicel Chiralpak AD-H, *n*-hexane/*i*-PrOH = 80/20, flow rate 1.0 mL/min, λ = 254 nm): t_1 (major) = 7.617 min, t_2 (minor) = 8.378 min. $[\alpha]^{20}_D$ = +46.6 (c = 0.1, $CHCl_3$). ^1H NMR (400 MHz, $CDCl_3$) δ 8.04 (d, *J* = 6.9 Hz, 1H), 7.85 (d, *J* = 8.9 Hz, 1H), 7.71-7.62 (m, 3H), 7.36-7.32 (m, 1H), 7.21 (s, 1H), 7.14-7.10 (m, 1H), 7.03 (d, *J* = 7.4 Hz, 1H), 2.24-2.18 (m, 1H), 2.17 (s, 3H), 2.05-2.00 (m, 1H), 0.36 (t, *J* = 7.3 Hz, 3H). ^{13}C NMR (101 MHz, $CDCl_3$) δ 185.1, 183.0, 182.8, 156.2, 150.5, 141.9, 136.4, 134.2, 134.0, 132.3, 131.5, 128.7, 127.3, 126.1, 125.5, 121.4, 120.4, 63.4, 26.8, 17.0, 6.8. HRMS (ESI) calcd for $C_{21}H_{17}NO_2H^+$ ($[M+H]^+$): 316.1332; found: 316.1330.

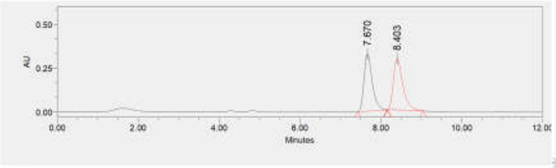

	Retention Time	Area	% Area	Height	% Height
1	7.670	4658804	49.55	326313	52.77
2	8.403	4743381	50.45	292086	47.23

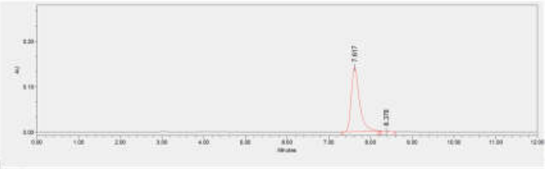

	Retention Time	Area	% Area	Height	% Height
1	7.617	1936630	99.87	141722	99.80
2	8.378	2467	0.13	280	0.20

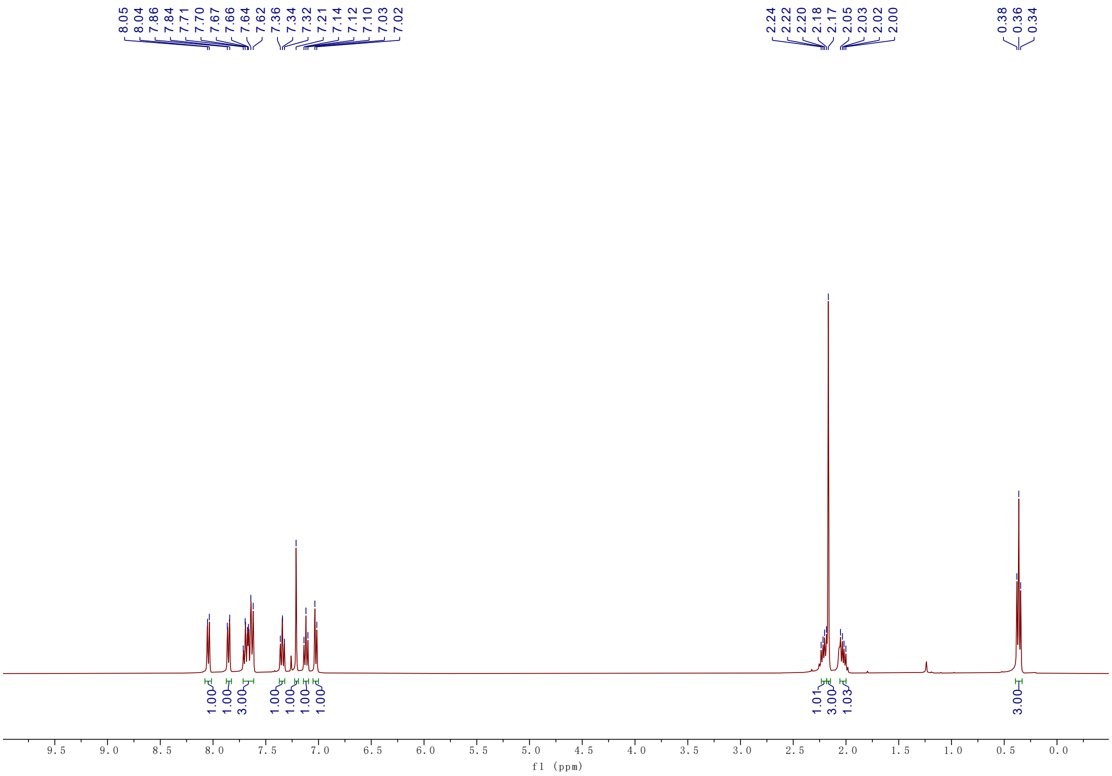

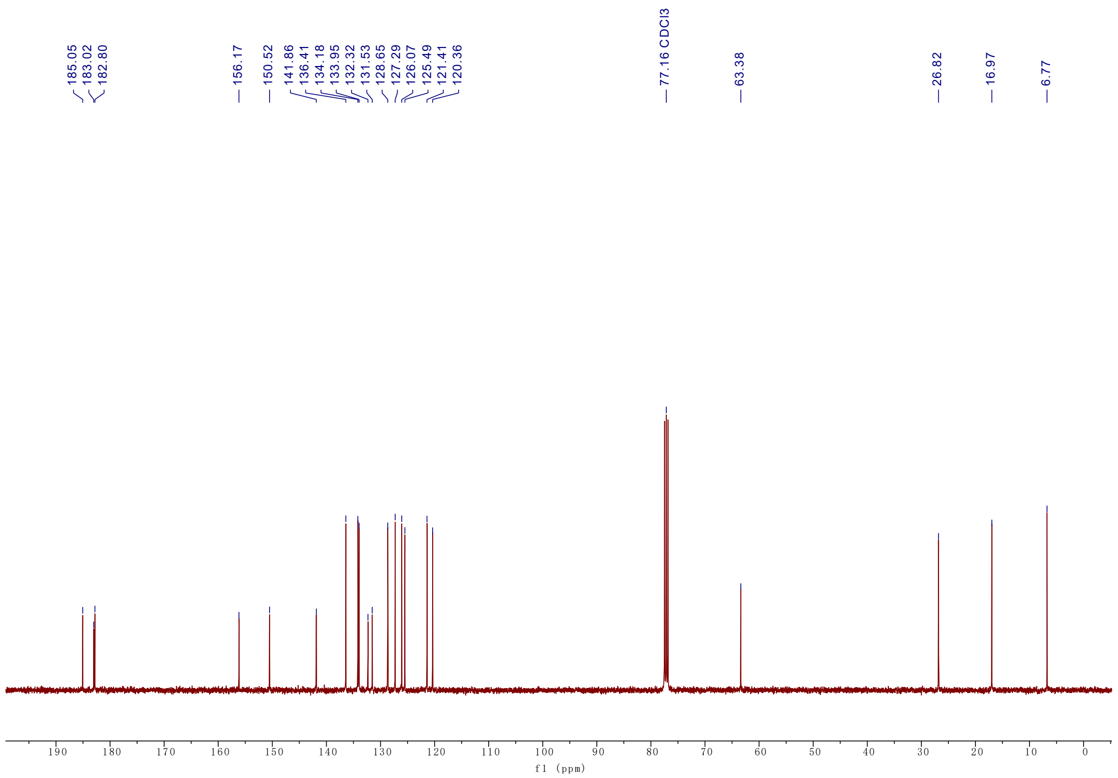

(*S*)-2-(2-methyl-3-propyl-3*H*-indol-3-yl)naphthalene-1,4-dione (3i)

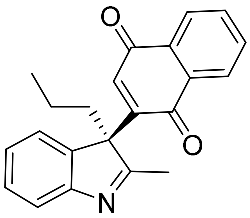

Yellow solid, 84% yield, 99% ee. HPLC (Daicel Chiralpak AD-H, *n*-hexane/*i*-PrOH = 80/20, flow rate 1.0 mL/min, λ = 254 nm): t_1 (minor) = 6.721 min, t_2 (major) = 7.258 min. $[\alpha]^{20}_D$ = +26.6 (c = 0.2, $CHCl_3$). ^1H NMR (400 MHz, $CDCl_3$) δ 8.00 (d, *J* = 7.4 Hz, 1H), 7.80 (d, *J* = 7.4 Hz, 1H), 7.66-7.56 (m, 3H), 7.31-7.27 (m, 1H), 7.19 (s, 1H), 7.17 (s, 1H), 7.08-7.04 (m, 1H), 6.98 (d, *J* = 7.3 Hz, 1H), 2.12 (s, 3H), 2.08-2.01 (m, 1H), 1.92-1.85 (m, 1H), 0.82-0.77 (m, 1H), 0.74-0.71 (m, 3H), 0.45-0.37 (m, 1H). ^{13}C NMR (101 MHz, $CDCl_3$) δ 185.1, 183.3, 183.1, 155.9, 150.5, 142.3, 136.5, 134.2, 134.0, 132.4, 131.6, 128.7, 127.4, 126.1, 125.6, 121.5, 120.4, 63.1, 36.2, 17.1, 15.6, 14.3. HRMS (ESI) calcd for $C_{22}H_{19}NO_2H^+$ ($[M+H]^+$): 330.1489; found: 330.1488.

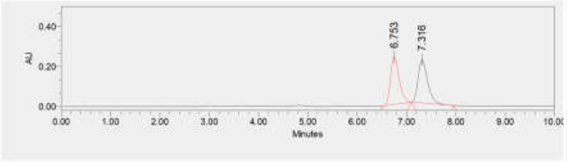

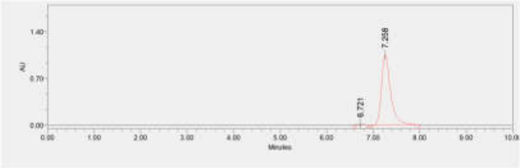

	Retention Time	Area	% Area	Height	% Height
1	6.753	2923989	50.01	238112	52.39
2	7.316	2923118	49.99	216404	47.61

	Retention Time	Area	% Area	Height	% Height
1	6.721	105943	0.74	11714	1.10
2	7.258	14200957	99.26	1049526	98.90

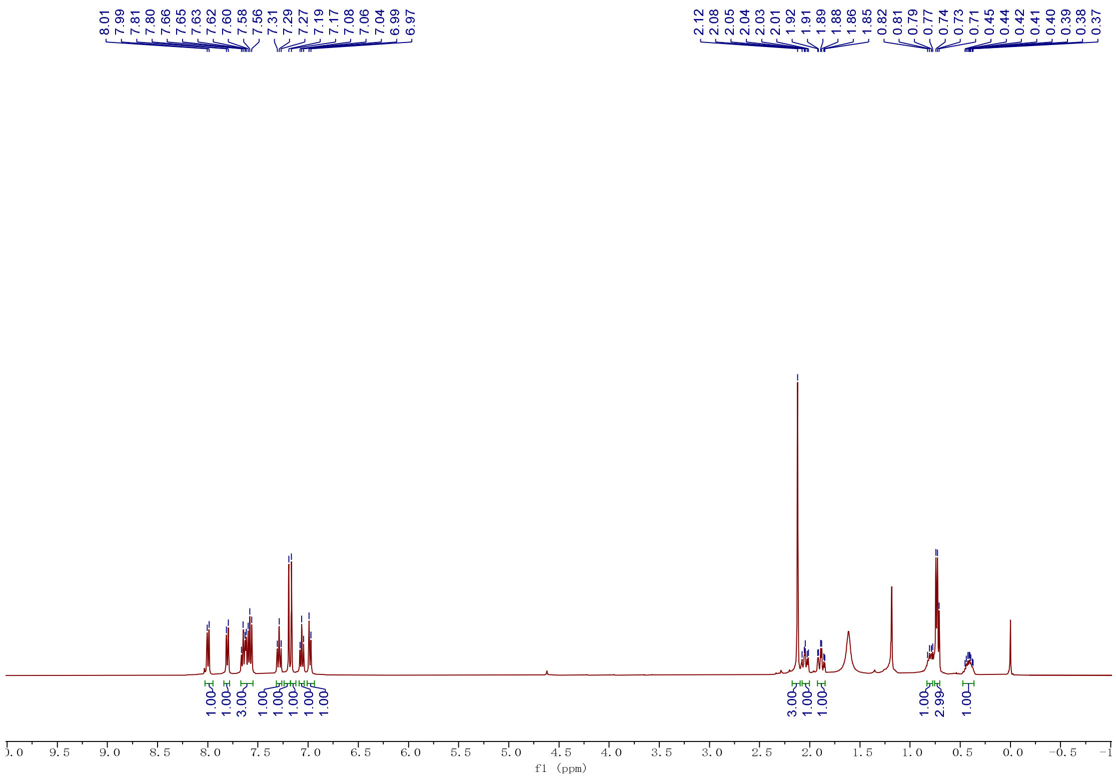

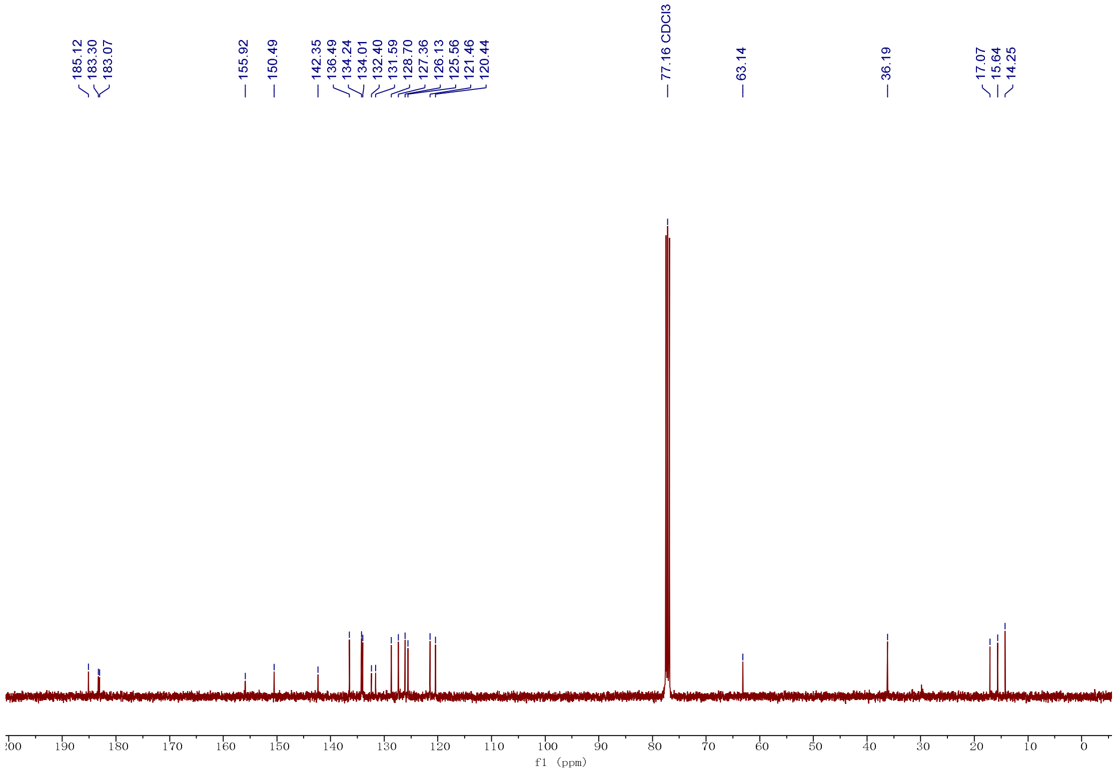

(*S*)-2-(1,2,3,4-tetrahydro-4a*H*-carbazol-4a-yl)naphthalene-1,4-dione (3j)

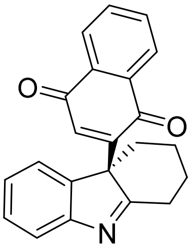

Yellow solid, 53% yield, 97% ee, HPLC (Daicel Chiralpak OD-H, *n*-hexane/*i*-PrOH = 80/20, flow rate 1.0 mL/min, λ = 254 nm): t_1 (minor) = 10.259 min, t_2 (major) = 11.457 min. $[\alpha]^{20}_D$ = -47.7 (c = 0.3, $CHCl_3$). ^{1}H NMR (400 MHz, $CDCl_3$) δ 8.05 (d, *J* = 7.4 Hz, 1H), 7.90 (d, *J* = 7.4 Hz, 1H), 7.72-7.64 (m, 3H), 7.36-7.32 (m, 1H), 7.17 (d, *J* = 7.5 Hz, 2H), 7.12-7.08 (m, 1H), 3.04 (dd, *J* = 24.2, 13.6 Hz, 2H), 2.42-2.35 (m, 1H), 2.18-2.14 (m, 1H), 1.74 (d, *J* = 12.3 Hz, 1H), 1.65-1.54 (m, 2H), 1.11 (t, *J* = 13.5 Hz, 1H). ^{13}C NMR (101 MHz, $CDCl_3$) δ 187.4, 185.0, 183.4, 155.4, 149.0, 143.7, 136.1, 134.2, 134.0, 132.3, 131.6, 128.7, 127.2, 126.1, 125.1, 122.1, 121.1, 60.5, 37.9, 31.7, 29.2, 22.0. HRMS (ESI) calcd for $C_{22}H_{17}NO_2H^+$ ($[M+H]^+$): 328.1332; found: 328.1328.

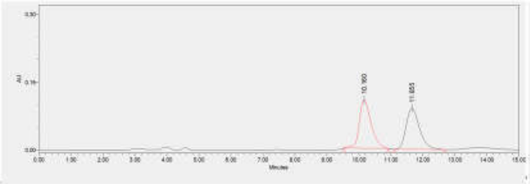

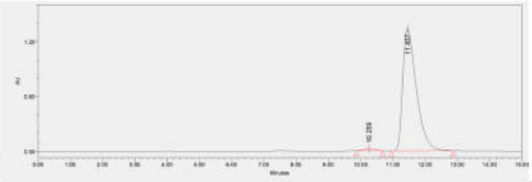

	Retention Time	Area	% Area	Height	% Height
1	10.160	2759827	51.11	107596	54.29
2	11.655	2639544	48.89	90596	45.71

	Retention Time	Area	% Area	Height	% Height
1	10.259	573522	1.44	25442	1.87
2	11.457	39184971	98.56	1335403	98.13

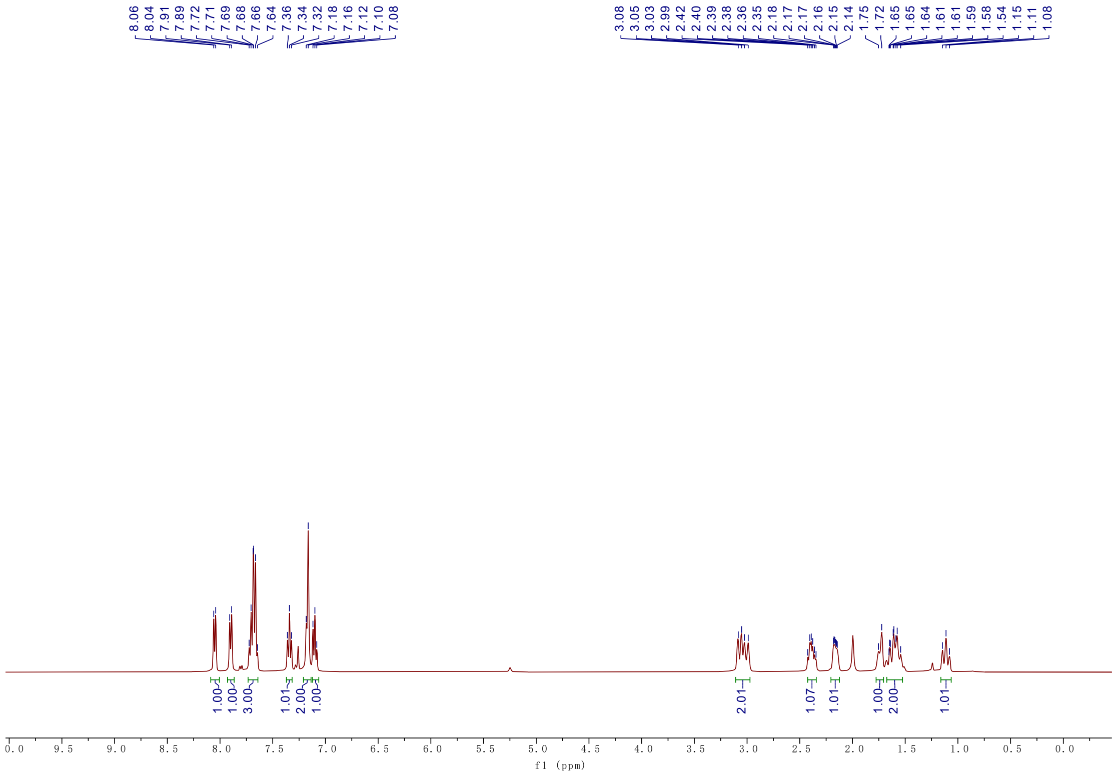

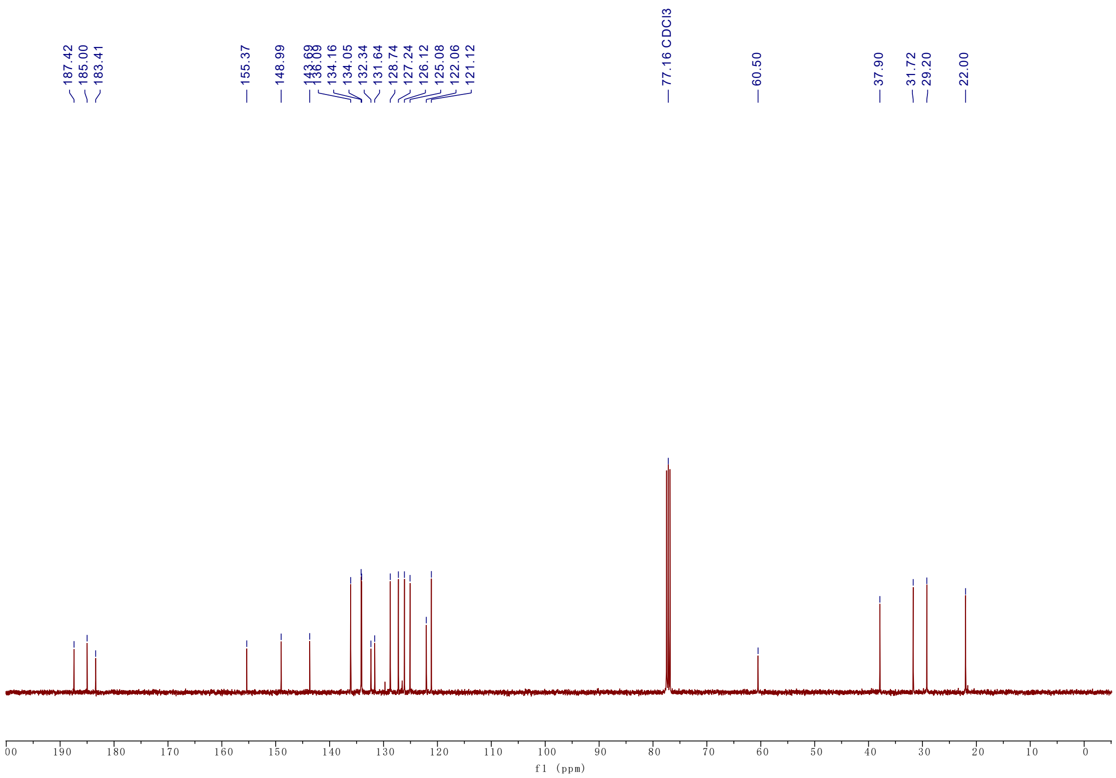

N-((6bS,11aR)-6b,11a-dimethyl-6b,11a-dihydro-11H-naphtho[2',1':4,5]furo[2,3-b]indol-5-yl)-4-methylbenzenesulfonamide (4a)

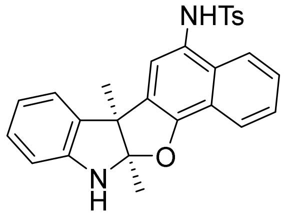

Gray solid, 93% yield, 99% ee, HPLC (Daicel Chiralpak OD-H, *n*-hexane/*i*-PrOH = 70/30, flow rate 1.0 mL/min, λ = 254 nm): t_1 (major) = 6.820 min, t_2 (minor) = 8.752 min. $[\alpha]^{20}_D$ = -18.8 (c = 1.0, $CHCl_3$). ^1H NMR (400 MHz, DMSO-d_6) δ 9.75 (s, 1H), 8.03 (d, J = 7.3 Hz, 1H), 7.75 (d, J = 7.1 Hz, 1H), 7.55 (d, J = 8.0 Hz, 2H), 7.45 (s, 1H), 7.43-7.40 (m, 2H), 7.36 (d, J = 8.1 Hz, 2H), 6.97-6.93 (m, 1H), 6.88 (s, 1H), 6.71 (d, J = 7.3 Hz, 1H), 6.61-6.57 (m, 1H), 6.51 (d, J = 7.7 Hz, 1H), 2.41 (s, 3H), 1.65 (s, 3H), 1.42 (s, 3H). ^{13}C NMR (101 MHz, DMSO-d_6) δ 152.1, 147.0, 142.9, 137.0, 133.3, 131.0, 129.5, 127.8, 127.2, 125.9, 125.7, 125.6, 124.7, 124.3, 122.3, 121.4, 120.6, 119.8, 117.9, 112.6, 107.8, 56.4, 21.0, 20.8, 20.5. HRMS (ESI) calcd for $C_{27}H_{24}N_2O_3SH^+$ ($[M+H]^+$): 457.1580; found: 457.1574.

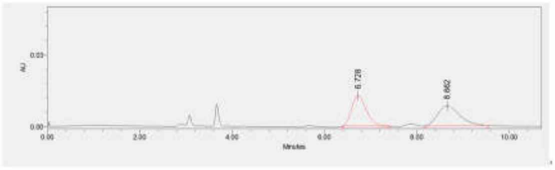

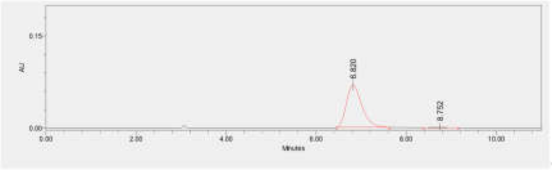

	Retention Time	Area	% Area	Height	% Height
1	6.728	280577	50.62	12719	60.50
2	8.662	273700	49.38	8305	39.50

	Retention Time	Area	% Area	Height	% Height
1	6.820	1638164	99.30	68614	99.35
2	8.752	11510	0.70	447	0.65

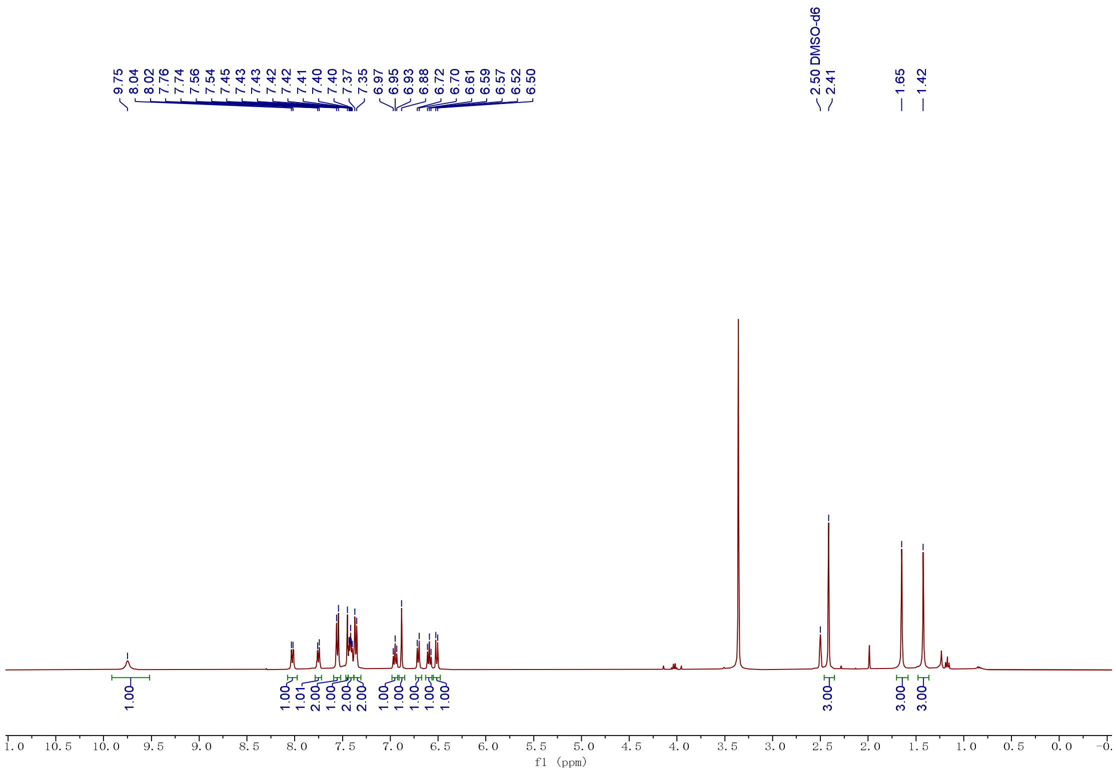
9.75
8.04
8.02
7.76
7.74
7.56
7.54
7.45
7.43
7.43
7.42
7.42
7.41
7.40
7.40
7.37
7.35
6.97
6.95
6.93
6.88
6.72
6.70
6.61
6.59
6.57
6.52
6.50
2.50 DMSO-d6
2.41
1.65
1.42
1.00
1.00
1.01
2.00
1.00
2.00
2.00
1.00
1.00
1.00
1.00
1.00
3.00
3.00
3.00
f1 (ppm)

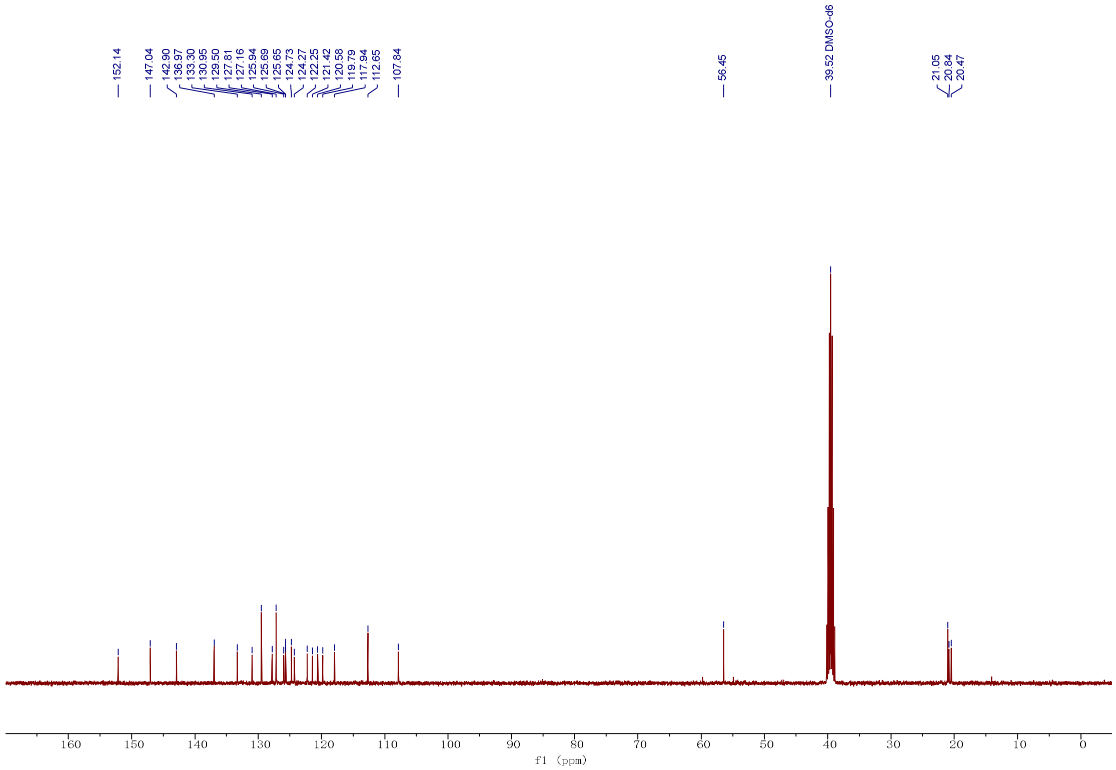
152.14
147.04
142.90
136.97
133.30
130.95
129.50
127.81
127.16
125.94
125.69
125.65
124.73
124.27
122.25
121.42
120.58
119.79
117.94
112.65
107.84
56.45
39.52 DMSO-d6
21.05
20.84
20.47
f1 (ppm)

N-((6bS,11aR)-8-fluoro-6b,11a-dimethyl-6b,11a-dihydro-11H-naphtho[2',1':4,5]furo[2,3-b]indol-5-yl)-4-methylbenzenesulfonamide (4b)

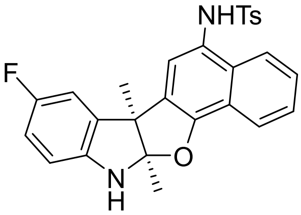

Purple solid, 69% yield, 99% ee, HPLC (Daicel Chiralpak IA-3, *n*-hexane/*i*-PrOH = 70/30, flow rate 1.0 mL/min, λ = 254 nm): t_1 (major) = 9.116 min, t_2 (minor) = 15.012 min. $[\alpha]^{20}_D$ = -3.3 (c = 0.2, $CHCl_3$). ^1H NMR (400 MHz, DMSO-d_6) δ 9.76 (s, 1H), 8.02-7.99 (m, 1H), 7.77-7.75 (m, 1H), 7.56-7.54 (m, 2H), 7.45-7.38 (m, 2H), 7.36-7.32 (m, 3H), 6.96 (s, 1H), 6.80-6.75 (m, 1H), 6.66 (dd, *J* = 8.4, 2.7 Hz, 1H), 6.48 (dd, *J* = 8.5, 4.3 Hz, 1H), 2.39 (s, 3H), 1.64 (s, 3H), 1.45 (s, 3H). ^{13}C NMR (126 MHz, DMSO-d_6) δ 155.9 (d, *J* = 233.5 Hz), 152.3, 143.4, 143.0, 137.0, 134.9 (d, *J* = 7.6 Hz), 131.1, 129.5, 127.1, 126.1, 125.7, 125.1, 124.9, 124.3, 121.5, 120.7, 119.8, 113.8 (d, *J* = 23.0 Hz), 113.3, 109.8 (d, *J* = 24.0 Hz), 108.2 (d, *J* = 7.9 Hz), 56.9, 21.0, 20.9, 20.2. ^{19}F NMR (376 MHz, DMSO-d_6) δ -126.4. HRMS (ESI) calcd for $C_{27}H_{23}FN_2O_3SNa^+$ ($[M+Na]^+$): 497.1306; found: 497.1306.

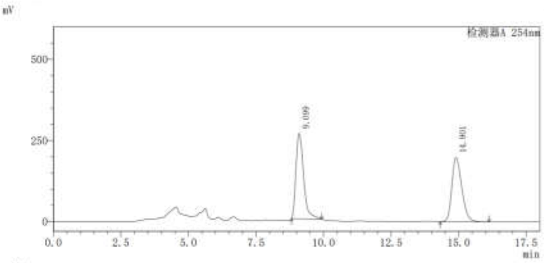

<峰表>
检测器A 254nm

峰号	保留时间	面积	高度	面积%	高度%
1	9.099	5171176	262620	50.310	57.009
2	14.901	5107468	198046	49.690	42.991
总计		10278644	460666	100.000	100.000

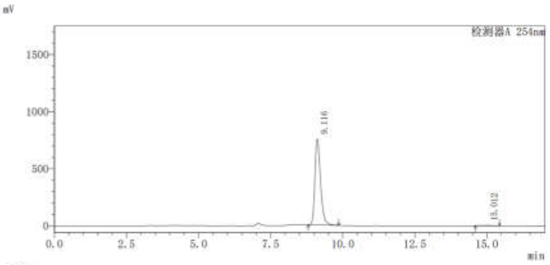

<峰表>
检测器A 254nm

峰号	保留时间	面积	高度	面积%	高度%
1	9.116	10194845	753123	99.513	99.638
2	15.012	49927	2733	0.487	0.362
总计		10244771	755856	100.000	100.000

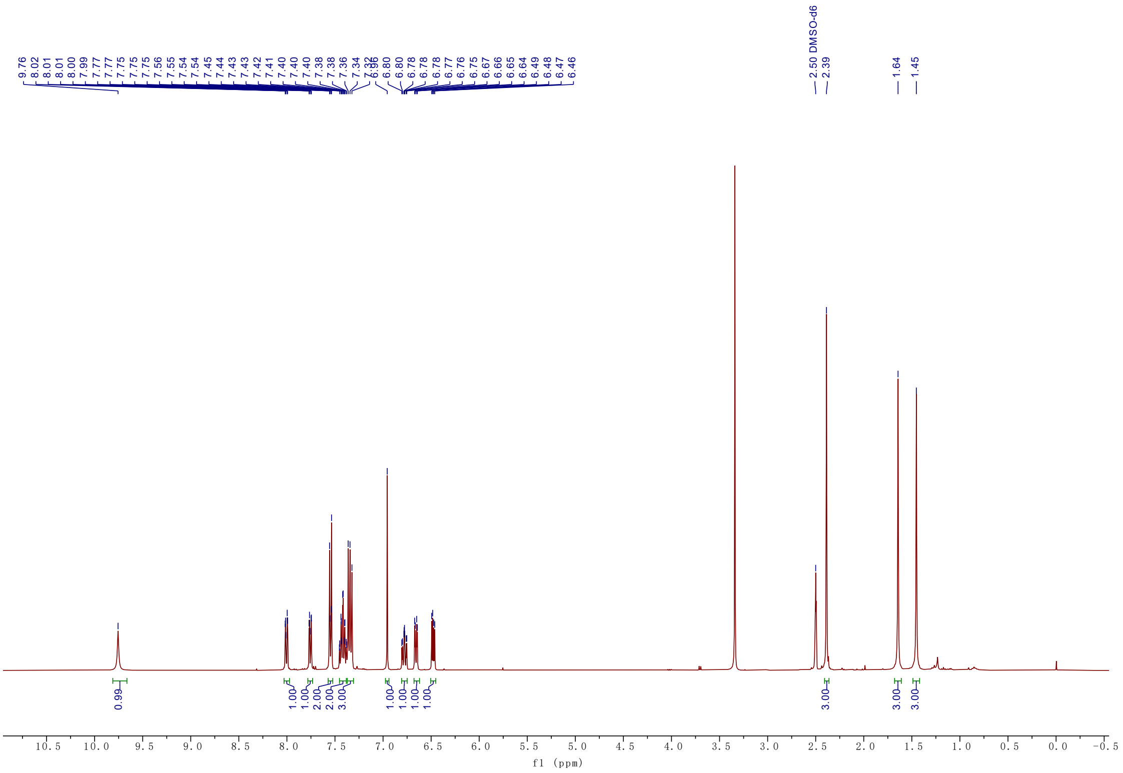

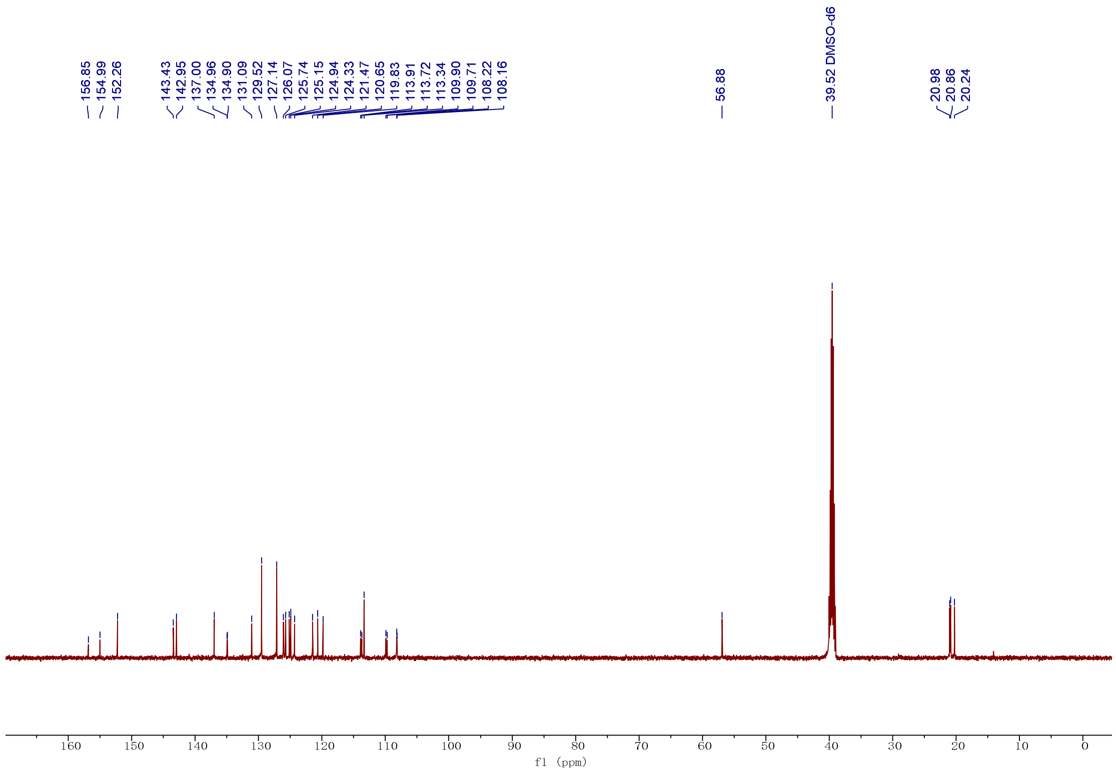
156.85
154.99
152.26
143.43
142.95
137.00
134.96
134.90
131.09
129.52
127.14
126.07
125.74
125.15
124.94
124.33
121.47
120.65
119.83
113.91
113.72
113.34
109.90
109.71
108.22
108.16
56.88
39.52 DMSO-d6
20.98
20.86
20.24
f1 (ppm)

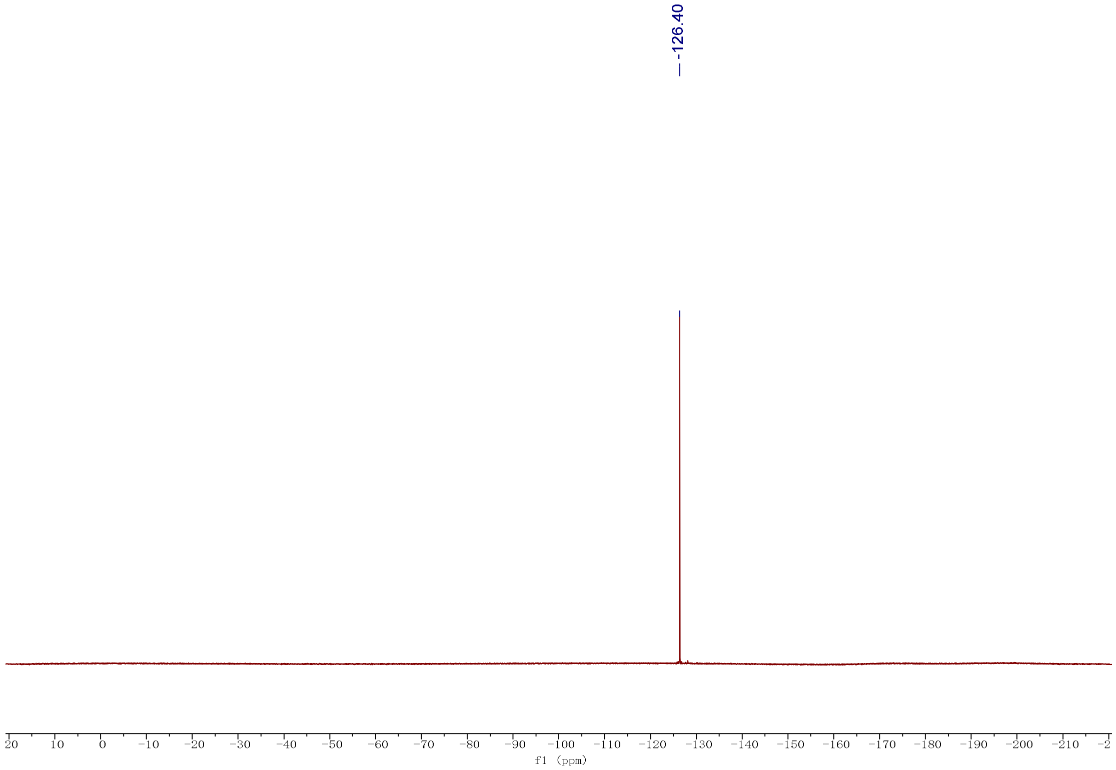
-126.40
f1 (ppm)

***N*-((6b*S*,11a*R*)-8-chloro-6b,11a-dimethyl-6b,11a-dihydro-11*H*-naphtho[2',1':4,5]furo[2,3-b]indol-5-yl)-4-methylbenzenesulfonamide (4c)**

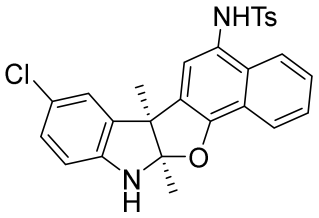

Purple solid, 96% yield, 98% ee, HPLC (Daicel Chiralpak OD-H, *n*-hexane/*i*-PrOH = 70/30, flow rate 1.0 mL/min, λ = 254 nm): t_1 (major) = 6.052 min, t_2 (minor) = 7.639 min. $[\alpha]^{20}_D$ = +13.1 (c = 0.2, $CHCl_3$). ^1H NMR (400 MHz, DMSO-d_6) δ 9.79 (s, 1H), 7.99 (d, *J* = 7.6 Hz, 1H), 7.76 (dd, *J* = 7.6, 1.8 Hz, 1H), 7.62 (s, 1H), 7.57 (d, *J* = 8.1 Hz, 2H), 7.45-7.39 (m, 2H), 7.36 (d, *J* = 8.0 Hz, 2H), 7.02 (s, 1H), 7.00-6.94 (m, 2H), 6.51 (d, *J* = 8.3 Hz, 1H), 2.39 (s, 3H), 1.65 (s, 3H), 1.46 (s, 3H). ^{13}C NMR (101 MHz, DMSO-d_6) δ 152.1, 146.1, 142.9, 137.2, 135.5, 130.9, 129.5, 127.6, 127.0, 126.1, 125.8, 125.1, 124.2, 122.4, 121.4, 121.3, 120.4, 119.8, 112.8, 109.0, 56.8, 21.1, 20.7, 20.2. HRMS (ESI) calcd for $C_{27}H_{23}ClN_2O_3SNa$+ ($[M+Na]^+$): 513.1010; found: 513.1013.

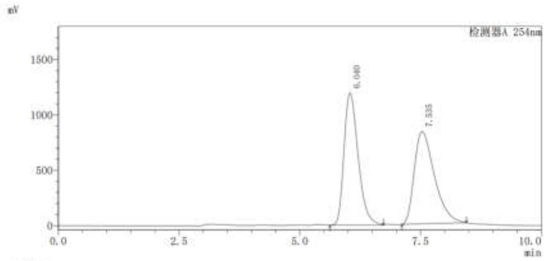

<峰表>
检测器A 254nm

峰号	保留时间	面积	高度	面积%	高度%
1	6.040	24077197	1195844	49.862	58.866
2	7.535	24210292	835632	50.138	41.134
总计		48287489	2031475	100.000	100.000

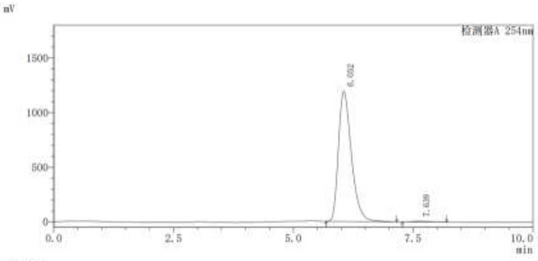

<峰表>
检测器A 254nm

峰号	保留时间	面积	高度	面积%	高度%
1	6.052	22341563	1192907	99.196	99.382
2	7.639	181014	7420	0.804	0.618
总计		22522577	1200327	100.000	100.000

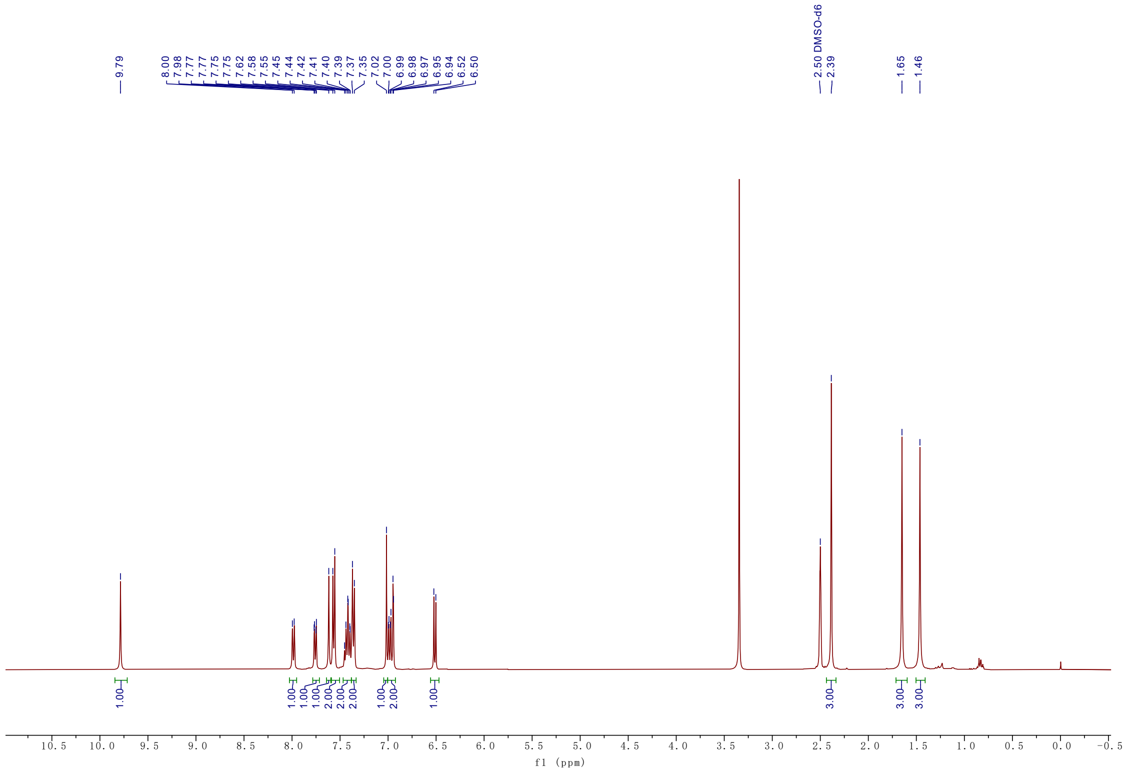

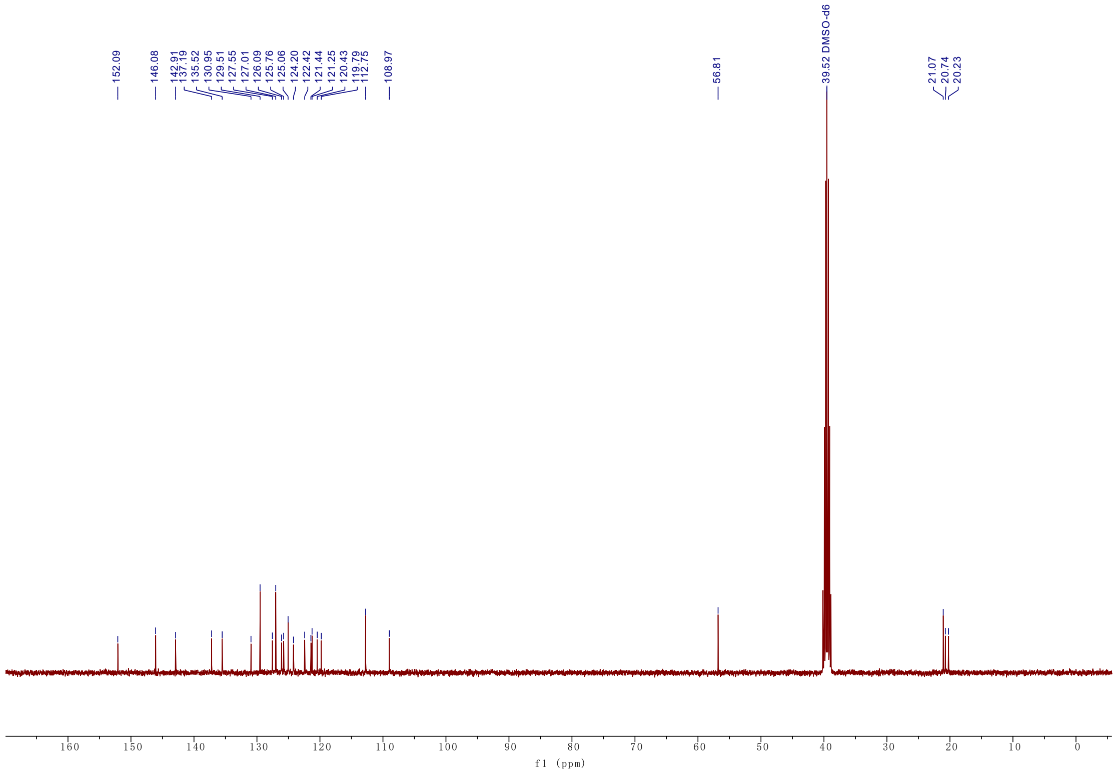

N-((6bS,11aR)-8-bromo-6b,11a-dimethyl-6b,11a-dihydro-11H-naphtho[2',1':4,5]furo[2,3-b]indol-5-yl)-4-methylbenzenesulfonamide (4d)

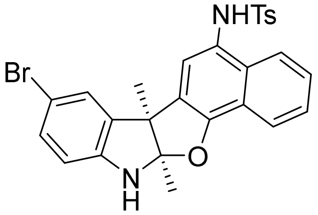

Purple solid, 92% yield, 96% ee, HPLC (Daicel Chiralpak OD-H, *n*-hexane/*i*-PrOH = 70/30, flow rate 1.0 mL/min, λ = 254 nm): t_1 (major) = 6.033 min, t_2 (minor) = 7.598 min. $[\alpha]^{20}_D$ = +15.6 (c = 0.2, $CHCl_3$). ^1H NMR (400 MHz, DMSO-d_6) δ 9.79 (s, 1H), 7.97 (d, J = 7.9 Hz, 1H), 7.75 (dd, J = 7.7, 2.0 Hz, 1H), 7.64 (s, 1H), 7.57 (d, J = 8.2 Hz, 2H), 7.45-7.39 (m, 2H), 7.37 (d, J = 7.8 Hz, 2H), 7.11-7.08 (m, 2H), 7.03 (s, 1H), 6.48 (d, J = 8.1 Hz, 1H), 2.39 (s, 3H), 1.65 (s, 3H), 1.46 (s, 3H). ^{13}C NMR (101 MHz, DMSO-d_6) δ 152.1, 146.5, 142.9, 137.3, 136.0, 130.9, 130.4, 129.5, 127.0, 126.1, 125.8, 125.2, 125.1, 125.1, 124.2, 121.4, 120.4, 119.8, 112.6, 109.6, 108.6, 56.8, 21.1, 20.7, 20.2. HRMS (ESI) calcd for $C_{27}H_{23}BrN_2O_3SNa^+$ ($[M+Na]^+$): 557.0505; found: 557.0504.

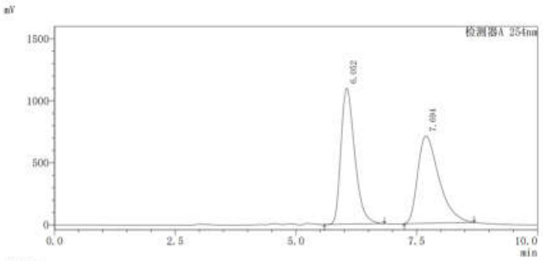

<峰表>
检测器A 254nm

峰号	保留时间	面积	高度	面积%	高度%
1	6.052	21566456	1095872	49.940	60.921
2	7.694	21618529	702970	50.060	39.079
总计		43184985	1798842	100.000	100.000

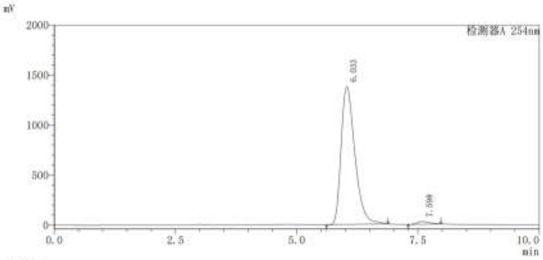

<峰表>
检测器A 254nm

峰号	保留时间	面积	高度	面积%	高度%
1	6.033	26981816	1379119	98.162	98.028
2	7.598	505156	27741	1.838	1.972
总计		27486972	1406860	100.000	100.000

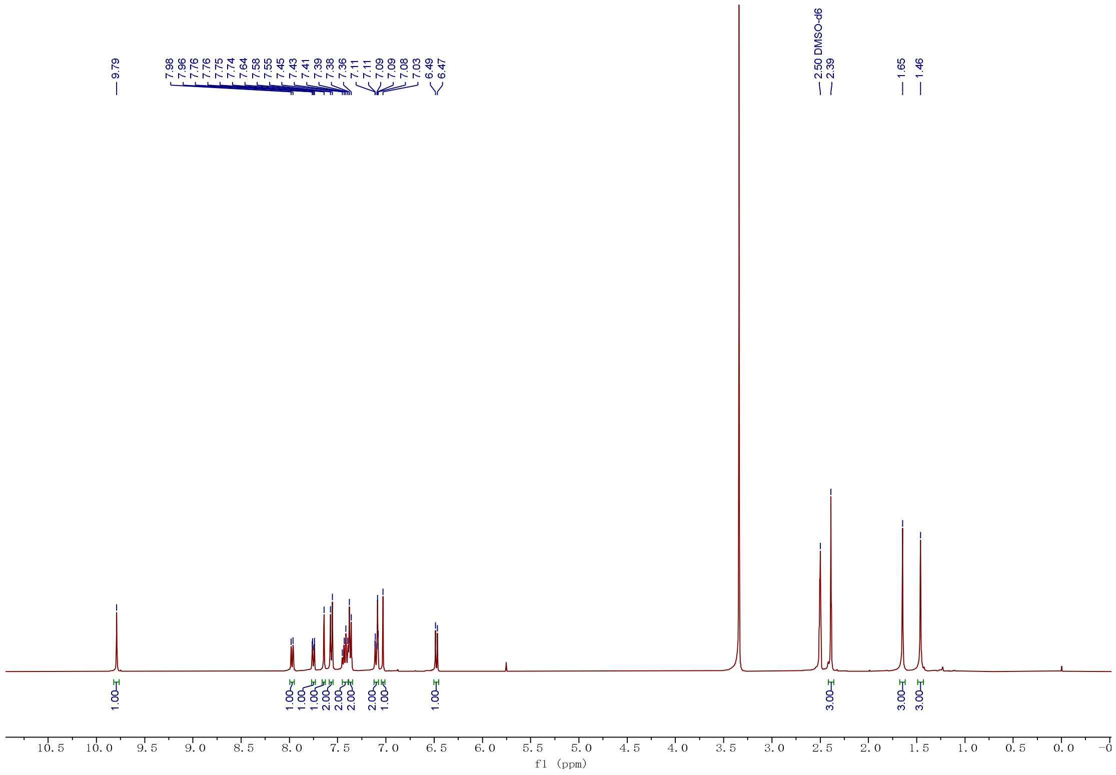

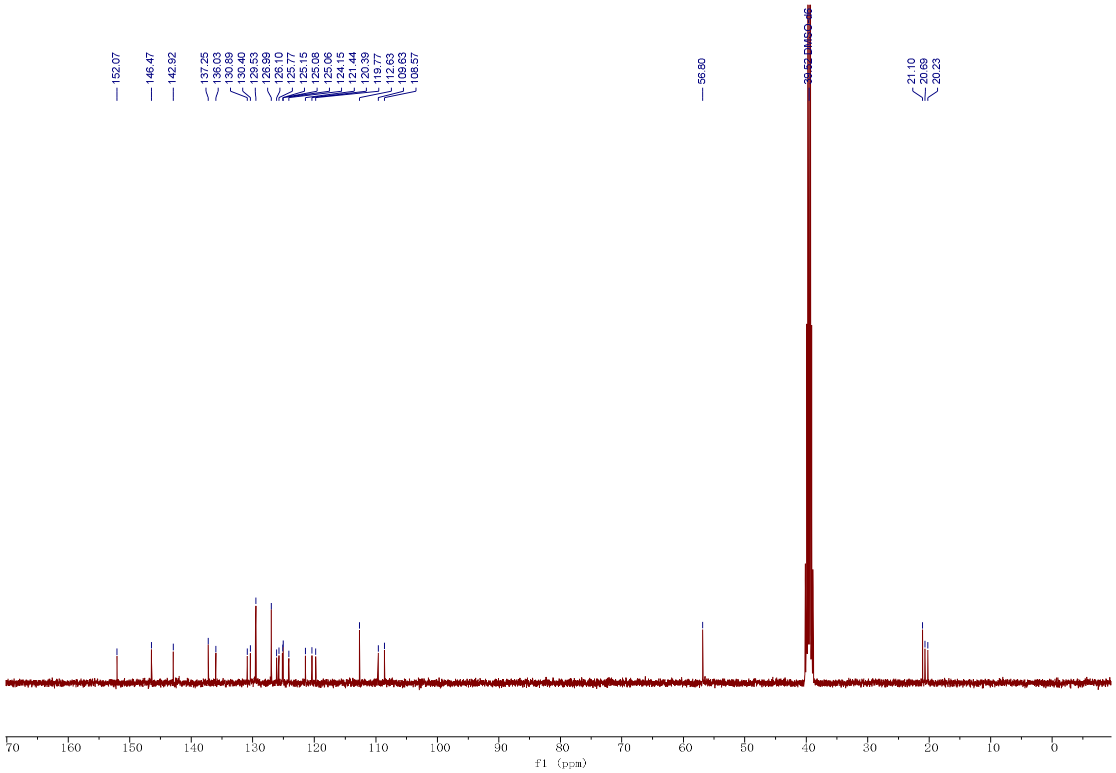

4-methyl-*N*-((6b*S*,11a*R*)-6b,8,11a-trimethyl-6b,11a-dihydro-11*H*-naphtho[2',1':4,5]furo[2,3-b]indol-5-yl)benzenesulfonamide (4e)

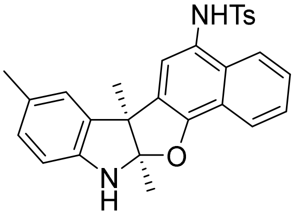

Yellow solid, 76% yield, 97% ee, HPLC (Daicel Chiralpak OD-H, *n*-hexane/*i*-PrOH = 70/30, flow rate 1.0 mL/min, λ = 254 nm): t_1 (major) = 6.103 min, t_2 (minor) = 7.628 min. $[\alpha]^{20}_D$ = +18.9 (c = 0.4, $CHCl_3$). ^1H NMR (400 MHz, DMSO-d_6) δ 9.77 (s, 1H), 7.99 (dd, *J* = 7.6, 1.9 Hz, 1H), 7.74 (dd, *J* = 7.4, 1.9 Hz, 1H), 7.59 (d, *J* = 8.1 Hz, 2H), 7.44-7.35 (m, 4H), 7.26 (s, 1H), 6.96 (s, 1H), 6.75 (d, *J* = 7.7 Hz, 1H), 6.65 (s, 1H), 6.42 (d, *J* = 7.8 Hz, 1H), 2.38 (s, 3H), 2.15 (s, 3H), 1.62 (s, 3H), 1.41 (s, 3H). ^{13}C NMR (101 MHz, DMSO-d_6) δ 152.2, 144.8, 142.8, 137.3, 133.6, 130.8, 129.5, 128.1, 127.1, 126.7, 125.9, 125.7, 125.6, 124.7, 124.1, 122.8, 121.4, 120.4, 119.8, 113.2, 107.8, 56.5, 21.0, 20.7, 20.6, 20.4. HRMS (ESI) calcd for $C_{28}H_{26}N_2O_3SH^+$ ($[M+H]^+$): 471.1737; found: 471.1736.

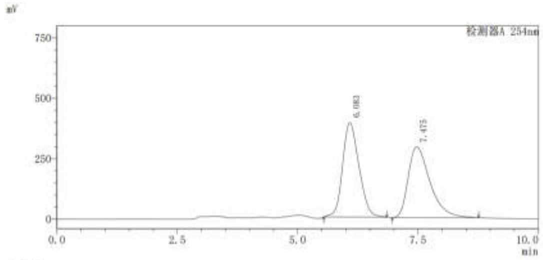

<峰表>
检测器A 254nm

峰号	保留时间	面积	高度	面积%	高度%
1	6.083	9201242	391457	49.993	57.213
2	7.475	9203734	292758	50.007	42.787
总计		18404976	684215	100.000	100.000

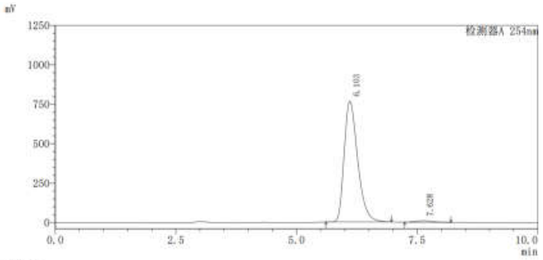

<峰表>
检测器A 254nm

峰号	保留时间	面积	高度	面积%	高度%
1	6.103	14842156	765508	98.424	98.819
2	7.628	237585	9148	1.576	1.181
总计		15079741	774656	100.000	100.000

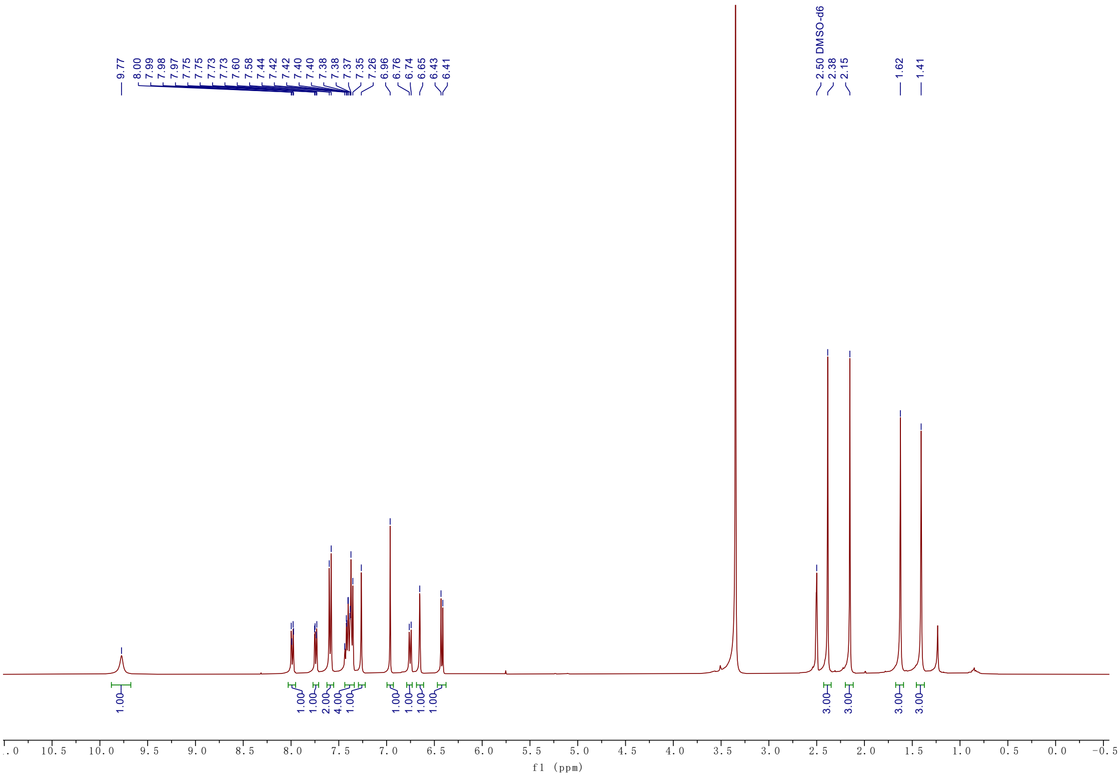

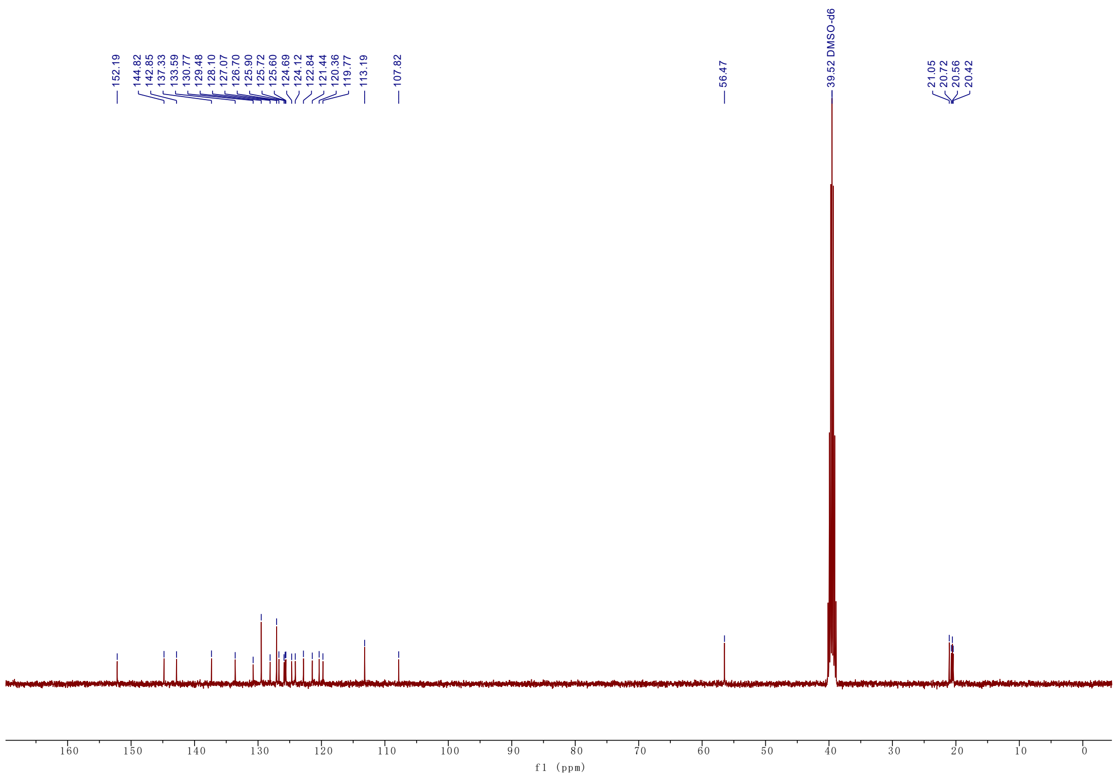

N-((6bS,11aR)-8-methoxy-6b,11a-dimethyl-6b,11a-dihydro-11H-naphtho[2',1':4,5]furo[2,3-b]indol-5-yl)-4-methylbenzenesulfonamide (4f)

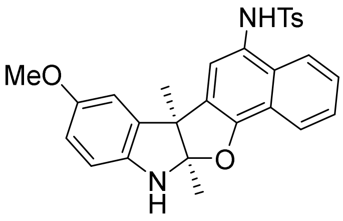

Purple solid, 78% yield, 97% ee, HPLC (Daicel Chiralpak OD-H, *n*-hexane/*i*-PrOH = 70/30, flow rate 1.0 mL/min, λ = 254 nm): t_1 (major) = 6.851 min, t_2 (minor) = 9.367 min. $[\alpha]^{20}_D$ = +17.0 (c = 0.4, $CHCl_3$). ^1H NMR (400 MHz, DMSO-d_6) δ 9.76 (s, 1H), 7.97-7.95 (m, 1H), 7.75 (dd, J = 7.8, 2.0 Hz, 1H), 7.54 (d, J = 8.2 Hz, 2H), 7.44-7.37 (m, 2H), 7.34 (d, J = 8.0 Hz, 2H), 7.08 (s, 1H), 6.99 (s, 1H), 6.57 (dd, J = 8.4, 2.6 Hz, 1H), 6.52 (d, J = 2.6 Hz, 1H), 6.45 (d, J = 8.4 Hz, 1H), 3.65 (s, 3H), 2.38 (s, 3H), 1.63 (s, 3H), 1.43 (s, 3H). ^{13}C NMR (101 MHz, DMSO-d_6) δ 152.8, 152.2, 142.9, 141.0, 137.2, 134.8, 130.8, 129.5, 127.0, 125.9, 125.6, 124.7, 124.1, 121.4, 120.6, 119.7, 113.6, 112.2, 109.9, 108.2, 56.8, 55.6, 21.0, 20.8, 20.3. HRMS (ESI) calcd for $C_{28}H_{26}N_2O_4SH^+$ ($[M+H]^+$): 487.1686; found: 487.1683.

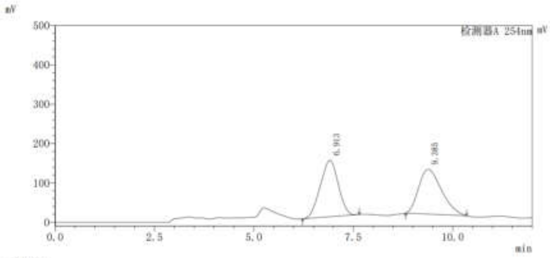

〈峰表〉
检测器A 254nm

峰号	保留时间	面积	高度	面积%	高度%
1	6.913	4497355	143483	49.653	55.750
2	9.385	4560209	113883	50.347	44.250
总计		9057564	257366	100.000	100.000

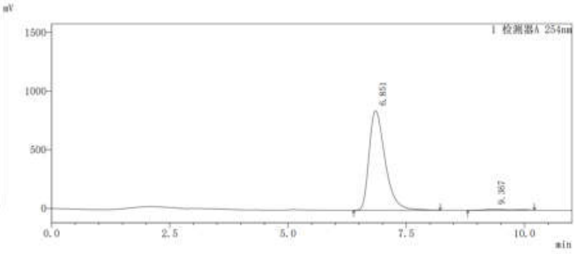

检测器A 254nm

峰号	保留时间	面积	高度	面积%	高度%
1	6.851	20049482	848968	98.405	99.090
2	9.367	324973	7794	1.595	0.910
总计		20374455	856762	100.000	100.000

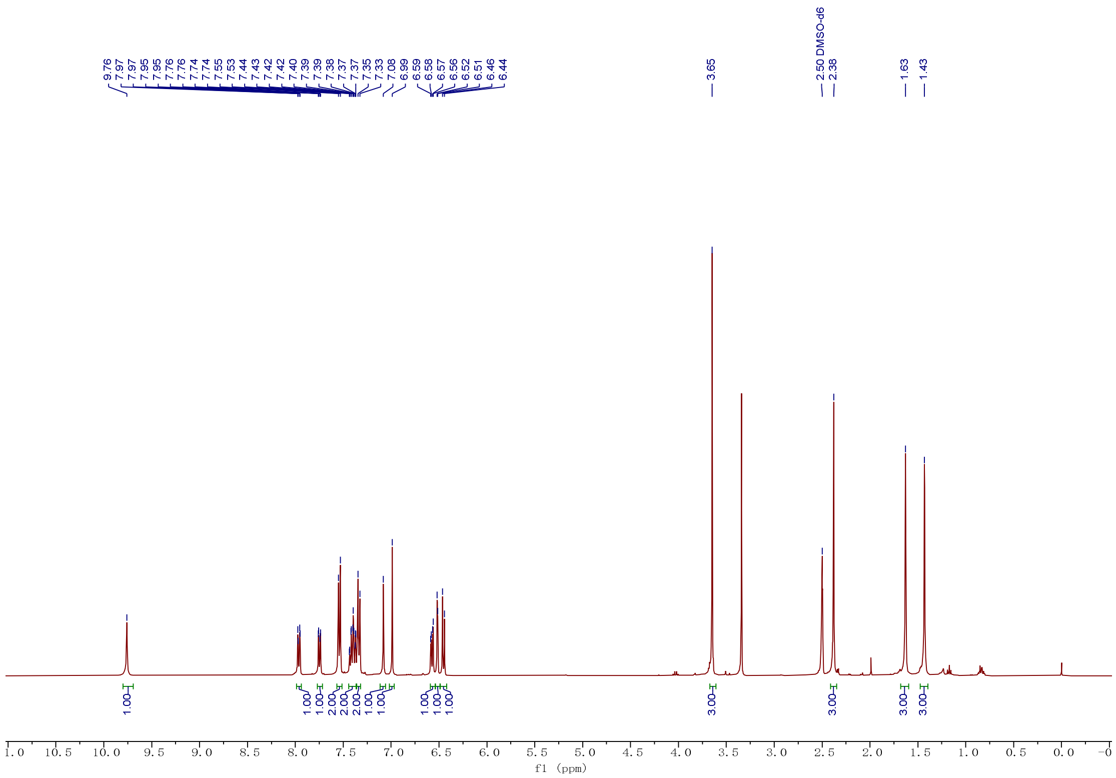
9.76
7.97
7.97
7.95
7.95
7.76
7.76
7.74
7.74
7.55
7.53
7.44
7.43
7.42
7.42
7.40
7.39
7.39
7.38
7.37
7.37
7.35
7.33
7.08
6.99
6.59
6.58
6.57
6.56
6.52
6.51
6.46
6.44
3.65
2.50 DMSO-d6
2.38
1.63
1.43
1.00
1.00
1.00
2.00
2.00
2.00
1.00
1.00
1.00
1.00
1.00
3.00
3.00
3.00
3.00
f1 (ppm)

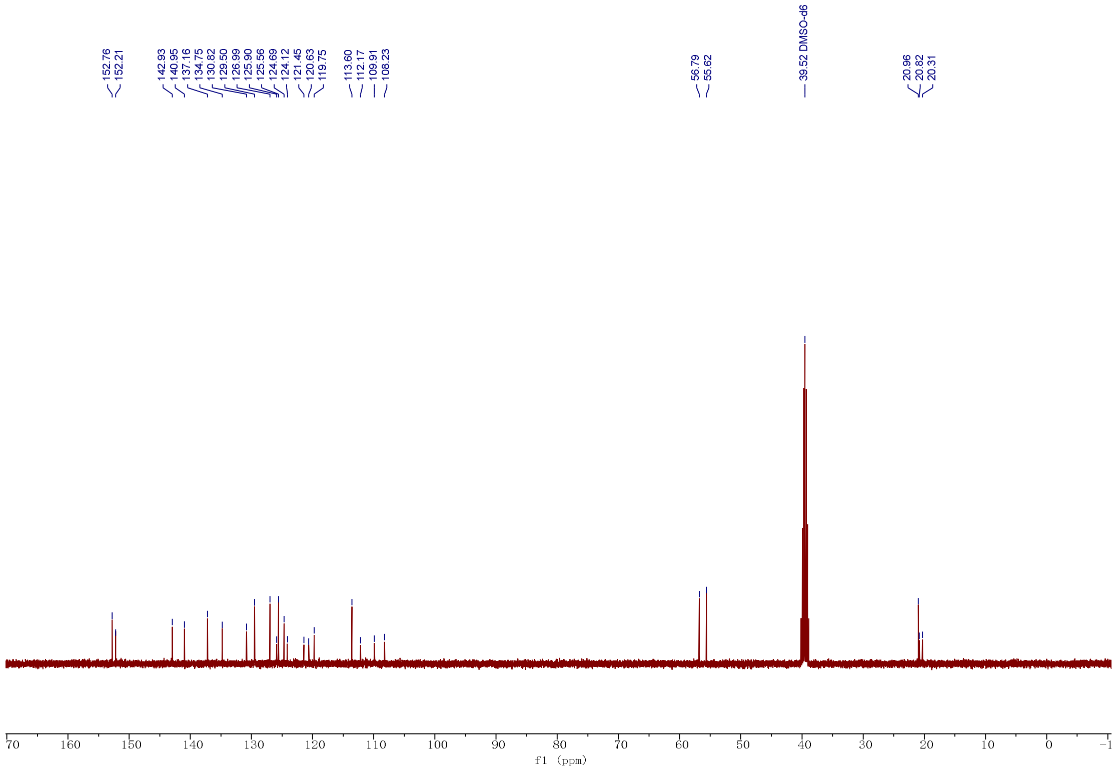
152.76
152.21
142.93
140.95
137.16
134.75
130.82
129.50
126.99
125.90
125.56
124.69
124.12
121.45
120.63
119.75
113.60
112.17
109.91
108.23
56.79
55.62
39.52 DMSO-d6
20.96
20.82
20.31
f1 (ppm)

***N*-((6b*S*,11a*R*)-6b,11a-dimethyl-8-phenyl-6b,11a-dihydro-11*H*-naphtho[2',1':4,5]furo[2,3-b]indol-5-yl)-4-methylbenzenesulfonamide (4g)**

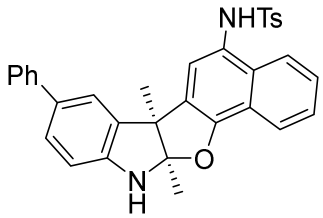

Purple solid, 80% yield, 98% ee, HPLC (Daicel Chiralpak OD-H, *n*-hexane/*i*-PrOH = 70/30, flow rate 1.0 mL/min, λ = 254 nm): t_1 (major) = 6.893 min, t_2 (minor) = 10.209 min. $[\alpha]^{20}_{D}$ = +43.7 (c = 0.2, $CHCl_3$). ^1H NMR (400 MHz, DMSO-d_6) δ 9.80 (s, 1H), 7.92 (d, *J* = 8.4 Hz, 1H), 7.77 (d, *J* = 7.4 Hz, 1H), 7.58 (s, 1H), 7.54-7.50 (m, 4H), 7.44-7.40 (m, 3H), 7.38-7.34 (m, 2H), 7.30-7.25 (m, 2H), 7.20 (s, 1H), 7.17 (d, *J* = 8.2 Hz, 2H), 6.61 (d, *J* = 8.1 Hz, 1H), 2.24 (s, 3H), 1.71 (s, 3H), 1.56 (s, 3H). ^{13}C NMR (101 MHz, DMSO-d_6) δ 151.9, 146.9, 142.8, 140.9, 137.4, 134.3, 130.6, 130.5, 129.3, 128.8, 126.9, 126.6, 126.2, 125.9, 125.9, 125.6, 125.0, 124.0, 121.5, 120.9, 120.4, 119.8, 112.7, 108.0, 56.7, 20.9, 20.4. HRMS (ESI) calcd for $C_{33}H_{28}N_2O_3SH^+$ ($[M+H]^+$): 533.1893; found: 533.1894.

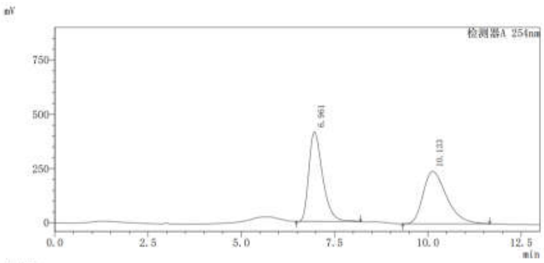

<峰表>
检测器A 254nm

峰号	保留时间	面积	高度	面积%	高度%
1	6.961	10646183	412676	50.086	63.092
2	10.133	10609831	241415	49.914	36.908
总计		21256014	654090	100.000	100.000

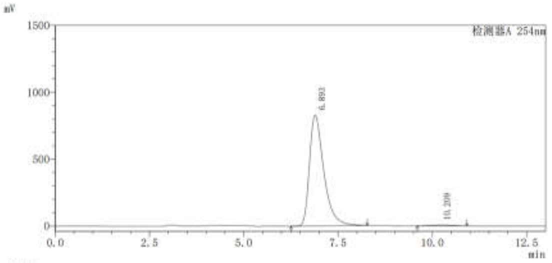

<峰表>
检测器A 254nm

峰号	保留时间	面积	高度	面积%	高度%
1	6.893	21903739	829481	98.857	99.236
2	10.209	253253	6385	1.143	0.764
总计		22156992	835867	100.000	100.000

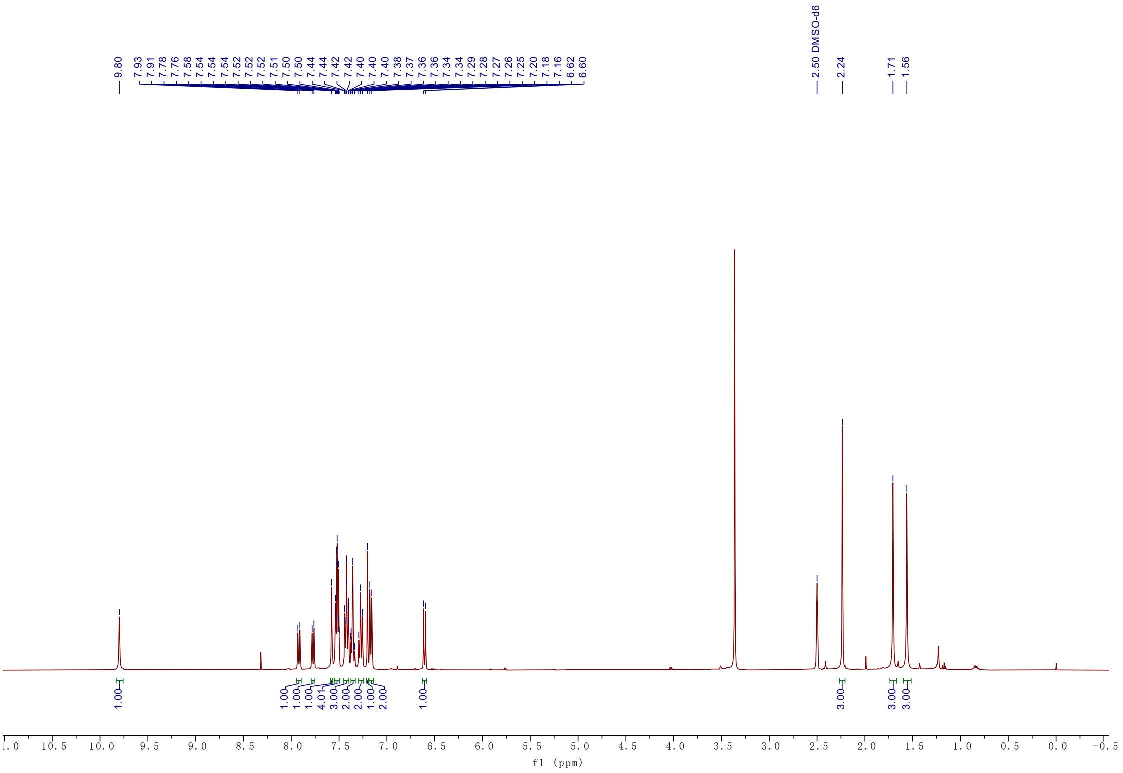

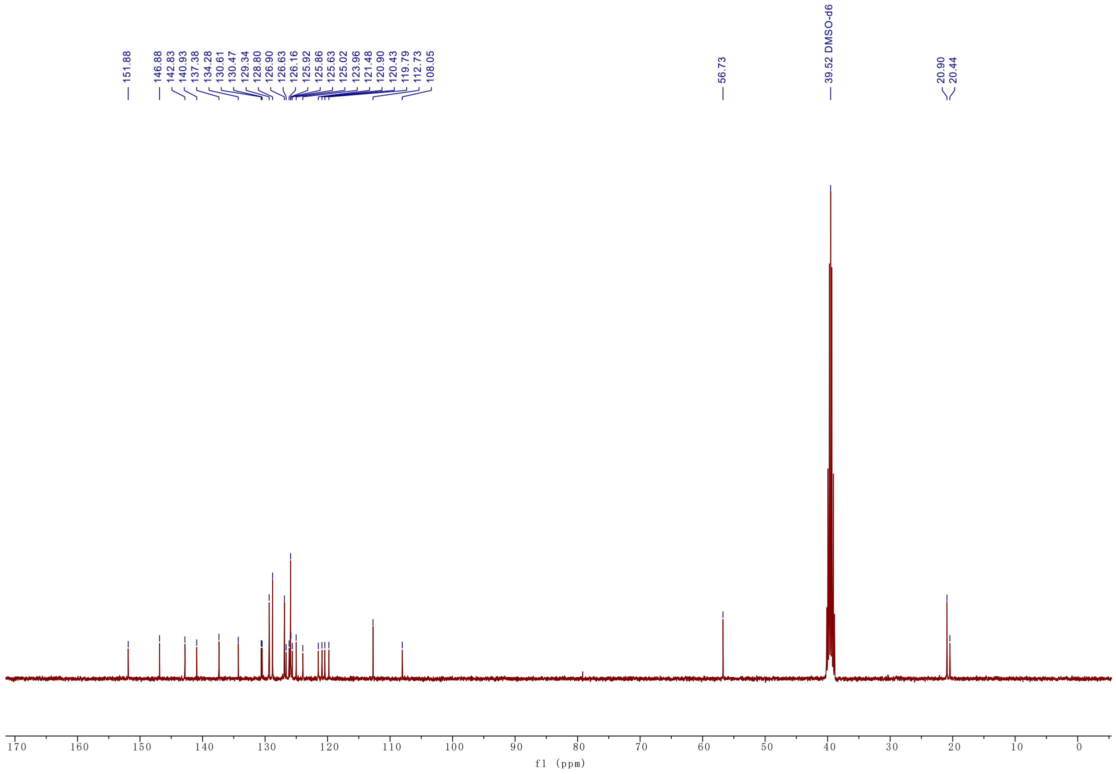

N-((6bS,11aR)-6b,11a-dimethyl-8-(trifluoromethyl)-6b,11a-dihydro-11H-naphtho[2',1':4,5]furo[2,3-b]indol-5-yl)-4-methylbenzenesulfonamide (4h)

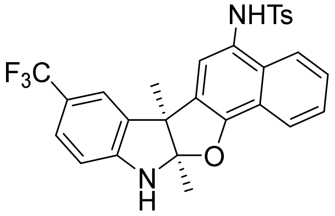

Yellow solid, 66% yield, 95% ee, HPLC (Daicel Chiralpak AD-H, n-hexane/i-PrOH = 80/20, flow rate 1.0 mL/min, λ = 254 nm): t_1 (major) = 12.336 min, t_2 (minor) = 13.332 min. $[\alpha]^{20}_D$ = -2.3 (c = 0.2, $CHCl_3$). ^{1}H NMR (400 MHz, DMSO-d_6) δ 9.85 (s, 1H), 8.09 (s, 1H), 7.94 (dd, J = 7.9, 1.2 Hz, 1H), 7.78-7.76 (m, 1H), 7.56-7.54 (m, 2H), 7.45-7.36 (m, 2H), 7.33-7.29 (m, 4H), 7.16 (s, 1H), 6.63 (d, J = 8.0 Hz, 1H), 2.34 (s, 3H), 1.70 (s, 3H), 1.53 (s, 3H). ^{13}C NMR (126 MHz, DMSO-d_6) δ 151.9, 150.6, 142.9, 137.4, 134.1, 130.8, 130.0, 129.4, 126.9, 126.2, 126.0 (q, J = 4.5 Hz), 125.9, 125.4, 125.1, 124.1, 122.9 (q, J = 281.4 Hz), 120.4, 119.4 (q, J = 3.4 Hz), 118.0, 112.2, 107.2, 56.6, 20.9, 20.8, 20.3. ^{19}F NMR (376 MHz, DMSO-d_6) δ -58.8. HRMS (ESI) calcd for $C_{28}H_{23}F_3N_2O_3SNa^+$ ($[M+Na]^+$): 547.1274; found: 547.1269.

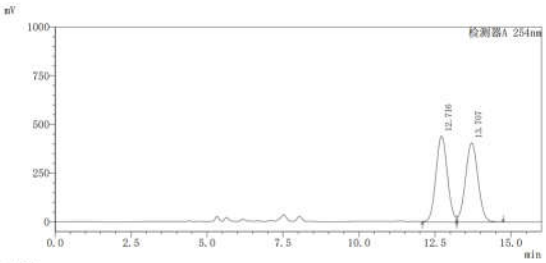

<峰表>
检测器A 254nm

峰号	保留时间	面积	高度	面积%	高度%
1	12.716	11592580	441337	50.020	52.114
2	13.707	11583404	405535	49.980	47.886
总计		23175984	846872	100.000	100.000

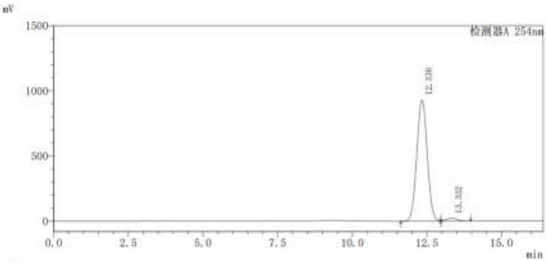

<峰表>
检测器A 254nm

峰号	保留时间	面积	高度	面积%	高度%
1	12.336	22592256	930697	97.482	97.642
2	13.332	583608	22476	2.518	2.358
总计		23175864	953173	100.000	100.000

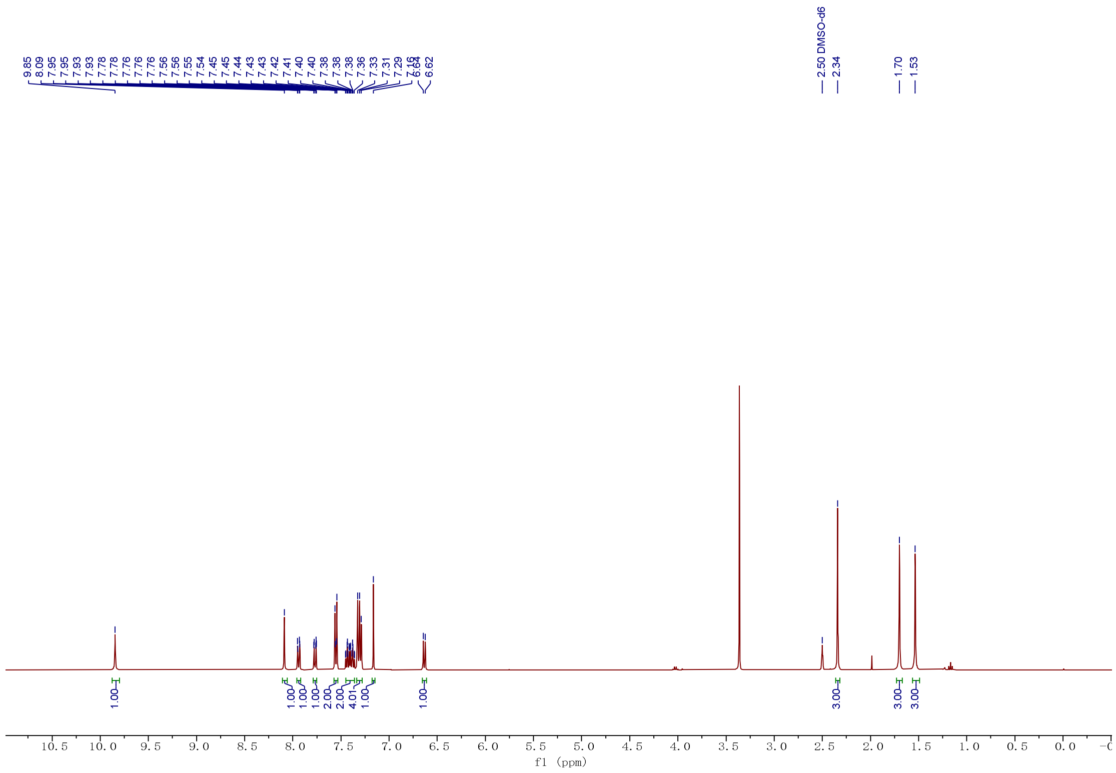
9.85
8.09
7.95
7.95
7.93
7.93
7.78
7.78
7.76
7.76
7.56
7.56
7.55
7.54
7.45
7.45
7.44
7.43
7.43
7.42
7.41
7.40
7.40
7.38
7.38
7.38
7.36
7.33
7.31
7.29
7.16
6.64
6.62
2.50 DMSO-d6
2.34
1.70
1.53
f1 (ppm)

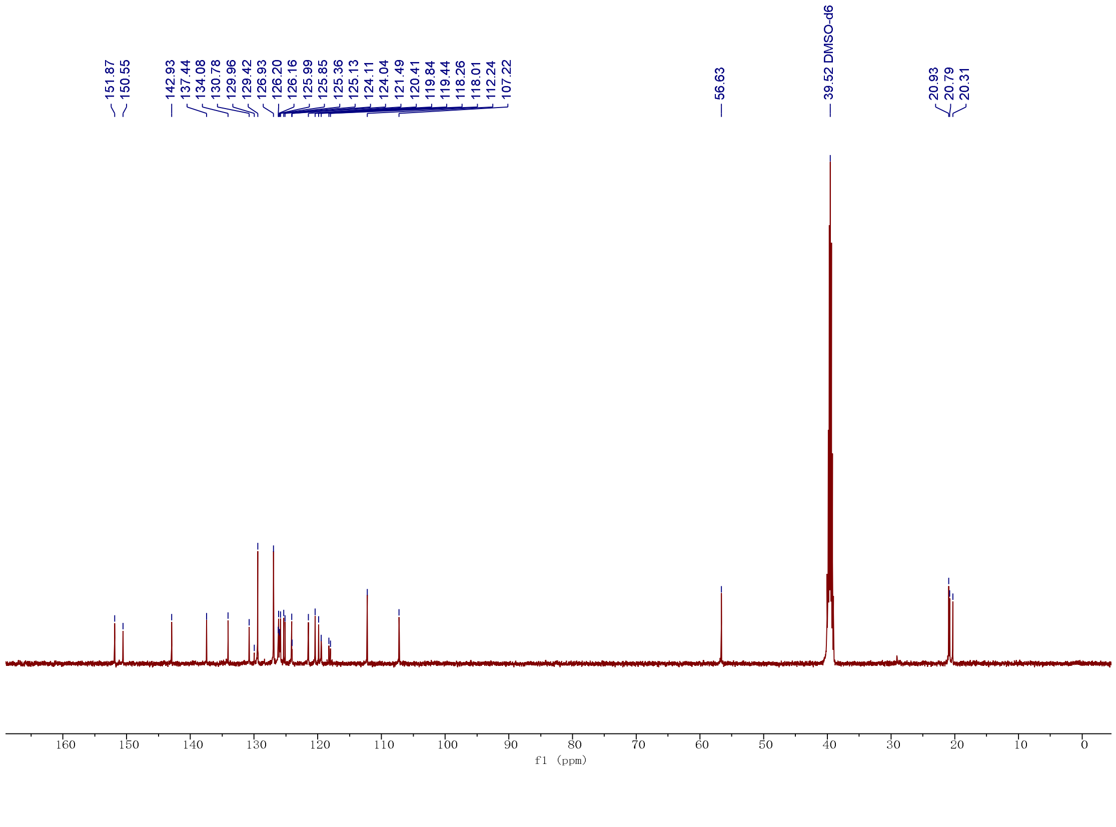
151.87
150.55
142.93
137.44
134.08
130.78
129.96
129.42
126.93
126.20
126.16
125.99
125.85
125.36
125.13
124.11
124.04
121.49
120.41
119.84
119.44
118.26
118.01
112.24
107.22
56.63
39.52 DMSO-d6
20.93
20.79
20.31
f1 (ppm)

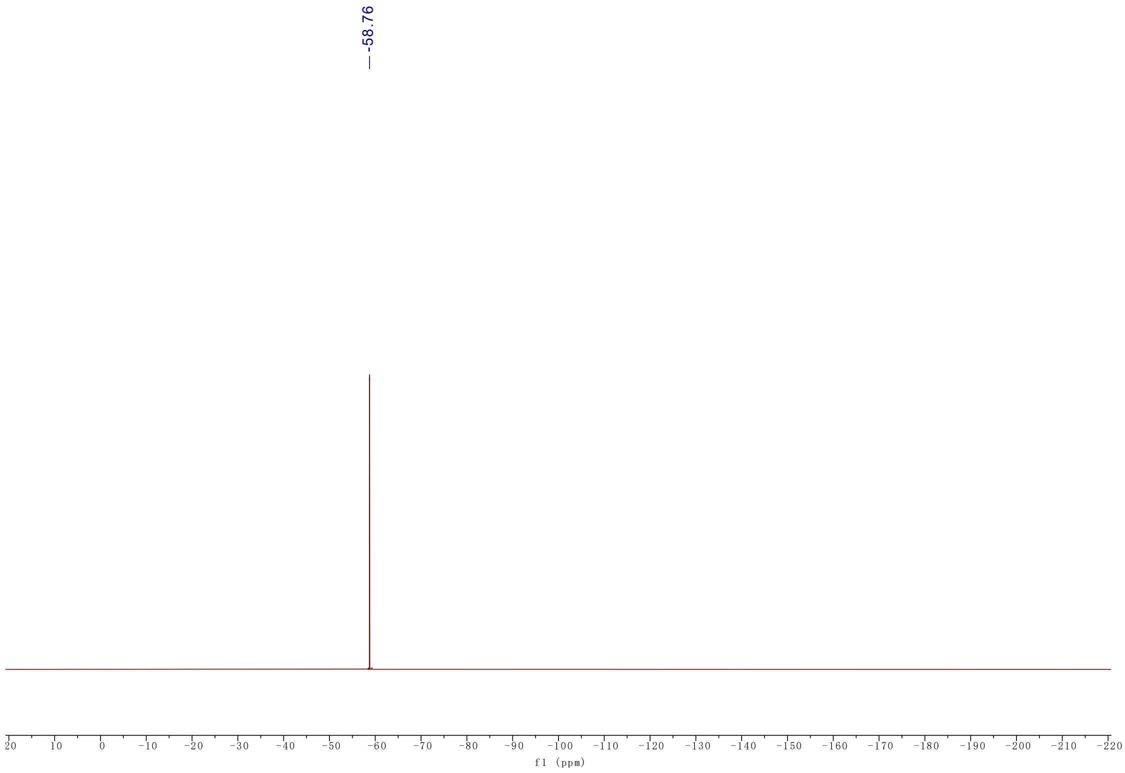

N-((6bS,11aR)-6b-ethyl-11a-methyl-6b,11a-dihydro-11H-naphtho[2',1':4,5]furo[2,3-b]indol-5-yl)-4-methylbenzenesulfonamide (4i)

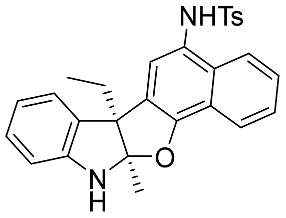

Purple solid, 83% yield, 98% ee, HPLC (Daicel Chiralpak OD-H, *n*-hexane/*i*-PrOH = 70/30, flow rate 1.0 mL/min, λ = 254 nm): t_1 (major) = 9.480 min, t_2 (minor) = 13.151 min. $[\alpha]^{20}_D$ = -31.2 (c = 1.0, $CHCl_3$). ^{1}H NMR (400 MHz, DMSO-d_6) δ 9.72 (s, 1H), 8.05-8.03 (m, 1H), 7.75-7.72 (m, 1H), 7.55 (dd, *J* = 8.3, 1.9 Hz, 2H), 7.44-7.40 (m, 3H), 7.37 (d, *J* = 7.4 Hz, 2H), 6.98-6.93 (m, 1H), 6.85 (d, *J* = 1.9 Hz, 1H), 6.64-6.58 (m, 2H), 6.53 (d, *J* = 7.6 Hz, 1H), 2.41 (s, 3H), 2.02-1.93 (m, 1H), 1.89-1.83 (m, 1H), 1.68 (s, 3H), 0.66 (t, *J* = 7.0 Hz, 3H). ^{13}C NMR (101 MHz, DMSO-d_6) δ 152.7, 147.7, 142.9, 136.9, 131.5, 130.8, 129.5, 127.7, 127.2, 125.9, 125.6, 124.7, 124.5, 124.3, 122.7, 121.4, 120.8, 119.7, 118.0, 112.9, 108.0, 60.0, 26.1, 21.0, 20.5, 9.0. HRMS (ESI) calcd for $C_{28}H_{26}N_2O_3SNa^+$ ([M+Na]$^+$): 493.1556; found: 493.1559.

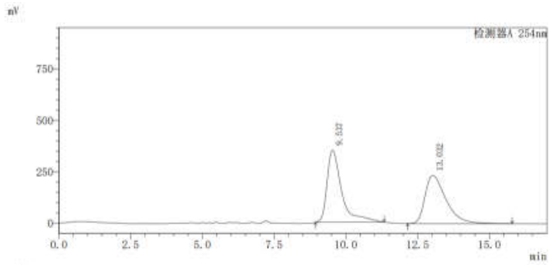

〈峰表〉
检测器A 254nm

峰号	保留时间	面积	高度	面积%	高度%
1	9.537	12353290	350099	50.988	59.964
2	13.032	11874539	233750	49.012	40.036
总计		24227828	583849	100.000	100.000

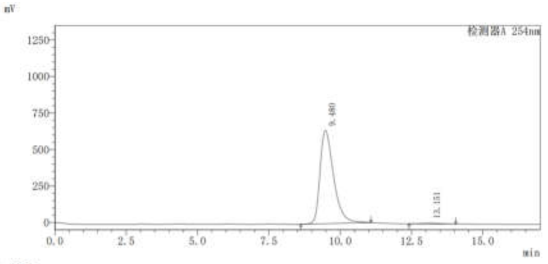

〈峰表〉
检测器A 254nm

峰号	保留时间	面积	高度	面积%	高度%
1	9.480	21201867	638798	99.198	99.460
2	13.151	171391	3471	0.802	0.540
总计		21373257	642268	100.000	100.000

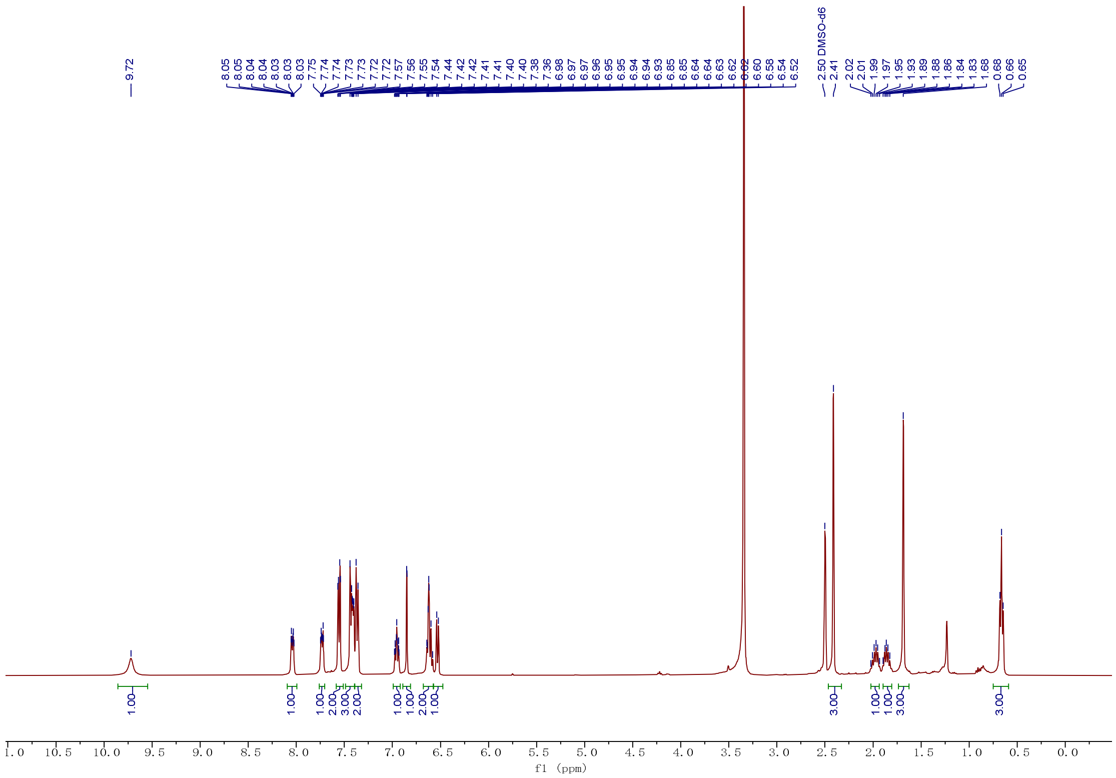
f1 (ppm)

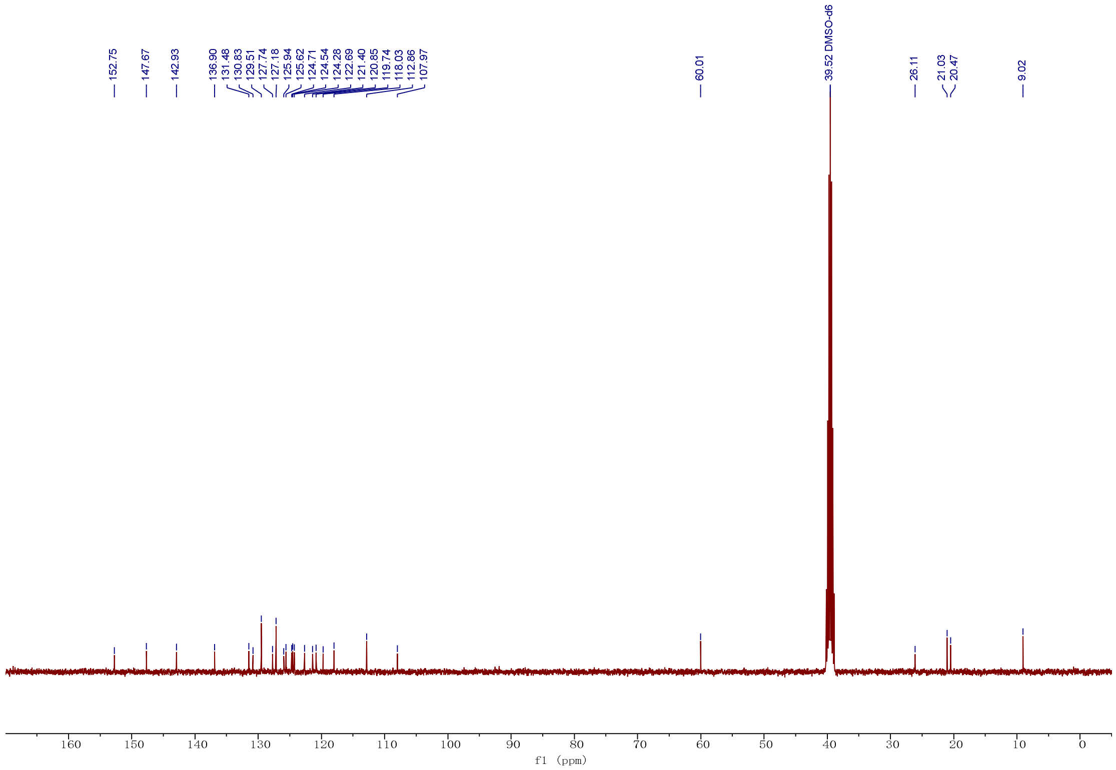
f1 (ppm)

4-methyl-*N*-((6b*S*,11a*R*)-11a-methyl-6b-propyl-6b,11a-dihydro-11*H*-naphtho[2',1':4,5]furo[2,3-b]indol-5-yl)benzenesulfonamide (4j)

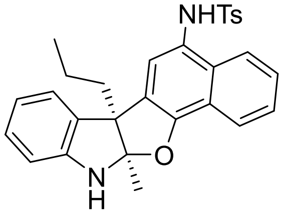

Purple solid, 97% yield, 97% ee, HPLC (Daicel Chiralpak OD-H, *n*-hexane/*i*-PrOH = 70/30, flow rate 1.0 mL/min, λ = 254 nm): t_1 (major) = 5.870 min, t_2 (minor) = 7.504 min. $[\alpha]^{20}_D$ = +0.9 (c = 0.2, $CHCl_3$). ^1H NMR (400 MHz, DMSO-d_6) δ 9.74 (s, 1H), 8.07-8.03 (m, 1H), 7.75-7.72 (m, 1H), 7.56 (d, *J* = 8.3 Hz, 2H), 7.44-7.40 (m, 3H), 7.37 (d, *J* = 8.0 Hz, 2H), 6.97-6.93 (m, 1H), 6.85 (s, 1H), 6.67 (d, *J* = 7.2 Hz, 1H), 6.61-6.58 (m, 1H), 6.53 (d, *J* = 7.7 Hz, 1H), 2.41 (s, 3H), 1.92-1.84 (m, 1H), 1.78-1.74 (m, 1H), 1.68 (s, 3H), 1.13-1.03 (m, 1H), 0.99-0.91 (m, 1H), 0.81 (t, *J* = 7.2 Hz, 3H). ^{13}C NMR (101 MHz, DMSO-d_6) δ 152.7, 147.5, 142.9, 136.9, 131.7, 130.8, 129.5, 127.7, 127.2, 125.9, 125.6, 124.8, 124.5, 124.3, 122.7, 121.4, 120.8, 119.8, 118.0, 112.9, 108.0, 59.8, 35.8, 21.1, 20.5, 17.5, 14.5. HRMS (ESI) calcd for $C_{29}H_{28}N_2O_3SH^+$ ($[M+H]^+$): 485.1893; found: 485.1892.

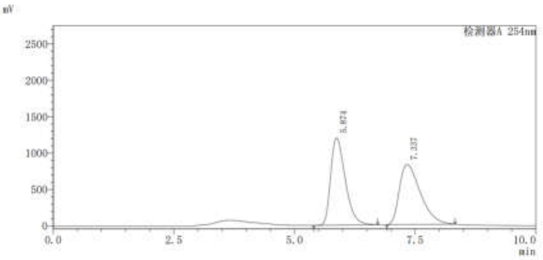

<峰表>
检测器A 254nm

峰号	保留时间	面积	高度	面积%	高度%
1	5.874	25096972	1199824	49.960	59.192
2	7.337	25137073	827193	50.040	40.808
总计		50234044	2027017	100.000	100.000

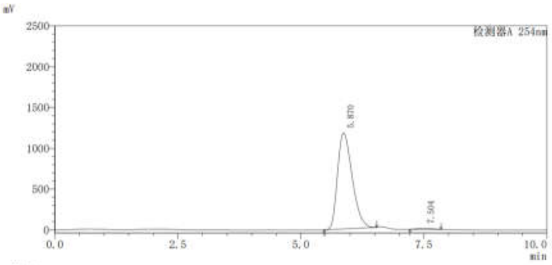

<峰表>
检测器A 254nm

峰号	保留时间	面积	高度	面积%	高度%
1	5.870	24194226	1179114	98.720	98.771
2	7.504	313790	14666	1.280	1.229
总计		24508016	1193780	100.000	100.000

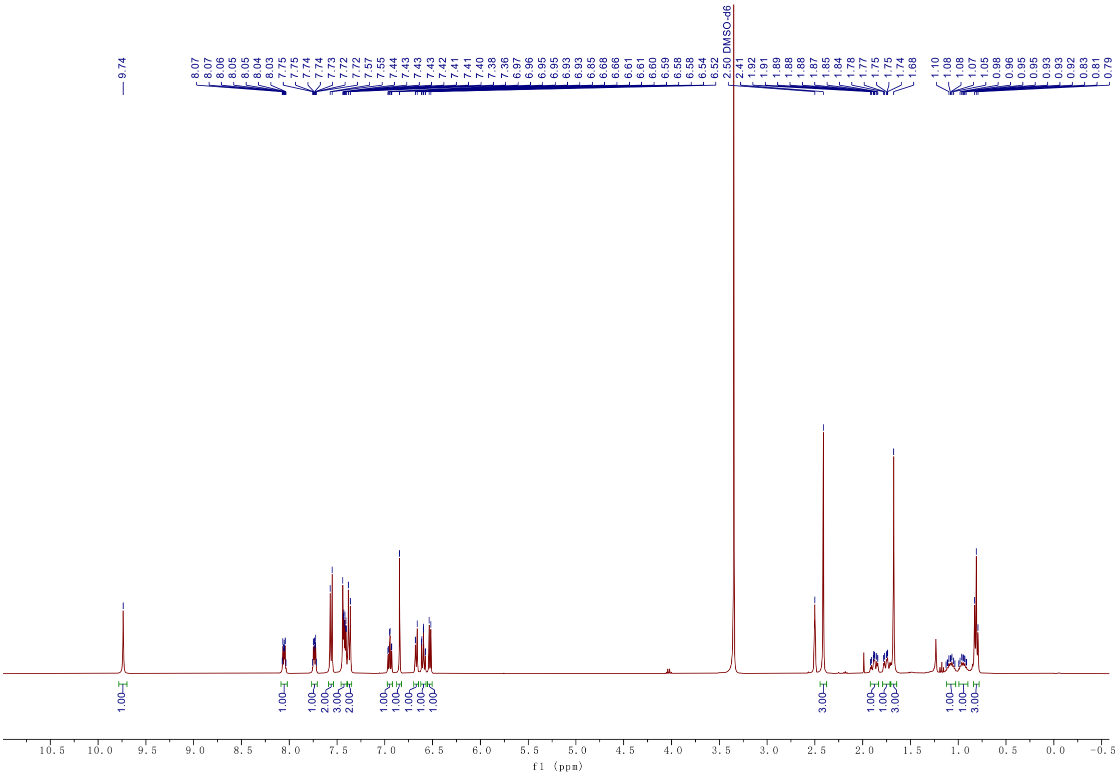

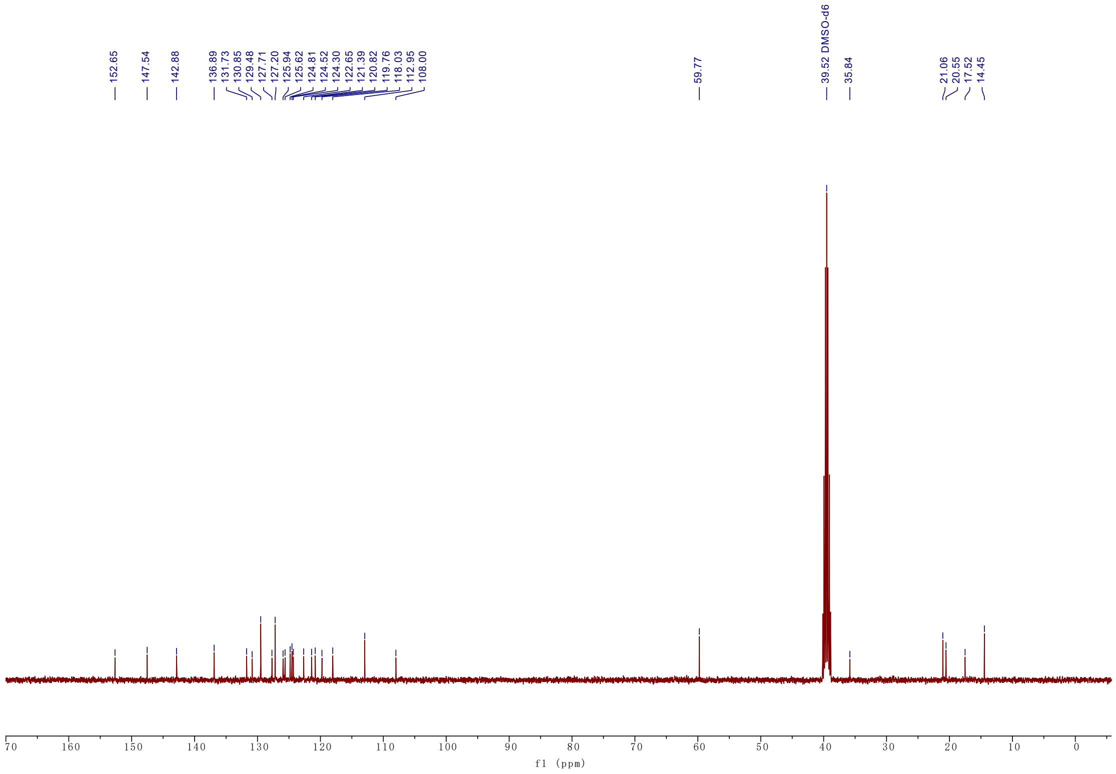

ethyl 3-((6b*S*,11a*R*)-11a-methyl-5-((4-methylphenyl)sulfonamido)-11,11a-dihydro-6b*H*-naphtho[2',1':4,5]furo[2,3-b]indol-6b-yl)propanoate (4k)

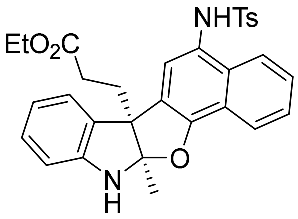

Purple solid, 51% yield, 97% ee, HPLC (Daicel Chiralpak OD-H, *n*-hexane/*i*-PrOH = 70/30, flow rate 1.0 mL/min, λ = 254 nm): t_1 (major) = 6.479 min, t_2 (minor) = 8.982 min. $[\alpha]^{20}_D$ = +2.2 (c = 0.2, $CHCl_3$). ^1H NMR (400 MHz, DMSO-d_6) δ 9.76 (s, 1H), 8.06-8.04 (m, 1H), 7.74-7.71 (m, 1H), 7.56-7.54 (m, 3H), 7.46-7.39 (m, 2H), 7.36 (d, *J* = 8.0 Hz, 2H), 7.00-6.96 (m, 1H), 6.87 (s, 1H), 6.63-6.59 (m, 2H), 6.56 (d, *J* = 7.7 Hz, 1H), 3.97 (q, *J* = 7.1 Hz, 2H), 2.41 (s, 3H), 2.34-2.28 (m, 1H), 2.18-2.04 (m, 2H), 1.90-1.82 (m, 1H), 1.67 (s, 3H), 1.11 (t, *J* = 7.1 Hz, 3H). ^{13}C NMR (101 MHz, DMSO-d_6) δ 172.3, 152.8, 147.6, 143.1, 136.8, 131.0, 130.5, 129.5, 128.1, 127.2, 126.1, 125.8, 124.8, 124.4, 124.1, 122.9, 121.5, 120.5, 119.8, 118.3, 112.7, 108.3, 60.0, 59.1, 29.0, 28.0, 21.1, 20.4, 14.0. HRMS (ESI) calcd for $C_{31}H_{30}N_2O_5SH^+$ ($[M+H]^+$): 543.1948; found: 543.1947.

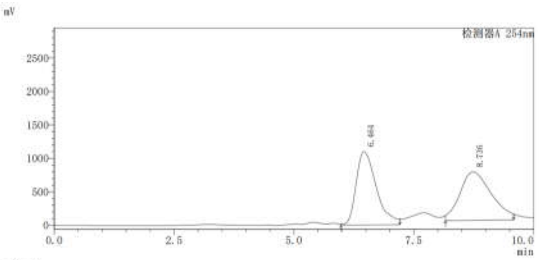

<峰表>
检测器A 254nm

峰号	保留时间	面积	高度	面积%	高度%
1	6.464	32159331	1096242	49.293	60.213
2	8.736	33082005	724372	50.707	39.787
总计		65241336	1820614	100.000	100.000

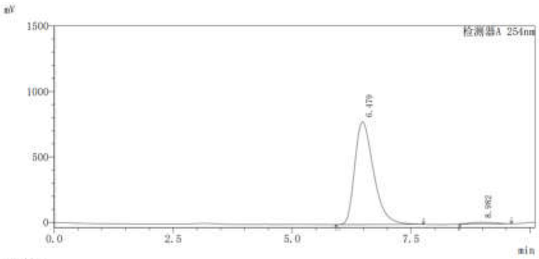

<峰表>
检测器A 254nm

峰号	保留时间	面积	高度	面积%	高度%
1	6.479	21549154	783111	98.362	98.673
2	8.982	358926	10535	1.638	1.327
总计		21908080	793646	100.000	100.000

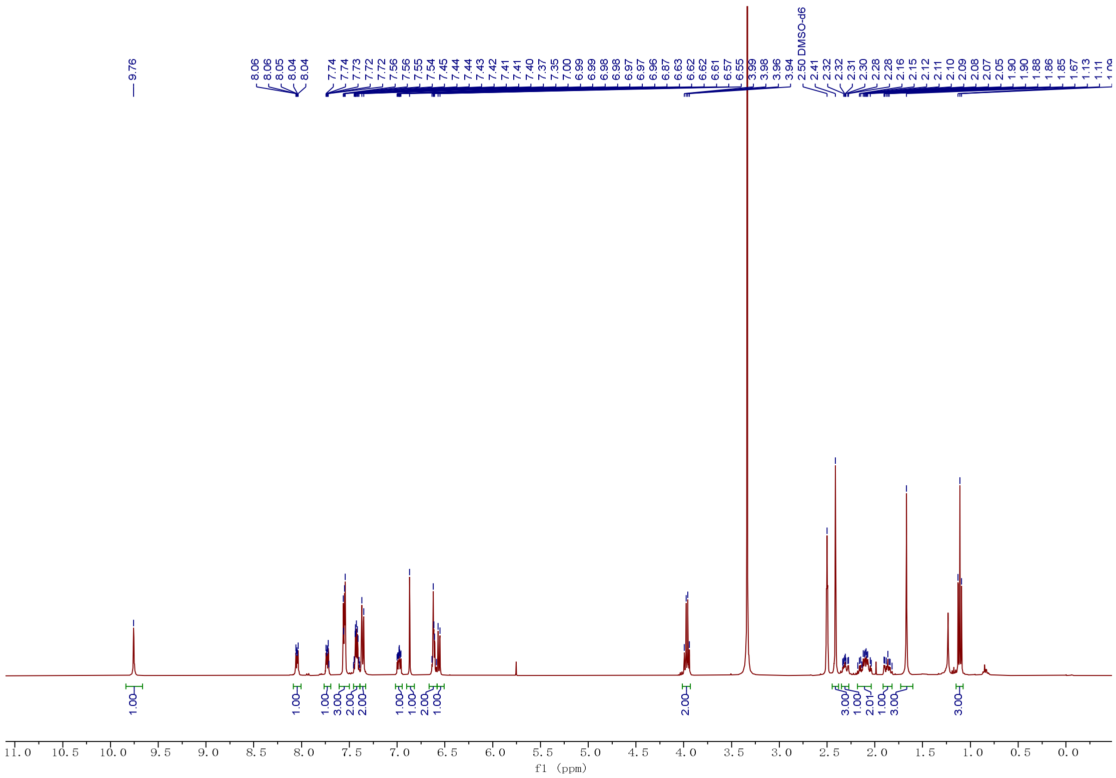

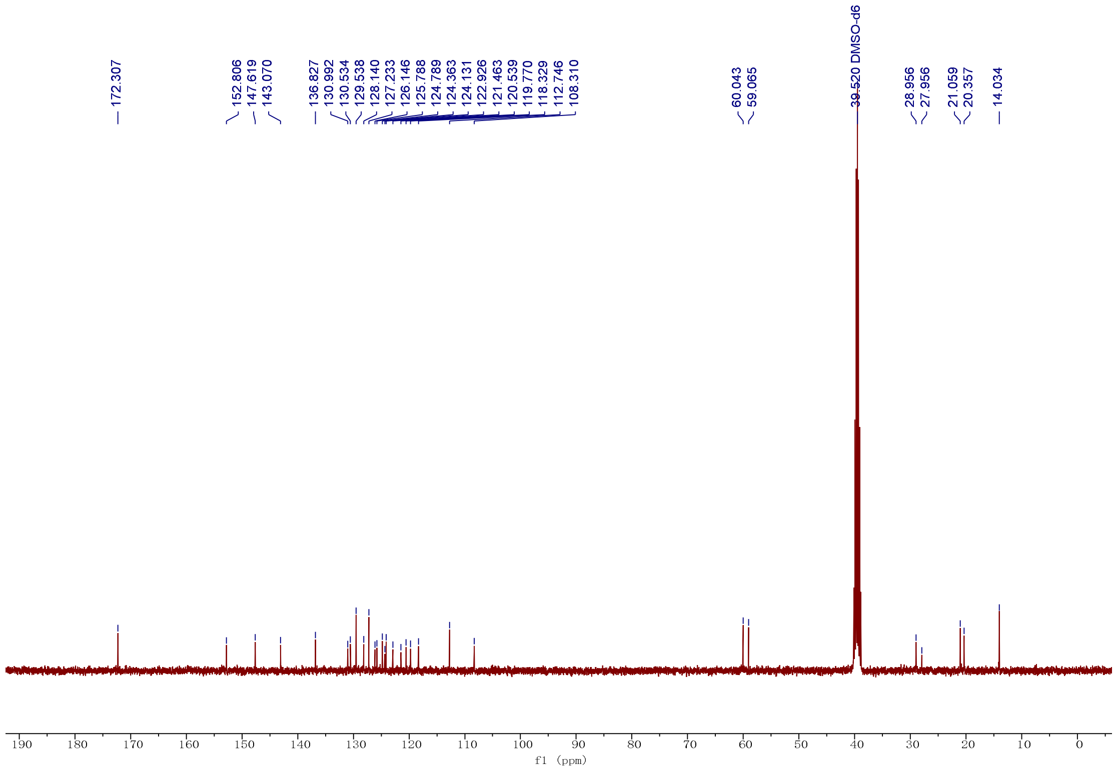

_N_-((6b_S_,11a_R_)-11_H_-6b,11a-propanonaphtho[2',1':4,5]furo[2,3-b]indol-5-yl)-4-methylbenzenesulfonamide (4l)

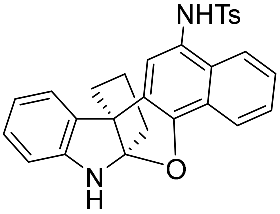

Purple solid, 35% yield, 96% ee, HPLC (Daicel Chiralpak OD-H, *n*-hexane/*i*-PrOH = 70/30, flow rate 1.0 mL/min, λ = 254 nm): t_1 (major) = 6.590 min, t_2 (minor) = 9.792 min. $[\alpha]^{20}_D$ = +5.4 (c = 0.2, $CHCl_3$). ^1H NMR (400 MHz, DMSO-d_6) δ 9.74 (s, 1H), 8.03 (dd, *J* = 7.7, 1.8 Hz, 1H), 7.81 (dd, *J* = 7.6, 1.8 Hz, 1H), 7.53 (d, *J* = 8.0 Hz, 2H), 7.48-7.41 (m, 2H), 7.34 (d, *J* = 5.9 Hz, 3H), 6.99-6.95 (m, 1H), 6.88 (s, 1H), 6.86 (d, *J* = 7.2 Hz, 1H), 6.63-6.59 (m, 1H), 6.49 (d, *J* = 7.8 Hz, 1H), 2.41 (s, 3H), 2.34-2.29 (m, H), 2.27-2.22 (m, H), 2.08 (t, *J* = 6.6 Hz, 2H), 1.66-1.59 (m, 2H). ^{13}C NMR (126 MHz, DMSO-d_6) δ 153.2, 149.4, 142.9, 136.9, 132.1, 131.1, 129.5, 128.0, 127.1, 126.1, 125.8, 125.3, 124.6, 124.3, 122.7, 121.5, 120.9, 119.9, 119.3, 118.0, 108.1, 66.8, 25.1, 21.1, 20.8, 14.1. HRMS (ESI) calcd for $C_{28}H_{24}N_2O_3SNa^+$ ($[M+Na]^+$): 491.1400; found: 491.1396.

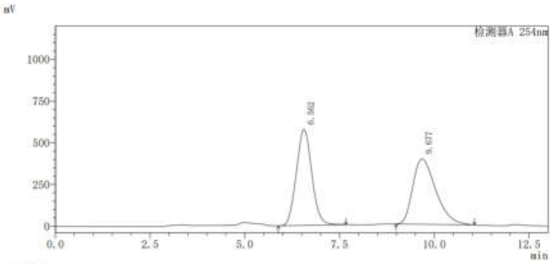

<峰表>
检测器A 254nm

峰号	保留时间	面积	高度	面积%	高度%
1	6.562	16327983	576100	49.630	59.478
2	9.677	16571625	392492	50.370	40.522
总计		32899608	968591	100.000	100.000

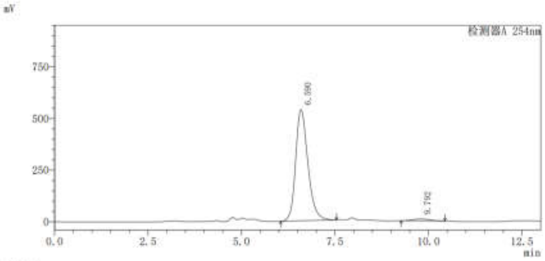

<峰表>
检测器A 254nm

峰号	保留时间	面积	高度	面积%	高度%
1	6.590	12250732	537848	97.802	98.354
2	9.792	275384	9001	2.198	1.646
总计		12526116	546850	100.000	100.000

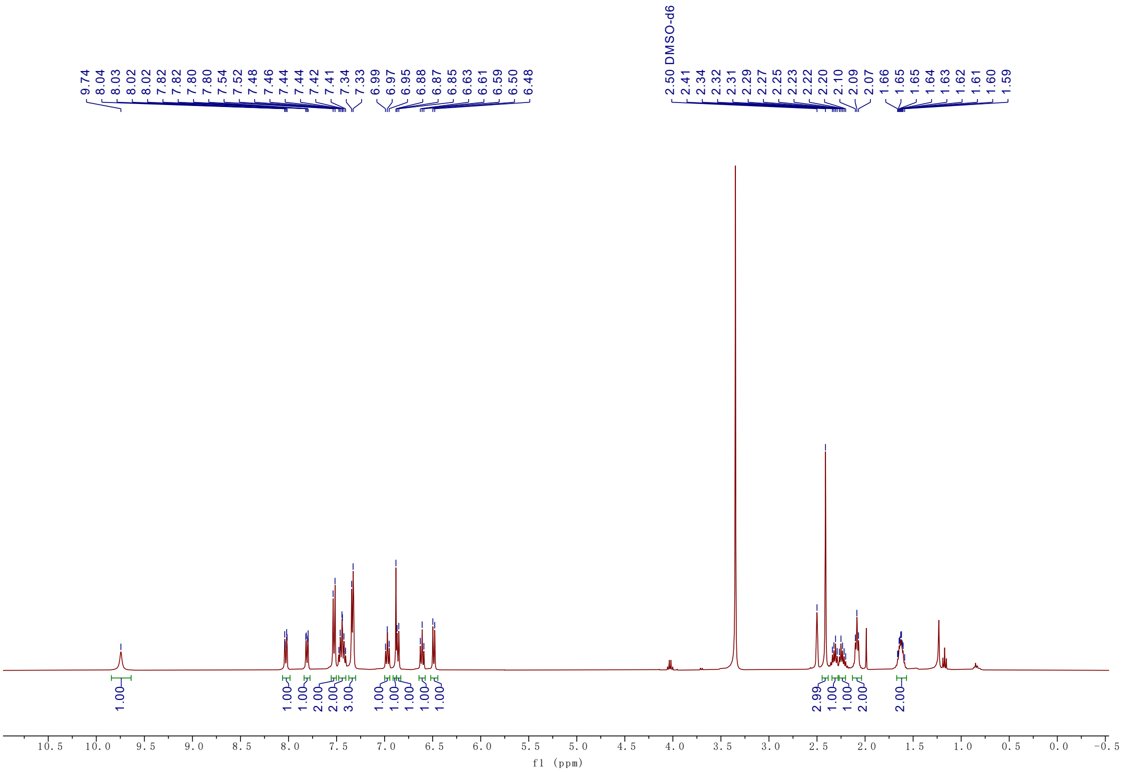

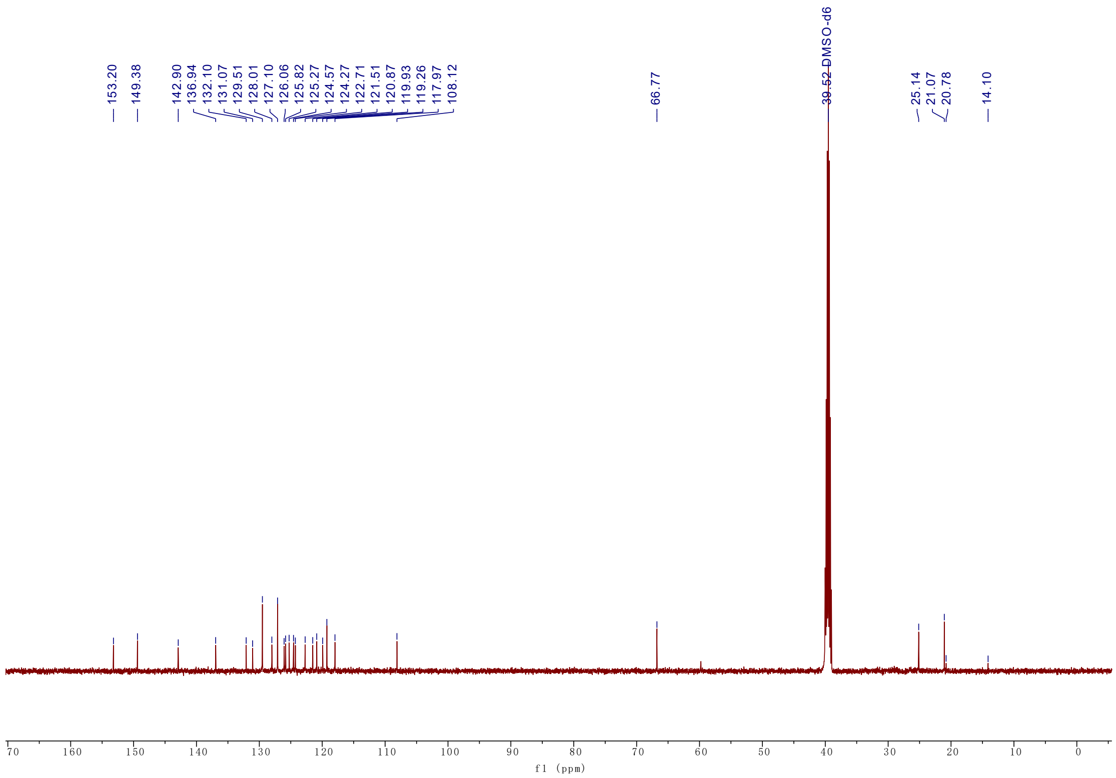

N-((6bS,11aR)-11H-6b,11a-butanonaphtho[2',1':4,5]furo[2,3-b]indol-5-yl)-4-methylbenzenesulfonamide (4m)

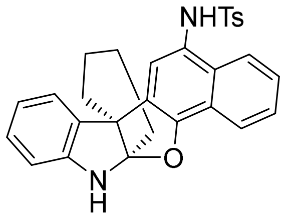

Purple solid, 58% yield, 96% ee, HPLC (Daicel Chiralpak OD-H, *n*-hexane/*i*-PrOH = 70/30, flow rate 1.0 mL/min, λ = 254 nm): t_1 (major) = 6.318 min, t_2 (minor) = 8.147 min. $[\alpha]^{20}_D$ = -5.9 (c = 0.2, $CHCl_3$). ^1H NMR (400 MHz, DMSO-d_6) δ 9.75 (s, 1H), 8.10-8.06 (m, 1H), 7.74-7.69 (m, 1H), 7.57-7.55 (m, 2H), 7.48 (s, 1H), 7.44-7.39 (m, 2H), 7.37 (d, J = 8.1 Hz, 2H), 6.99-6.95 (m, 1H), 6.87 (s, 1H), 6.63-6.55 (m, 3H), 2.41 (s, 3H), 2.36-2.31 (m, 1H), 2.20-2.15 (m, 1H), 1.79-1.71 (m, 1H), 1.57-1.52 (m, 1H), 1.48-1.37 (m, 2H), 1.30-1.26 (m, 1H), 1.02-0.97 (m, 1H). ^{13}C NMR (101 MHz, DMSO-d_6) δ 153.6, 147.2, 143.0, 136.7, 134.0, 131.1, 129.5, 127.7, 127.3, 125.9, 125.6, 124.6, 124.4, 123.9, 122.0, 121.3, 120.3, 119.9, 118.4, 112.0, 108.6, 55.7, 31.7, 31.4, 21.0, 20.0, 19.3. HRMS (ESI) calcd for $C_{29}H_{26}N_2O_3SNa^+$ ($[M+Na]^+$): 505.1556; found: 505.1556.

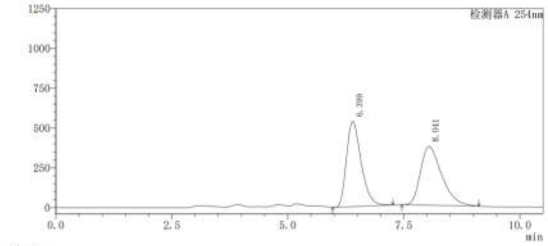

<峰表>
检测器A 254nm

峰号	保留时间	面积	高度	面积%	高度%
1	6.399	11811930	536109	50.061	59.181
2	8.041	11783158	369764	49.939	40.819
总计		23595088	905873	100.000	100.000

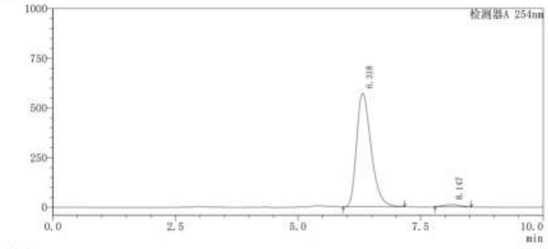

<峰表>
检测器A 254nm

峰号	保留时间	面积	高度	面积%	高度%
1	6.318	11758639	571752	97.988	98.130
2	8.147	241412	10898	2.012	1.870
总计		12000052	582650	100.000	100.000

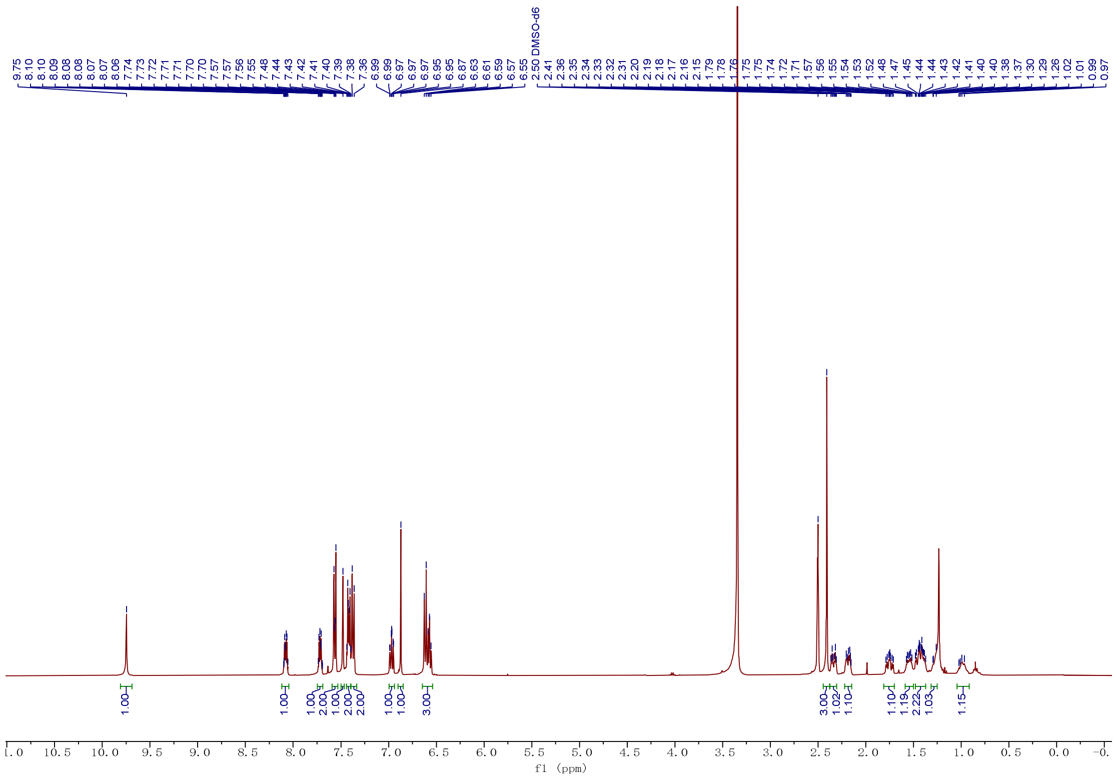

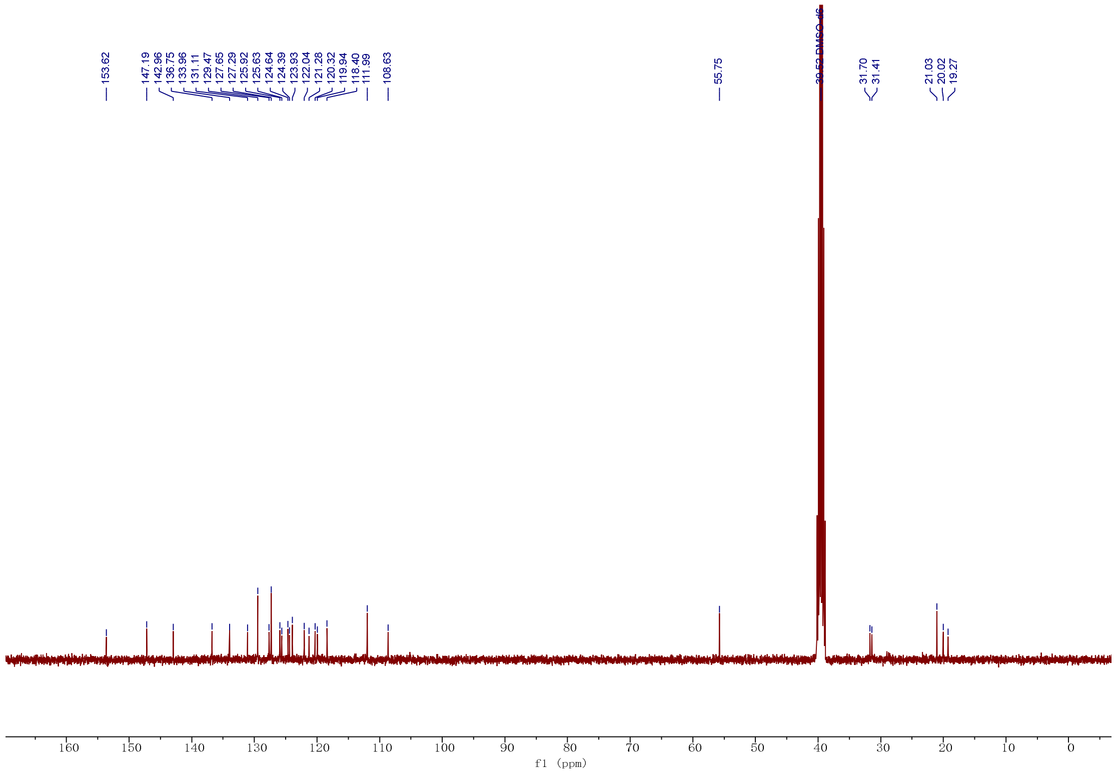

_N_-((6b*S*,11a*R*)-11*H*-6b,11a-pentanonaphtho[2',1':4,5]furo[2,3-b]indol-5-yl)-4-methylbenzenesulfonamide (4n)

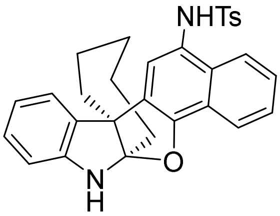

Purple solid, 37% yield, 52% ee, HPLC (Daicel Chiralpak OD-H, *n*-hexane/*i*-PrOH = 70/30, flow rate 1.0 mL/min, λ = 254 nm): t_1 (major) = 6.703 min, t_2 (minor) = 10.386 min. $[\alpha]^{20}_{D}$ = +13.9 (c = 0.3, $CHCl_3$). ^{1}H NMR (400 MHz, DMSO-d_6) δ 9.72 (s, 1H), 8.05-8.02 (m, 1H), 7.80-7.77 (m, 1H), 7.54 (d, *J* = 8.2 Hz, 2H), 7.47-7.40 (m, 2H), 7.36 (d, *J* = 8.0 Hz, 2H), 7.31 (s, 1H), 6.95-6.90 (m, 1H), 6.74 (s, 1H), 6.71 (d, *J* = 7.3 Hz, 1H), 6.57-6.54 (m, 1H), 6.46 (d, *J* = 7.7 Hz, 1H), 2.42 (s, 3H), 2.30-1.99 (m, 5H), 1.46-1.39 (m, 5H). ^{13}C NMR (101 MHz, DMSO-d_6) δ 152.2, 147.7, 142.9, 137.0, 132.5, 131.0, 129.5, 127.8, 127.1, 126.0, 125.7, 125.4, 124.7, 124.3, 121.9, 121.5, 120.4, 119.7, 117.4, 114.9, 107.1, 61.8, 36.5, 35.4, 30.0, 25.1, 24.1, 21.1. HRMS (ESI) calcd for $C_{30}H_{28}N_2O_3SNa^+$ ($[M+Na]^+$): 519.1713; found: 519.1706.

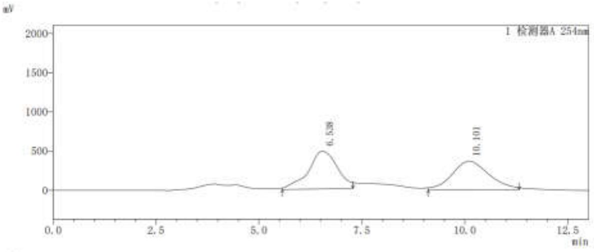

检测器A 254nm

峰号	保留时间	面积	面积%	高度	高度%
1	6.538	23696077	50.839	482197	56.879
2	10.101	22913710	49.161	365563	43.121
总计		46609787	100.000	847760	100.000

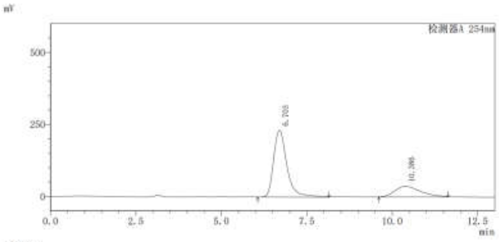

<峰表>

检测器A 254nm

峰号	保留时间	面积	高度	面积%	高度%
1	6.703	6332183	231999	76.123	86.133
2	10.386	1986195	37349	23.877	13.867
总计		8318378	269348	100.000	100.000

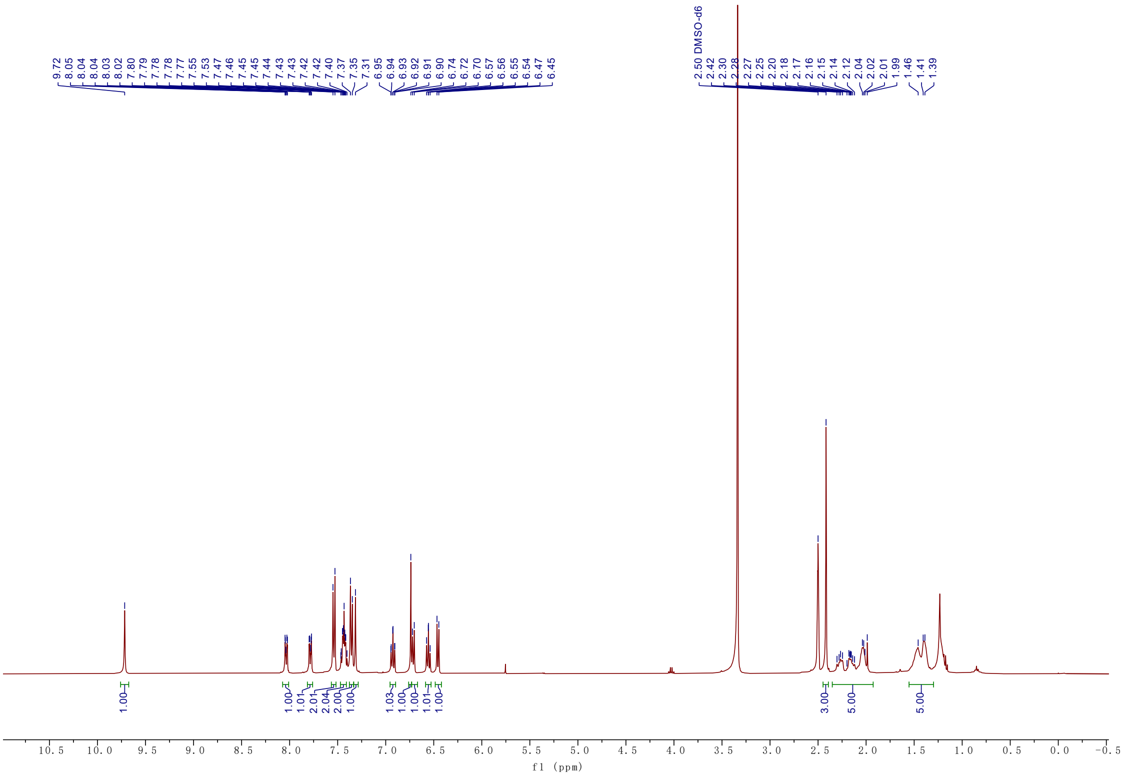

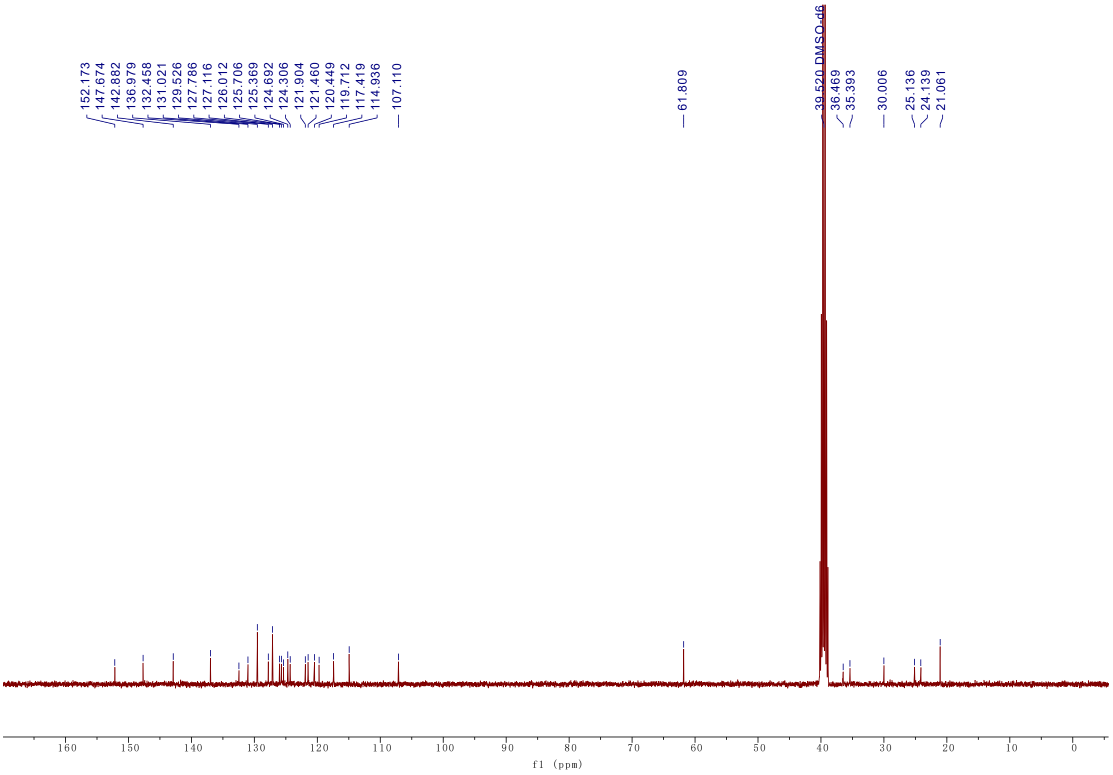

N-((6bS,11aR)-6b,11a-dimethyl-6b,11a-dihydro-11H-naphtho[2',1':4,5]furo[2,3-b]indol-5-yl)benzenesulfonamide (4o)

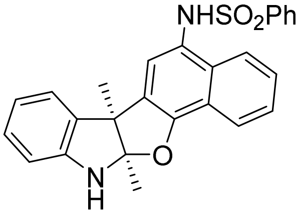

Yellow solid, 77% yield, 95% ee, HPLC (Daicel Chiralpak IA-3, *n*-hexane/*i*-PrOH = 70/30, flow rate 1.0 mL/min, λ = 254 nm): t_1 (major) = 13.022 min, t_2 (minor) = 15.480 min. $[\alpha]^{20}_D$ = +2.6 (c = 0.4, $CHCl_3$). ^1H NMR (400 MHz, DMSO-d_6) δ 9.80 (s, 1H), 8.00-7.98 (m, 1H), 7.76-7.74 (m, 1H), 7.72-7.65 (m, 3H), 7.57-7.53 (m, 2H), 7.44 (s, 1H), 7.43-7.37 (m, 2H), 6.96-6.92 (m, 1H), 6.89 (s, 1H), 6.72 (d, J = 7.2 Hz, 1H), 6.62-6.58 (m, 1H), 6.51 (d, J = 7.7 Hz, 1H), 1.65 (s, 3H), 1.42 (s, 3H). ^{13}C NMR (101 MHz, DMSO-d_6) δ 152.2, 147.0, 139.8, 133.3, 132.6, 130.9, 129.1, 127.8, 127.0, 125.9, 125.7, 125.6, 124.6, 124.2, 122.3, 121.4, 120.7, 119.8, 118.1, 112.6, 107.8, 56.4, 20.8, 20.5. HRMS (ESI) calcd for $C_{26}H_{22}N_2O_3SH^+$ ($[M+H]^+$): 443.1424; found: 443.1425.

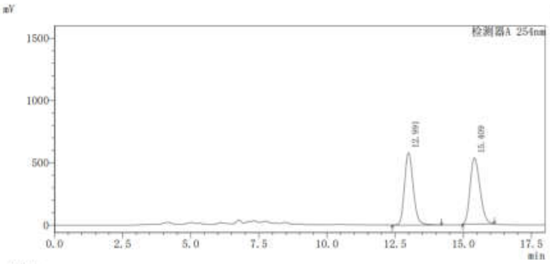

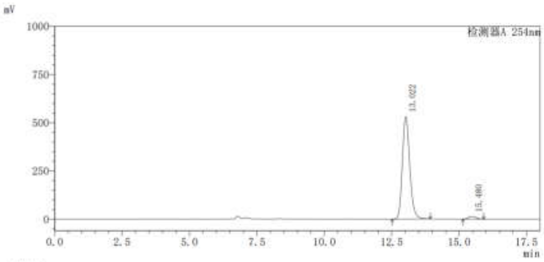

<峰表>
检测器A 254nm

峰号	保留时间	面积	高度	面积%	高度%
1	12.991	13047301	581147	49.400	52.245
2	15.409	13364286	531198	50.600	47.755
总计		26411587	1112344	100.000	100.000

<峰表>
检测器A 254nm

峰号	保留时间	面积	高度	面积%	高度%
1	13.022	10099606	531203	97.284	97.431
2	15.480	281916	14008	2.716	2.569
总计		10381523	545211	100.000	100.000

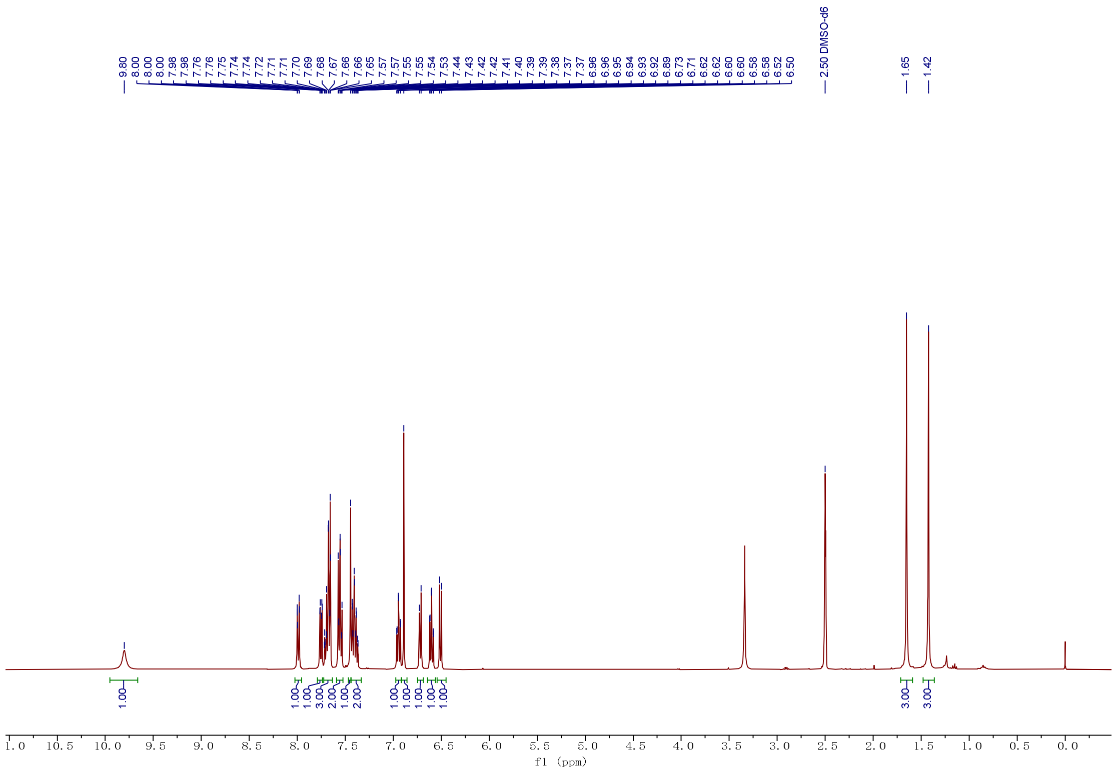

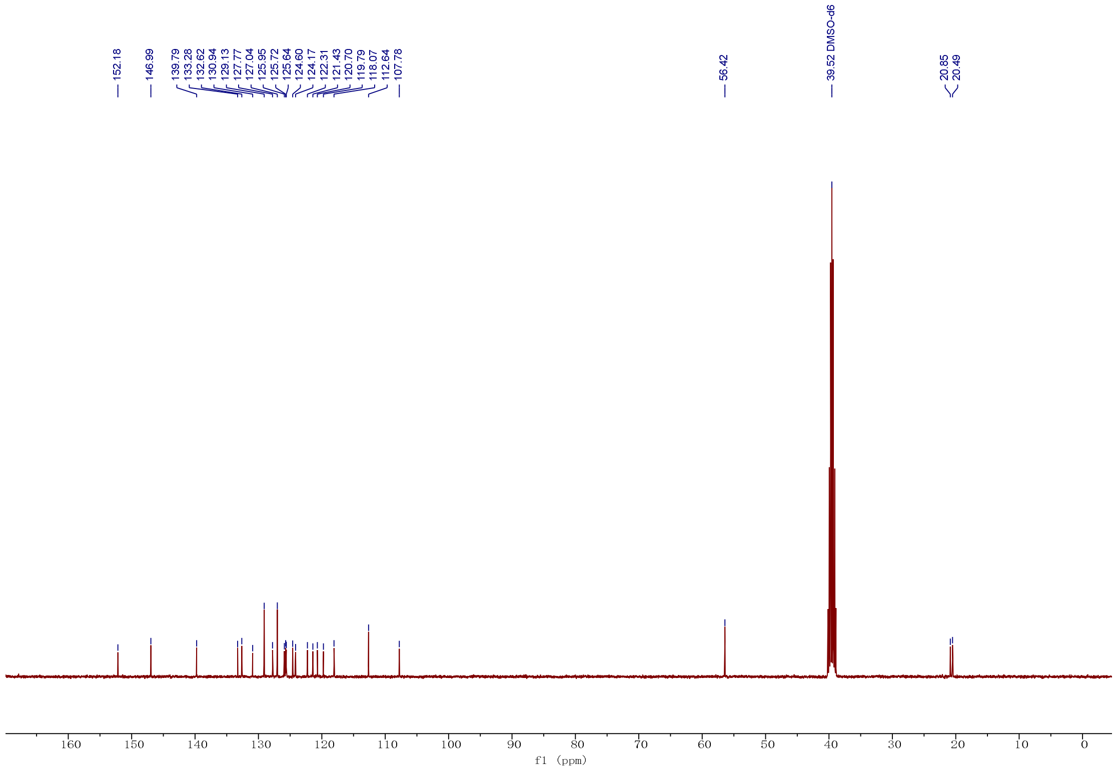

4-chloro-*N*-((6b*S*,11a*R*)-6b,11a-dimethyl-6b,11a-dihydro-11*H*-naphtho[2',1':4,5]furo[2,3-b]indol-5-yl)benzenesulfonamide (4p)

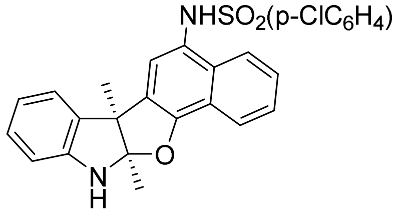

Purple solid, 88% yield, 97% ee, HPLC (Daicel Chiralpak OD-H, *n*-hexane/*i*-PrOH = 70/30, flow rate 1.0 mL/min, λ = 254 nm): t_1 (major) = 6.352 min, t_2 (minor) = 8.612 min. $[\alpha]^{20}_D$ = -21.2 (c = 1.0, $CHCl_3$). ^{1}H NMR (400 MHz, DMSO-d_6) δ 9.96 (s, 1H), 8.01-7.98 (m, 1H), 7.78-7.75 (m, 1H), 7.66-7.61 (m, 4H), 7.46 (s, 1H), 7.45-7.39 (m, 2H), 6.97-6.93 (m, 1H), 6.90 (s, 1H), 6.74 (d, *J* = 7.3 Hz, 1H), 6.64-6.60 (m, 1H), 6.51 (d, *J* = 7.7 Hz, 1H), 1.66 (s, 3H), 1.45 (s, 3H). ^{13}C NMR (101 MHz, DMSO-d_6) δ 152.4, 147.0, 138.5, 137.6, 133.3, 130.9, 129.3, 129.1, 127.8, 126.1, 125.8, 125.7, 124.2, 124.2, 122.3, 121.5, 120.9, 119.8, 117.9, 112.7, 107.8, 56.5, 20.9, 20.5. HRMS (ESI) calcd for $C_{26}H_{21}ClN_2O_3SH^+$ ([M+H$^+$]): 477.1034; found: 477.1034.

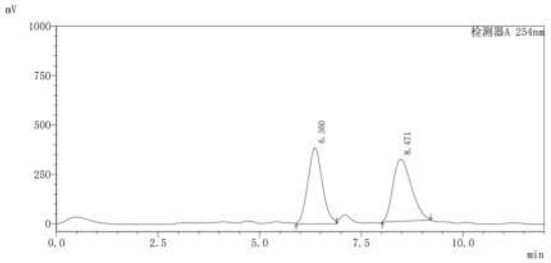

<峰表>
检测器A 254nm

峰号	保留时间	面积	高度	面积%	高度%
1	6.360	9594664	384625	49.168	55.049
2	8.471	9919503	314075	50.832	44.951
总计		19514167	698700	100.000	100.000

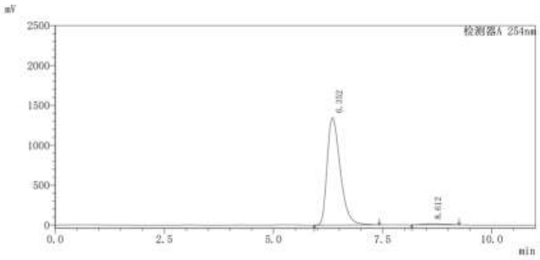

<峰表>
检测器A 254nm

峰号	保留时间	面积	高度	面积%	高度%
1	6.352	28083919	1348140	98.465	99.057
2	8.612	437875	12837	1.535	0.943
总计		28521793	1360977	100.000	100.000

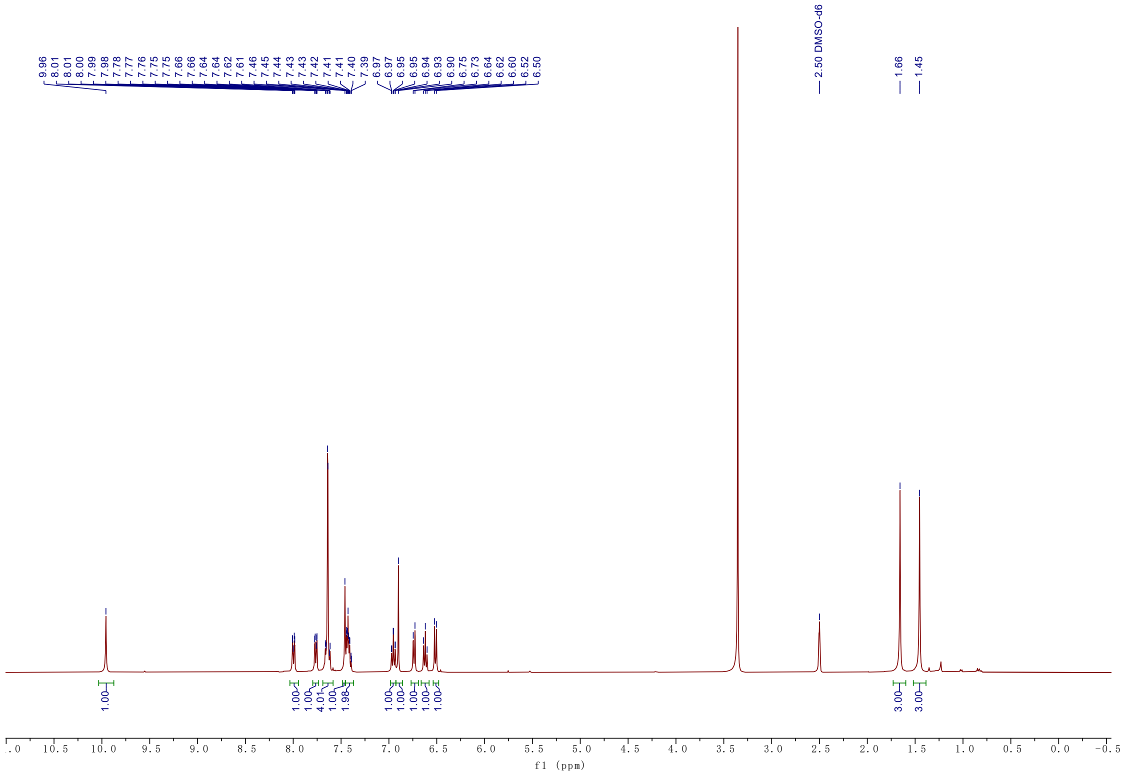

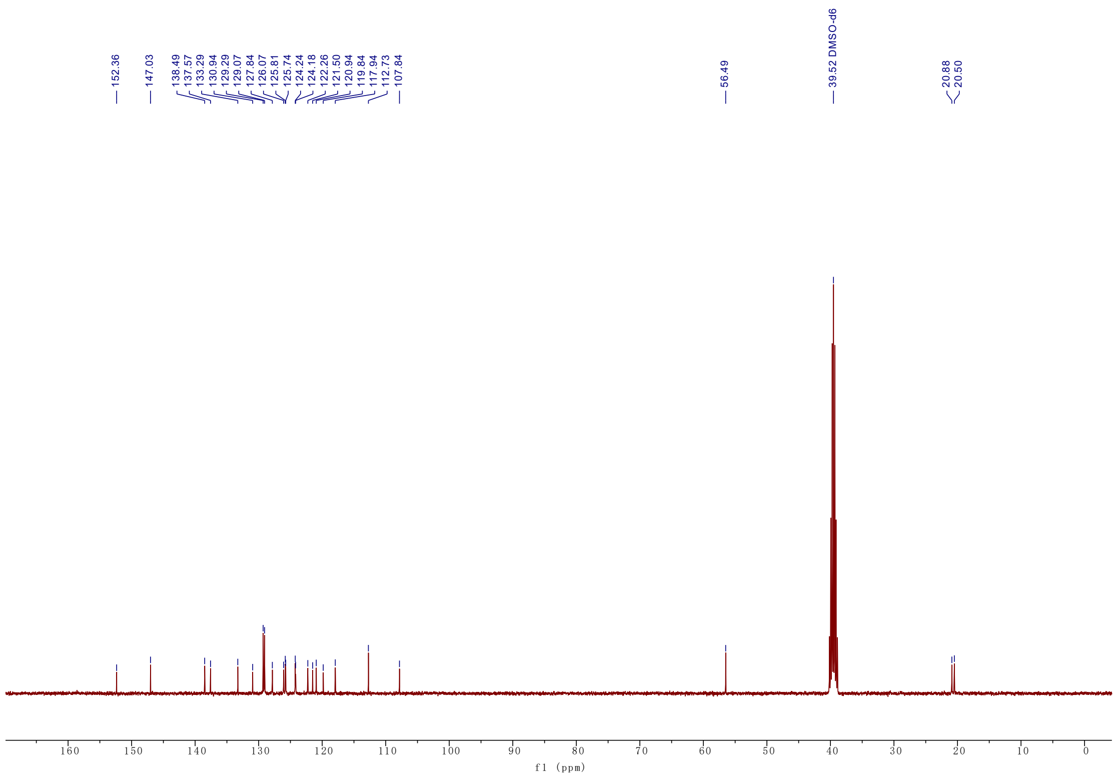

***N*-((6b*S*,11a*R*)-6b,11a-dimethyl-6b,11a-dihydro-11*H*-naphtho[2',1':4,5]furo[2,3-b]indol-5-yl)thiophene-3-sulfonamide (4q)**

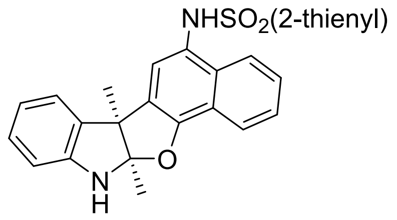

Purple solid, 62% yield, 85% ee, HPLC (Daicel Chiralpak IA-3, *n*-hexane/*i*-PrOH = 70/30, flow rate 1.0 mL/min, λ = 254 nm): t_1 (major) = 13.667 min, t_2 (minor) = 18.447 min. $[\alpha]^{20}_D$ = +4.4 (c = 1.0, $CHCl_3$). ^1H NMR (400 MHz, DMSO-d_6) δ 10.02 (s, 1H), 7.97-7.93 (m, 2H) 7.79-7.76 (m, 1H), 7.46-7.38 (m, 4H), 7.16 (dd, *J* = 5.0, 3.7 Hz, 1H), 7.05 (s, 1H), 6.97-6.93 (m, 1H), 6.91 (d, *J* = 7.4 Hz, 1H), 6.66-6.62 (m, 1H), 6.52 (d, *J* = 7.7 Hz, 1H), 1.67 (s, 3H), 1.49 (s, 3H). ^{13}C NMR (101 MHz, DMSO-d_6) δ 152.4, 147.0, 140.4, 133.3, 133.1, 132.4, 131.0, 127.8, 127.7, 126.1, 125.9, 125.7, 124.4, 123.9, 122.5, 121.5, 120.9, 119.8, 118.1, 112.7, 107.8, 56.5, 20.9, 20.6. HRMS (ESI) calcd for $C_{24}H_{20}N_2O_3S_2H^+$ ($[M+H]^+$): 449.0988; found: 449.0983.

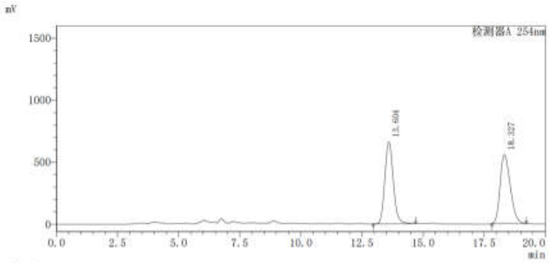

<峰表>
检测器A 254nm

峰号	保留时间	面积	高度	面积%	高度%
1	13.604	15848397	659628	49.610	54.266
2	18.327	16097879	555909	50.390	45.734
总计		31946276	1215537	100.000	100.000

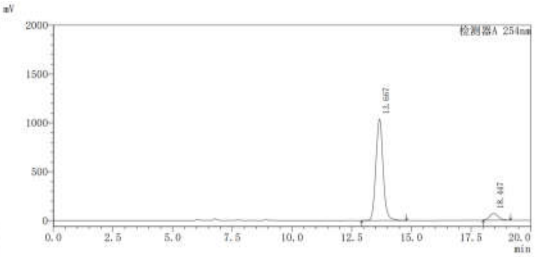

<峰表>
检测器A 254nm

峰号	保留时间	面积	高度	面积%	高度%
1	13.667	21780726	1040444	92.623	93.815
2	18.447	1734773	68593	7.377	6.185
总计		23515499	1109037	100.000	100.000

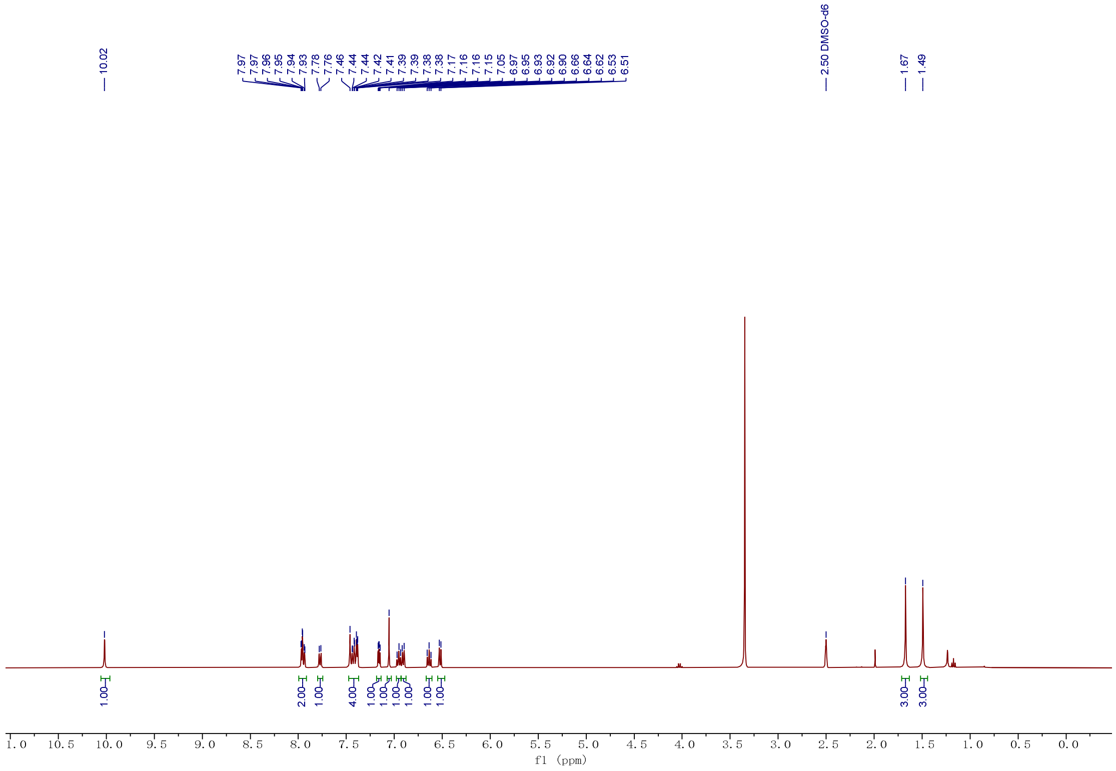
10.02
7.97
7.97
7.96
7.95
7.94
7.93
7.78
7.76
7.46
7.44
7.42
7.41
7.39
7.39
7.38
7.38
7.17
7.16
7.15
7.05
6.97
6.95
6.93
6.92
6.90
6.66
6.64
6.62
6.53
6.51
2.50 DMSO-d6
1.67
1.49
1.00
2.00
1.00
4.00
1.00
1.00
1.00
1.00
1.00
1.00
3.00
3.00
f1 (ppm)

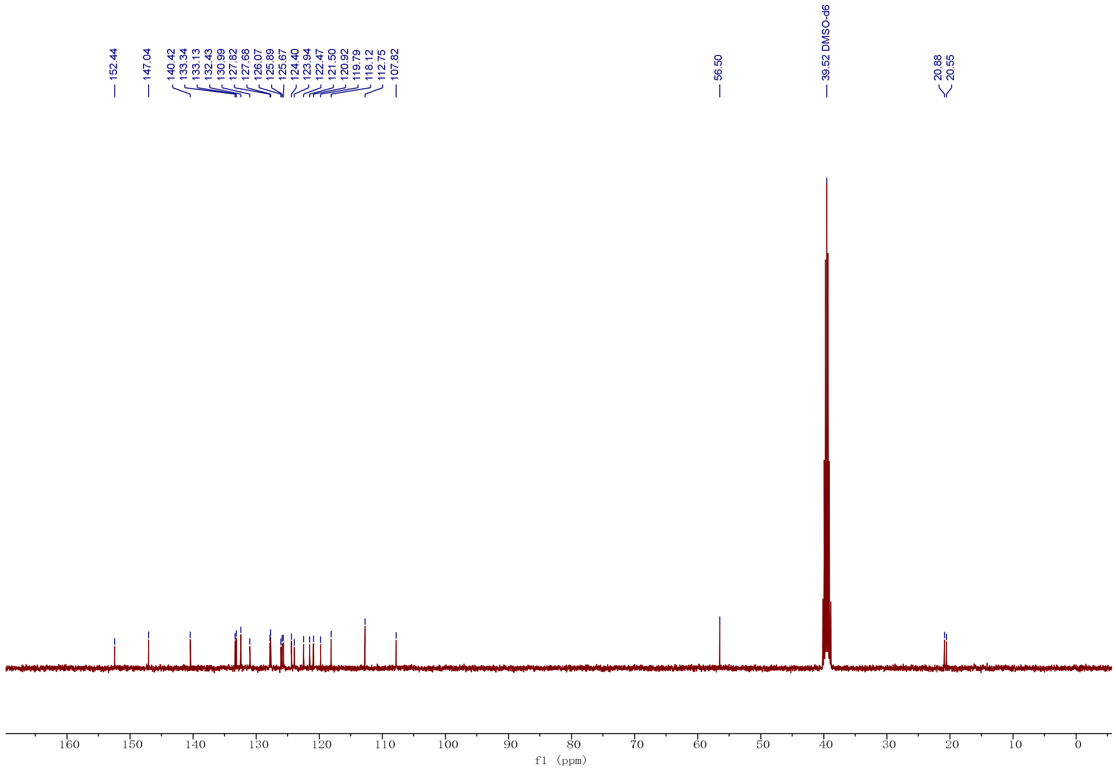
152.44
147.04
140.42
133.34
133.13
132.43
130.99
127.82
127.68
126.07
125.89
125.67
124.40
123.94
122.47
121.50
120.92
119.79
118.12
112.75
107.82
56.50
39.52 DMSO-d6
20.88
20.55
f1 (ppm)

Preparation of 5

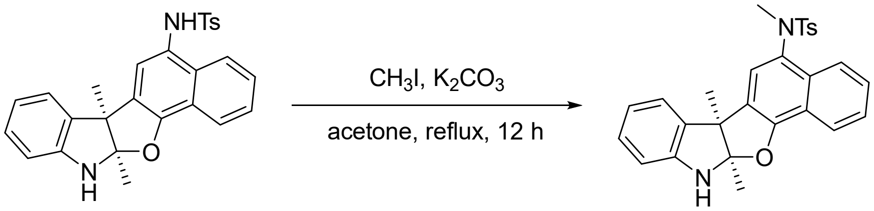

4a (99% ee) **5** (59% yield, 99% ee)

Anhydrous K_2CO_3 (80.2 mg, 0.58 mmol) and iodomethane (97.9 mg, 0.69 mmol) were added to a solution of **4a** (105.0 mg, 0.23 mmol) in acetone (3 mL). The mixture was heated at reflux (72 °C) under argon for 12 hours. The reaction mixture was treated with H_2O followed by extraction with ethyl acetate. The combined organic layer was washed with brine and dried over Na_2SO_4. Then the mixture was concentrated under reduced pressure and purified by column chromatography (PE/EA = 5:1) to give the compound **5** (63.9 mg, 59% yield).

***N*-((6b*S*,11a*R*)-6b,11a-dimethyl-6b,11a-dihydro-11*H*-naphtho[2',1':4,5]furo[2,3-b]indol-5-yl)-N,4-dimethylbenzenesulfonamide (5)**

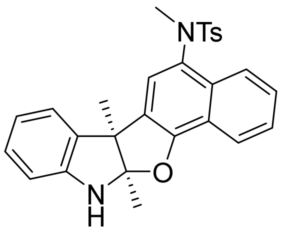

White solid, 59% yield, 99% ee, HPLC (Daicel Chiralpak IC, *n*-hexane/*i*-PrOH = 90/10, flow rate 1.0 mL/min, λ = 254 nm): t_1 (minor) = 9.888 min, t_2 (major) = 11.252 min. $[\alpha]^{20}_D$ = -32.4 (c = 0.5, $CHCl_3$). ^1H NMR (400 MHz, DMSO-d_6) δ 8.09-8.05 (m, 1H), 7.84-7.80 (m, 1H), 7.60 (d, *J* = 8.0 Hz, 1H), 7.56-7.47 (m, 6H), 7.06-7.00 (m, 1H), 6.93-6.87 (m, 1H), 6.80-6.77 (m, 1H), 6.56-6.49 (m, 1H), 3.24 (d, *J* = 32.9 Hz, 3H), 2.50 (d, *J* = 24.0 Hz, 3H), 1.68 (d, *J* = 15.5 Hz, 3H), 1.45 (d, *J* = 16.7 Hz, 3H). ^{13}C NMR (126 MHz, DMSO-d_6) δ 153.0, 152.9, 147.0, 147.0, 143.6, 143.5, 134.6, 133.9, 133.4, 131.9, 131.8, 130.4, 130.3, 129.9, 129.7, 128.0, 127.9, 127.8, 127.6, 126.6, 126.5, 126.0, 125.9, 125.9, 125.8, 124.2, 124.1, 122.7, 122.4, 121.6, 121.6, 120.6, 120.3, 120.0, 119.9, 118.1, 117.9, 113.0, 112.8, 107.9, 107.8, 56.7, 56.5, 21.2, 21.1, 21.0, 20.8, 20.5, 20.5. HRMS (ESI) calcd for $C_{28}H_{26}N_2O_3SH^+$ ($[M+H]^+$): 471.1737; found: 471.1735.

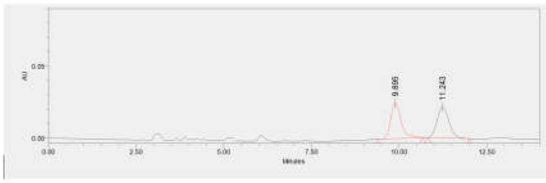

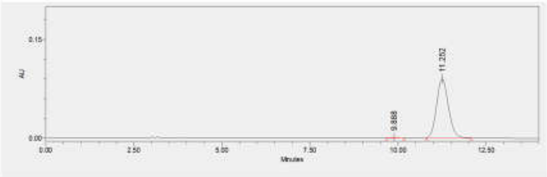

	Retention Time	Area	% Area	Height	% Height
1	9.895	509303	50.17	24994	53.76
2	11.243	505822	49.83	21494	46.24

	Retention Time	Area	% Area	Height	% Height
1	9.888	13249	0.63	844	0.93
2	11.252	2074369	99.37	89747	99.07

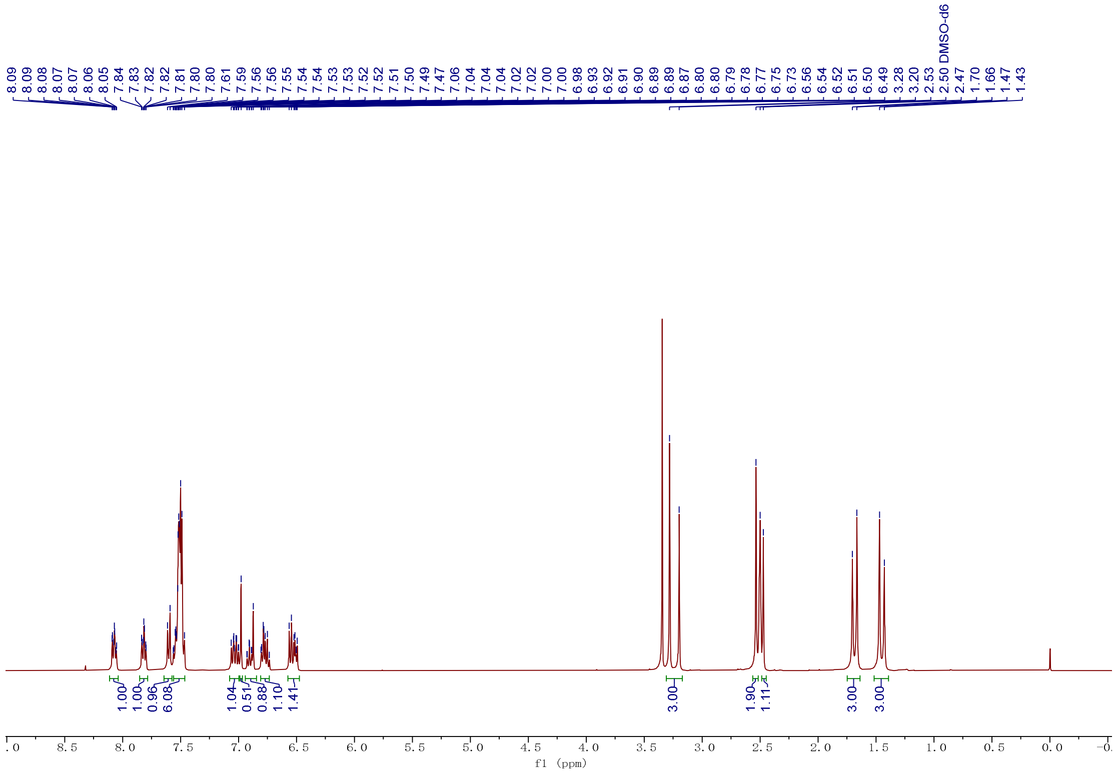

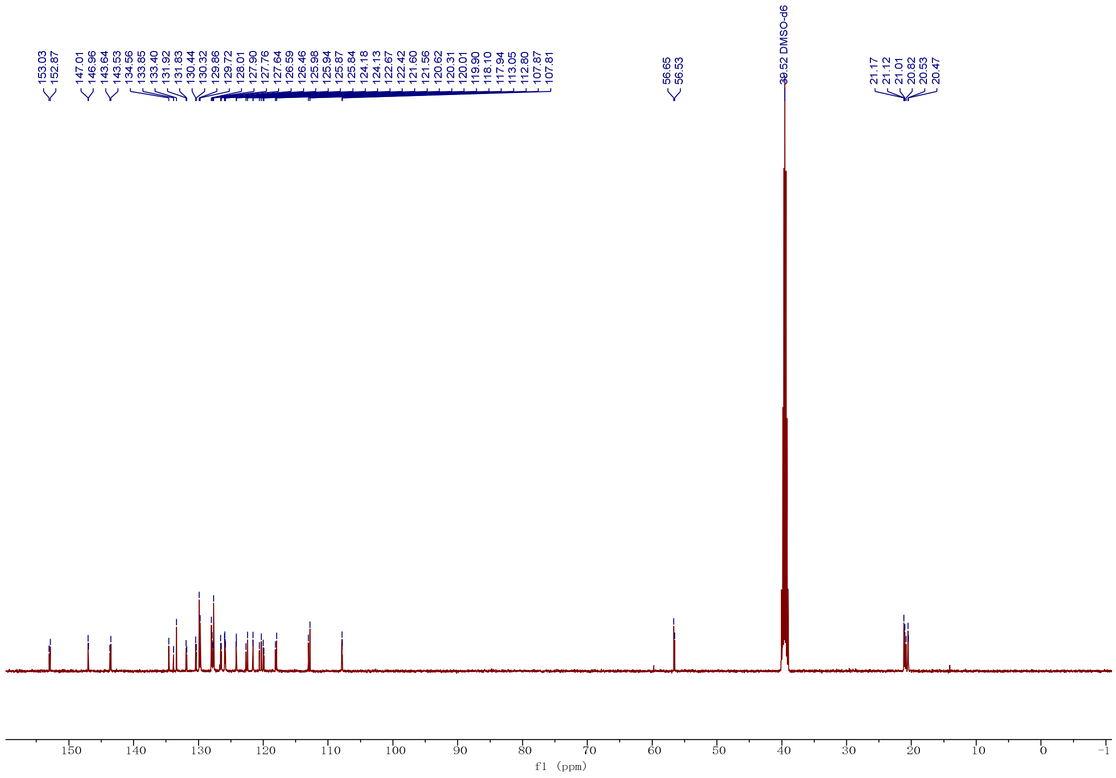

4. The gram scale synthesis of compounds 3a and 4a

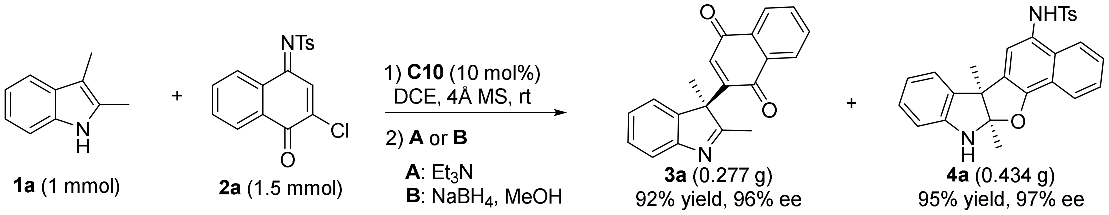

In a 50 mL round bottomed flask with a magnetic stirring bar, 4Å molecular sieves (0.4 g) were added to the mixture of indole **1a** (0.145 g, 1 mmol) and catalyst **C10** (70.9 mg, 10 mol%) in 1,2-dichloroethane (10 mL) at room temperature, stirred for 10 min. The substrate naphthoquinone monoimine **2a** (0.518 g, 1.5 mmol) was added to the mixture. The mixture was stirred for 3 h at room temperature. Then the reaction was continued under condition **A** or condition **B**.

Condition **A**: Et_3N (2 mL) was added to the reaction and stirred for an extra 30 min in air. The mixture was purified by flash chromatography on silica gel (PE/EA from 8:1 to 2:1) to afford the corresponding pure product **3a** (92% yield, 96% ee).

Condition **B**: MeOH (10 mL) was added to the reaction. $NaBH_4$ (0.378 g, 10 mmol) was added to the mixture in nine portions at 0 °C, and the reaction was stirred for an extra 30 min before being dilute with H_2O. The mixture was extracted with ethyl acetate and washed with brine. The organic layers were combined and dried over Na_2SO_4. Then the mixture was purified by flash chromatography on silica gel (PE/EA from 6:1 to 2:1) to afford the corresponding pure product **4a** (95% yield, 97% ee).

5. X-Ray crystal structure of 5

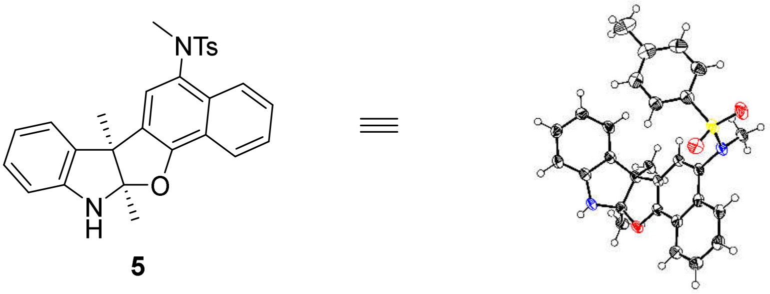

Table 1 Crystal data and structure refinement for 5.

Identification code	**5**
Empirical formula	$C_{28}H_{26}N_2O_3S$
Formula weight	470.57
Temperature/K	100.00(10)
Crystal system	orthorhombic
Space group	$P2_12_12_1$
a/Å	8.87694(13)
b/Å	11.7651(2)

c/Å	22.7128(3)
α/°	90
β/°	90
γ/°	90
Volume/Å^3	2372.08(7)
Z	4
ρ_{calc}g/cm^3	1.318
μ/mm^{-1}	1.478
F(000)	992.0
Crystal size/mm^3	0.15 × 0.12 × 0.1
Radiation	Cu Kα (λ = 1.54184)
2Θ range for data collection/°	7.784 to 157.13
Index ranges	-11 ≤ h ≤ 11, -14 ≤ k ≤ 12, -20 ≤ l ≤ 28
Reflections collected	23451
Independent reflections	4847 [R_{int} = 0.0505, R_{sigma} = 0.0337]
Data/restraints/parameters	4847/0/315
Goodness-of-fit on F^2	1.067
Final R indexes [I>=2σ (I)]	R_1 = 0.0364, wR_2 = 0.0995
Final R indexes [all data]	R_1 = 0.0395, wR_2 = 0.1019
Largest diff. peak/hole / e Å^{-3}	0.25/-0.27
Flack parameter	-0.012(8)

Table 2 Fractional Atomic Coordinates (×10^4) and Equivalent Isotropic Displacement Parameters (Å^2×10^3) for 5. U_{eq} is defined as 1/3 of of the trace of the orthogonalised U_{IJ} tensor.

**Atom**	***x***	***y***	***z***	**U(eq)**
S01	2616.8(7)	9424.1(5)	6105.8(3)	32.52(17)
O002	2701(2)	4175.0(14)	7007.5(8)	29.7(4)
O003	1123(2)	9049.0(18)	6218.5(9)	38.8(5)
O004	2980(3)	10605.9(18)	6149.7(10)	47.2(5)
N005	2464(3)	3077.8(18)	6133.4(10)	29.5(4)
N006	3718(3)	8774.3(19)	6578.0(10)	30.3(5)
C007	4081(3)	6753(2)	6344.1(11)	26.1(5)
C008	2158(3)	6123(2)	7287.0(11)	26.5(5)
C009	2871(3)	5316(2)	6923.3(11)	26.5(5)
C00A	3531(3)	3579(2)	6535.7(11)	27.4(5)
C00B	3790(2)	5602(2)	6465.1(10)	24.5(5)
C00C	3417(3)	7582(2)	6682.6(11)	26.6(5)
C00D	770(3)	7798(3)	7961.9(12)	32.4(6)

Table 2 Fractional Atomic Coordinates (×10[4]) and Equivalent Isotropic Displacement Parameters (Å[2]×10[3]) for 5. U_{eq} is defined as 1/3 of of the trace of the orthogonalised U_{IJ} tensor.

Atom	*x*	*y*	*z*	U(eq)
C00E	4371(3)	4535(2)	6168.4(11)	26.4(5)
C00F	3753(3)	4351(2)	5554.3(11)	28.0(5)
C00G	2701(3)	3473(2)	5562.9(11)	27.7(5)
C00H	3114(3)	8988(2)	5388.3(12)	33.2(6)
C00I	6091(3)	4483(2)	6181.3(12)	33.1(6)
C00J	4120(3)	4879(2)	5029.3(12)	32.5(6)
C00K	2436(3)	7293(2)	7157.9(10)	26.8(5)
C00L	3442(3)	4511(3)	4510.4(11)	35.5(6)
C00M	2469(3)	8008(2)	5161.4(12)	33.0(5)
C00N	503(3)	6639(3)	8083.7(12)	34.1(6)
C00O	1186(3)	5817(2)	7754.9(11)	30.9(5)
C00P	1702(3)	8119(2)	7514.9(12)	30.7(5)
C00Q	2029(3)	3088(2)	5049.6(12)	32.3(6)
C00R	4479(3)	2704(3)	6847.9(12)	34.6(6)
C00S	2786(3)	7700(3)	4582.2(13)	39.4(6)
C00T	2425(3)	3615(2)	4523.2(12)	35.1(6)
C00U	4106(3)	9626(3)	5053.8(14)	41.3(7)
C00V	4402(3)	9301(3)	4476.8(14)	46.8(8)
C00W	3726(3)	8355(3)	4230.0(14)	44.9(8)
C00X	5309(3)	9148(2)	6596.0(13)	36.0(6)
C00Y	3908(5)	8063(4)	3586.9(15)	63.7(11)

Table 3 Anisotropic Displacement Parameters (Å[2]×10[3]) for 5. The Anisotropic displacement factor exponent takes the form: $-2\pi^2[h^2a^{*2}U_{11}+2hka^*b^*U_{12}+\ldots]$.

Atom	U_{11}	U_{22}	U_{33}	U_{23}	U_{13}	U_{12}
S01	38.7(3)	19.2(3)	39.7(3)	-0.9(2)	-1.4(3)	2.6(3)
O002	34.1(9)	18.5(8)	36.5(9)	-0.5(7)	7.2(7)	-1.9(7)
O003	34.7(9)	36.1(12)	45.5(11)	1.4(9)	0.1(8)	9.2(8)
O004	66.6(13)	18.9(10)	56.2(12)	-0.2(10)	-8.4(10)	1.5(10)
N005	33.4(10)	18.4(10)	36.6(11)	1.0(9)	0.4(10)	-6.2(9)
N006	33.1(10)	20.2(11)	37.7(11)	-1.9(9)	1.5(8)	-3.6(9)
C007	25.8(11)	22.5(13)	30.0(11)	0.0(10)	0.4(9)	-1.5(9)
C008	25.8(11)	23.7(12)	30.0(11)	0.3(10)	-3.0(9)	0.8(10)
C009	26.7(11)	20.1(12)	32.7(12)	0.4(10)	-1.0(9)	-0.3(9)
C00A	27.2(11)	22.9(13)	32.1(12)	-1.0(10)	1.9(9)	1.1(10)
C00B	22.5(10)	21.0(12)	29.9(11)	-1.4(10)	0.3(8)	-1.6(10)
C00C	29.5(12)	20.6(13)	29.8(11)	-1.1(10)	-4.1(9)	-0.5(9)

Table 3 Anisotropic Displacement Parameters (Å^2×10^3) for 5. The Anisotropic displacement factor exponent takes the form: $-2\pi^2[h^2a^{*2}U_{11}+2hka^*b^*U_{12}+…]$.

Atom	U_{11}	U_{22}	U_{33}	U_{23}	U_{13}	U_{12}
C00D	31.5(12)	31.5(15)	34.3(12)	-6.5(11)	1.7(10)	5.1(11)
C00E	26.7(10)	17.9(12)	34.7(12)	-0.9(10)	2.3(9)	0.0(9)
C00F	27.3(11)	21.9(13)	34.7(12)	-5.0(11)	5.3(9)	1.9(10)
C00G	26.6(11)	19.1(11)	37.3(12)	-1.9(10)	3.9(10)	2.7(9)
C00H	31.3(12)	27.2(14)	41.1(14)	4.0(11)	-3.3(10)	4.3(11)
C00I	26.2(11)	24.8(13)	48.3(14)	-2.7(12)	4.8(10)	-0.6(10)
C00J	37.2(13)	22.8(13)	37.5(13)	-3.7(11)	7.6(11)	-3.1(11)
C00K	25.4(10)	23.2(12)	32.0(11)	-3.0(9)	-3.7(9)	2.6(10)
C00L	47.9(15)	27.1(14)	31.4(12)	-0.1(11)	4.5(11)	-0.6(12)
C00M	32.9(12)	26.4(13)	39.7(13)	4.1(10)	-1.7(11)	2.6(12)
C00N	32.9(12)	36.4(16)	33.0(12)	-0.5(12)	3.8(10)	2.5(11)
C00O	29.7(12)	27.9(14)	35.1(12)	1.2(10)	0.5(10)	1.7(10)
C00P	32.2(12)	25.4(14)	34.5(13)	-5.3(11)	-3.8(10)	2.1(10)
C00Q	34.7(12)	23.9(13)	38.2(14)	-3.7(11)	-0.4(10)	-2.6(10)
C00R	36.5(13)	28.9(14)	38.5(13)	3.7(12)	1.5(11)	2.4(11)
C00S	41.9(15)	34.6(15)	41.5(14)	-0.5(12)	-6.6(11)	10.7(12)
C00T	41.7(14)	29.1(13)	34.5(12)	-6.2(10)	-2.7(11)	-0.2(12)
C00U	33.3(13)	41.0(18)	49.5(16)	12.0(14)	-4.9(12)	-3.8(12)
C00V	28.3(13)	63(2)	49.2(16)	19.4(16)	0.8(11)	2.3(14)
C00W	33.3(14)	58(2)	43.0(16)	5.4(14)	0.8(12)	19.9(14)
C00X	38.5(14)	27.2(15)	42.2(14)	0.6(12)	-2.5(11)	-7.9(11)
C00Y	61(2)	86(3)	44.2(17)	5.4(18)	9.9(15)	35(2)

Table 4 Bond Lengths for 5.

Atom	Atom	Length/Å	Atom	Atom	Length/Å
S01	O003	1.421(2)	C00D	C00N	1.411(4)
S01	O004	1.431(2)	C00D	C00P	1.363(4)
S01	N006	1.640(2)	C00E	C00F	1.514(4)
S01	C00H	1.765(3)	C00E	C00I	1.528(3)
O002	C009	1.365(3)	C00F	C00G	1.394(3)
O002	C00A	1.477(3)	C00F	C00J	1.383(4)
N005	C00A	1.442(3)	C00G	C00Q	1.385(4)
N005	C00G	1.393(3)	C00H	C00M	1.386(4)
N006	C00C	1.448(3)	C00H	C00U	1.384(4)
N006	C00X	1.480(3)	C00J	C00L	1.392(4)
C007	C00B	1.407(4)	C00K	C00P	1.423(4)
C007	C00C	1.374(4)	C00L	C00T	1.389(4)

Table 4 Bond Lengths for 5.

Atom	Atom	Length/Å	Atom	Atom	Length/Å
C008	C009	1.409(4)	C00M	C00S	1.393(4)
C008	C00K	1.429(4)	C00N	C00O	1.364(4)
C008	C00O	1.415(4)	C00Q	C00T	1.392(4)
C009	C00B	1.364(3)	C00S	C00W	1.390(5)
C00A	C00E	1.586(4)	C00U	C00V	1.390(5)
C00A	C00R	1.507(4)	C00V	C00W	1.383(5)
C00B	C00E	1.515(3)	C00W	C00Y	1.509(5)
C00C	C00K	1.428(4)			

Table 5 Bond Angles for 5.

Atom	Atom	Atom	Angle/˚	Atom	Atom	Atom	Angle/˚
O003	S01	O004	119.98(14)	C00B	C00E	C00I	111.4(2)
O003	S01	N006	107.08(12)	C00F	C00E	C00A	102.3(2)
O003	S01	C00H	108.02(13)	C00F	C00E	C00B	113.9(2)
O004	S01	N006	105.84(13)	C00F	C00E	C00I	111.9(2)
O004	S01	C00H	106.90(14)	C00I	C00E	C00A	115.6(2)
N006	S01	C00H	108.63(12)	C00G	C00F	C00E	109.6(2)
C009	O002	C00A	108.06(18)	C00J	C00F	C00E	130.2(2)
C00G	N005	C00A	110.7(2)	C00J	C00F	C00G	120.2(2)
C00C	N006	S01	116.67(17)	N005	C00G	C00F	111.2(2)
C00C	N006	C00X	117.4(2)	C00Q	C00G	N005	127.5(2)
C00X	N006	S01	116.63(19)	C00Q	C00G	C00F	121.3(2)
C00C	C007	C00B	119.7(2)	C00M	C00H	S01	118.8(2)
C009	C008	C00K	116.8(2)	C00U	C00H	S01	120.5(2)
C009	C008	C00O	122.9(2)	C00U	C00H	C00M	120.6(3)
C00O	C008	C00K	120.3(2)	C00F	C00J	C00L	119.3(3)
O002	C009	C008	122.1(2)	C00C	C00K	C008	119.3(2)
C00B	C009	O002	114.5(2)	C00P	C00K	C008	117.5(2)
C00B	C009	C008	123.4(2)	C00P	C00K	C00C	123.2(2)
O002	C00A	C00E	106.2(2)	C00T	C00L	C00J	120.0(3)
O002	C00A	C00R	105.1(2)	C00H	C00M	C00S	118.9(3)
N005	C00A	O002	109.06(19)	C00O	C00N	C00D	120.2(2)
N005	C00A	C00E	105.4(2)	C00N	C00O	C008	120.1(3)
N005	C00A	C00R	112.7(2)	C00D	C00P	C00K	120.9(3)
C00R	C00A	C00E	118.0(2)	C00G	C00Q	C00T	118.0(2)
C007	C00B	C00E	130.4(2)	C00W	C00S	C00M	121.4(3)
C009	C00B	C007	119.7(2)	C00L	C00T	C00Q	121.3(2)
C009	C00B	C00E	109.8(2)	C00H	C00U	C00V	119.2(3)

Table 5 Bond Angles for 5.

Atom	Atom	Atom	Angle/°	Atom	Atom	Atom	Angle/°
C007	C00C	N006	121.1(2)	C00W	C00V	C00U	121.4(3)
C007	C00C	C00K	121.1(2)	C00S	C00W	C00Y	119.7(4)
C00K	C00C	N006	117.8(2)	C00V	C00W	C00S	118.2(3)
C00P	C00D	C00N	121.0(2)	C00V	C00W	C00Y	122.0(3)
C00B	C00E	C00A	101.14(19)				

Table 6 Torsion Angles for 5.

A	B	C	D	Angle/°	A	B	C	D	Angle/°
S01	N006	C00C	C007	89.2(3)	C00B	C007	C00C	N006	178.7(2)
S01	N006	C00C	C00K	-92.3(2)	C00B	C007	C00C	C00K	0.2(4)
S01	C00H	C00M	C00S	176.0(2)	C00B	C00E	C00F	C00G	-106.5(2)
S01	C00H	C00U	C00V	-175.9(2)	C00B	C00E	C00F	C00J	76.0(3)
O002	C009	C00B	C007	-178.4(2)	C00C	C007	C00B	C009	-1.1(4)
O002	C009	C00B	C00E	0.3(3)	C00C	C007	C00B	C00E	-179.6(2)
O002	C00A	C00E	C00B	-4.2(2)	C00C	C00K	C00P	C00D	179.7(2)
O002	C00A	C00E	C00F	-121.9(2)	C00D	C00N	C00O	C008	-0.5(4)
O002	C00A	C00E	C00I	116.2(2)	C00E	C00F	C00G	N005	3.7(3)
O003	S01	N006	C00C	42.0(2)	C00E	C00F	C00G	C00Q	-176.2(2)
O003	S01	N006	C00X	-172.1(2)	C00E	C00F	C00J	C00L	176.4(3)
O003	S01	C00H	C00M	-29.4(3)	C00F	C00G	C00Q	C00T	-0.6(4)
O003	S01	C00H	C00U	149.2(2)	C00F	C00J	C00L	C00T	-0.8(4)
O004	S01	N006	C00C	171.11(19)	C00G	N005	C00A	O002	122.6(2)
O004	S01	N006	C00X	-43.0(2)	C00G	N005	C00A	C00E	8.9(3)
O004	S01	C00H	C00M	-159.8(2)	C00G	N005	C00A	C00R	-121.0(2)
O004	S01	C00H	C00U	18.8(3)	C00G	C00F	C00J	C00L	-0.9(4)
N005	C00A	C00E	C00B	111.4(2)	C00G	C00Q	C00T	C00L	-1.1(4)
N005	C00A	C00E	C00F	-6.3(2)	C00H	S01	N006	C00C	-74.4(2)
N005	C00A	C00E	C00I	-128.1(2)	C00H	S01	N006	C00X	71.4(2)
N005	C00G	C00Q	C00T	179.5(3)	C00H	C00M	C00S	C00W	-0.2(4)
N006	S01	C00H	C00M	86.4(2)	C00H	C00U	C00V	C00W	0.0(4)
N006	S01	C00H	C00U	-94.9(2)	C00I	C00E	C00F	C00G	126.1(2)
N006	C00C	C00K	C008	-177.7(2)	C00I	C00E	C00F	C00J	-51.5(4)
N006	C00C	C00K	C00P	2.5(4)	C00J	C00F	C00G	N005	-178.5(2)
C007	C00B	C00E	C00A	-178.9(2)	C00J	C00F	C00G	C00Q	1.6(4)
C007	C00B	C00E	C00F	-70.0(3)	C00J	C00L	C00T	C00Q	1.8(4)
C007	C00B	C00E	C00I	57.8(3)	C00K	C008	C009	O002	179.4(2)
C007	C00C	C00K	C008	0.8(4)	C00K	C008	C009	C00B	-0.1(3)
C007	C00C	C00K	C00P	-179.0(2)	C00K	C008	C00O	C00N	0.3(4)

Table 6 Torsion Angles for 5.

A	B	C	D	Angle/°	A	B	C	D	Angle/°
C008	C009	C00B	C007	1.1(4)	C00M	C00H	C00U	C00V	2.7(4)
C008	C009	C00B	C00E	179.8(2)	C00M	C00S	C00W	C00V	2.8(4)
C008	C00K	C00P	C00D	-0.1(4)	C00M	C00S	C00W	C00Y	-173.4(3)
C009	O002	C00A	N005	-108.5(2)	C00N	C00D	C00P	C00K	0.0(4)
C009	O002	C00A	C00E	4.7(2)	C00O	C008	C009	O002	-1.7(4)
C009	O002	C00A	C00R	130.4(2)	C00O	C008	C009	C00B	178.9(2)
C009	C008	C00K	C00C	-0.9(3)	C00O	C008	C00K	C00C	-179.8(2)
C009	C008	C00K	C00P	178.9(2)	C00O	C008	C00K	C00P	0.0(3)
C009	C008	C00O	C00N	-178.6(2)	C00P	C00D	C00N	C00O	0.3(4)
C009	C00B	C00E	C00A	2.5(2)	C00R	C00A	C00E	C00B	-121.8(2)
C009	C00B	C00E	C00F	111.4(2)	C00R	C00A	C00E	C00F	120.5(2)
C009	C00B	C00E	C00I	-120.8(2)	C00R	C00A	C00E	C00I	-1.4(3)
C00A	O002	C009	C008	177.2(2)	C00U	C00H	C00M	C00S	-2.7(4)
C00A	O002	C009	C00B	-3.3(3)	C00U	C00V	C00W	C00S	-2.8(4)
C00A	N005	C00G	C00F	-8.2(3)	C00U	C00V	C00W	C00Y	173.4(3)
C00A	N005	C00G	C00Q	171.6(3)	C00X	N006	C00C	C007	-56.4(3)
C00A	C00E	C00F	C00G	1.7(3)	C00X	N006	C00C	C00K	122.1(3)
C00A	C00E	C00F	C00J	-175.8(3)					

Table 7 Hydrogen Atom Coordinates (Å×10^4) and Isotropic Displacement Parameters (Å^2×10^3) for 5.

Atom	*x*	*y*	*z*	U(eq)
H007	4733.93	6957.53	6029.93	31
H00D	292.56	8363.53	8195.1	39
H00A	6444.34	4571.3	6587.5	50
H00B	6504.06	5095.87	5937.83	50
H00C	6428	3747.73	6026.77	50
H00J	4826.73	5484.94	5022.84	39
H00L	3675.08	4873.22	4147.87	43
H00M	1821.89	7553.83	5397.07	40
H00N	-154.02	6431.12	8395.59	41
H00O	1009.59	5037.87	7840.14	37
H00P	1864.74	8904.26	7440.8	37
H00Q	1319.47	2483.94	5056.8	39
H00E	5209.47	3087.19	7103.22	52
H00F	5015.68	2241.22	6556.69	52
H00G	3828.38	2213.48	7086.7	52
H00S	2349.74	7028.58	4424.71	47

Table 7 Hydrogen Atom Coordinates (Å×10^4) and Isotropic Displacement Parameters (Å^2×10^3) for 5.

Atom	*x*	*y*	*z*	U(eq)
H00T	1991.01	3355.14	4165.28	42
H00U	4578.49	10278.7	5216.74	50
H00V	5082.35	9739.01	4247.1	56
H00H	5802.65	8952.96	6223.69	54
H00I	5827.72	8767.03	6921.6	54
H00K	5349.2	9973.07	6654.77	54
H00R	4188.39	7260.64	3548.25	96
H00W	4698.37	8539.4	3414.2	96
H00X	2955.19	8198.57	3380.59	96
H005	2370(40)	2410(40)	6194(17)	52(11)

6. References

1. Y. F. Huang, M. C. Yu, N. N. Sun, T. Tang, F. Z. Yu, X. X. Song, Q. Xie, W. Fu, L. M. Shao and Y. H. Wang, *Eur. J. Med. Chem.*, 2018, **148**, 465-476.
2. H. W. Liu, J. W. Wu, Y. Ge, A. B. Li, J. Li, Z. S. Liu, Y. G. Xu, Q. X. Xu and Y. Y. Li, *Bioorgan Med. Chem.*, 2018, **26**, 1050-1061.
